# High-Throughput Profiling of Cas12a Orthologues and Engineered Variants for Enhanced Genome Editing Activity

**DOI:** 10.3390/ijms222413301

**Published:** 2021-12-10

**Authors:** Dan Zhu, Junyi Wang, Di Yang, Jianzhong Xi, Juan Li

**Affiliations:** College of Engineering, Peking University, Beijing 100871, China; zhudanzd1991@pku.edu.cn (D.Z.); wangjunyi1994@pku.edu.cn (J.W.); coeyangdi@pku.edu.cn (D.Y.)

**Keywords:** gene editing, CRISPR/Cas12a, editing activity improvement, high throughput, engineered variant

## Abstract

CRISPR/Cas12a (formerly Cpf1), an RNA-guided endonuclease of the Class II Type V-A CRISPR system, provides a promising tool for genome engineering. Over 10 Cas12a orthologues have been identified and employed for gene editing in human cells. However, the functional diversity among emerging Cas12a orthologues remains poorly explored. Here, we report a high-throughput comparative profiling of editing activities across 16 Cas12a orthologues in human cells by constructing genome-integrated, self-cleaving, paired crRNA–target libraries containing >40,000 guide RNAs. Three Cas12a candidates exhibited promising potential owing to their compact structures and editing efficiency comparable with those of AsCas12a and LbCas12a, which are well characterized. We generated three arginine substitution variants (3Rv) via structure-guided protein engineering: BsCas12a-3Rv (K155R/N512R/K518R), PrCas12a-3Rv (E162R/N519R/K525R), and Mb3Cas12a-3Rv (D180R/N581R/K587R). All three Cas12a variants showed enhanced editing activities and expanded targeting ranges (NTTV, NTCV, and TRTV) compared with the wild-type Cas12a effectors. The base preference analysis among the three Cas12a variants revealed that PrCas12a-3Rv shows the highest activity at target sites with canonical PAM TTTV and non-canonical PAM TTCV, while Mb3Cas12a-3Rv exhibits recognition features distinct from the others by accommodating for more nucleotide A at position −3 for PAM TATV and at position −4 for PAM ATCV. Thus, the expanded Cas12a toolbox and an improved understanding of Cas12a activities should facilitate their use in genome engineering.

## 1. Introduction

CRISPR/Cas systems are adaptive immune systems found in bacteria and archaea that defend prokaryotes against bacteriophages and other mobile genetic elements deemed to be revolutionary tools for genetic engineering. Type II Cas9 and Type V-A Cas12a, two class II CRISPR/Cas systems utilizing single-protein effectors combined with guide RNAs to cleave complementary DNA targets, are widely used for gene, epigenetic, and base editing in human cells and other organisms [1,2,3,4].

Compared with Cas9, endonucleases from the Cas12a family exhibit various distinct features: (i) more compact structures; (ii) guidance by a single short crRNA without the need to transactivate CRISPR RNA; (iii) both DNase and RNase activities, and CRISPR array processing; (iv) recognition of a target with T-rich protospacer adjacent motifs (PAMs) located at the 5 end; (v) generation of PAM-distal staggered overhangs; and (vi) relatively low off-target effects [5,6]. These properties make Cas12a effectors particularly promising when targeting AT-rich genomic regions applied for multiplexed gene editing and applications requiring high specificity [7,8,9,10,11]. Additionally, Cas12a collaterally cleaves non-specific single-strand DNA triggered by target binding and has been used to develop diverse virus-detection kits [12,13,14,15].

Since CRISPR/Cas12a was first developed in 2015, over 10 Cas12a orthologues have been identified and employed for gene editing in human cells [16,17,18,19,20,21,22,23,24,25]. However, only AsCas12a (*Acidaminococcus* sp. BV3L6) and LbCas12a (*Lachnospiraceae bacterium* ND2006) have been well characterized, engineered, and widely used for genome targeting in eukaryotic cells [17,26,27,28,29], while other Cas12a orthologues were only identified for editing activities by targeting a small number of commonly used endogenous target sites. A lack of systematic, extensive, and comparative evaluations resulted in insufficient understanding of the range of properties and activities of these Cas12a. Moreover, the moderate editing activity and stringent targeting range of wild-type Cas12a orthologues also limit further applications that require efficient gene editing. Hence, screening for potential Cas12a candidates and engineering them for improved editing efficiency and broader PAM compatibility are needed.

Given the potential advantages of the Cas12a system, we sought to improve their overall editing activity by identifying novel Cas12a effectors and by generating engineered variants. Here, we selected 16 Cas12a orthologues already identified as possessing editing activities in human cells and performed an extensive and comparative profiling of their Cas12a editing activities using a modified high-throughput strategy based on genome-integrated, self-cleaving, paired guide-RNA–target libraries. Three Cas12a candidates (PrCas12a, Mb3Cas12a, and BsCas12a) showed potential, exhibiting editing activities comparable with those of AsCas12a and LbCas12a when targeting sites with canonical TTTV PAMs (V = A/C/G) and with more compact structures in favor of protein delivery. We then engineered these three Cas12a orthologues with a combination of three arginine substitutions to generate variants with improved editing activity and expanded the PAM recognition range. Finally, by generating novel engineered Cas12a variants, we aimed to expand the CRISPR/Cas12a toolbox and to improve their targeting capabilities and flexibility, which would facilitate further Cas12a-based applications.

## 2. Results

### 2.1. Modified Strategy for High-Throughput Profiling of Cas12a Editing Activities

To characterize the editing activities of multiple Cas12a orthologues and variants, we adopted and modified the high-throughput evaluation methods applied to Cas9 and Cas12a systems from previous studies [30,31,32]. For target sites with different PAM compositions, three lentiviral plasmid libraries (Lib-A, B, and C) containing 12,544, 12,543, and 12,363 pairs of guides and corresponding target sequences, respectively, were array-synthesized and prepared from oligonucleotide pools. Lib-A contained targets with canonical TTTV PAMs, while Lib-B and Lib-C included targets with altered PAMs (see Section 4). In addition, eight sub-libraries were constructed based on Lib-A, B, and C using amplifying primers including base substitutions in the loop region of crRNA scaffolds. Sub-libraries with crRNA loop region variants were generated and combined with compatible Cas12a effectors in cleavage assays (Supplementary Table S3).

**Figure 1:**
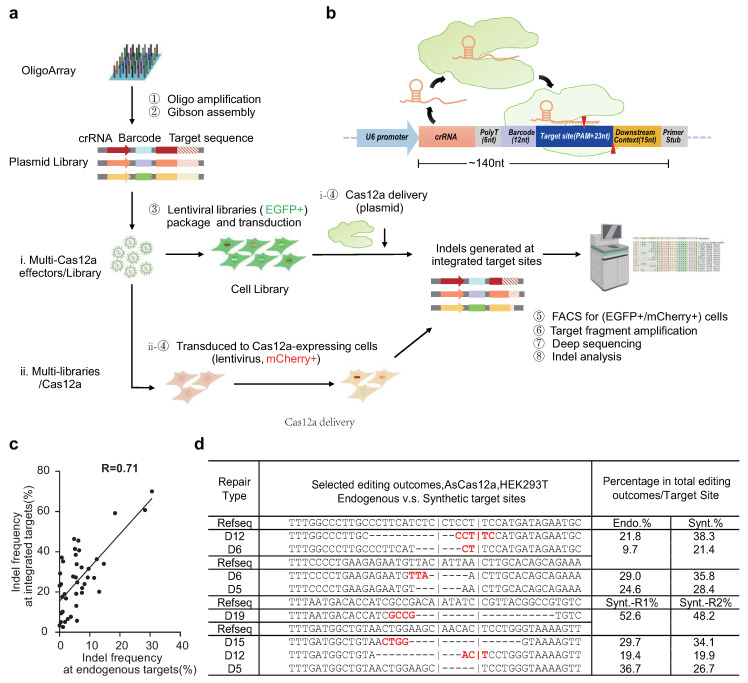
Paired guide-RNA–target library preparation and high-throughput profiling of editing activity mediated by diverse Cas12a effectors. The editing efficiency of Cas12a at synthetic target sites is reproducible and closely resembles endogenous editing profiles. (**a**) Schematic representation of a workflow for high-throughput assaying of Cas12a-mediated editing efficiency in human cells. (**b**) Design of oligonucleotides containing a self-cleavage crRNA–target paired library. (**c**) Correlation of indel frequencies measured at integrated target sites introduced via lentiviruses and corresponding endogenous sites edited by AsCas12a in HEK293T (*n* = 42, Pearson (r)). (**d**) Sequence alignment examples showing the reproducibility of repair outcomes and the proportional editing efficiency generated by AsCas12a between synthetic (Synt.) and endogenous (Endo.) target sites. For details, see the Supplementary Note.

The paired guide–target structure provided a highly efficient self-cleavage system by ensuring that the guide RNA and its corresponding target site were in the same cell and easy to recover and identify [32]. After this integration into the genomes of the target cell line by lentivirus transduction, the transcription of guide RNAs (crRNAs) was initiated by the upstream U6 promoter and terminated by poly T. Then, the guide RNA was combined with the Cas12a effectors, which led to editing and indel generation at integrated target site sequences located downstream (Figure 1a,b).

Since up to 20 Cas12a effectors (16 wild-type Cas12a orthologues and 4 Cas12a variants) are involved in our work (Supplementary Table S2), to simplify the protocol and to increase the reliability of this extensive evaluation, we adapted methods for assessing multiple Cas12a effectors targeting multiple paired-guide–target libraries by creating two cleavage assay strategies: (i) When multiple Cas12a effectors target a certain guide–target library, the paired guide–target pool-integrated cell libraries were constructed first by lentivirus infection, followed by diverse Cas12a effector delivery through plasmid transfection. (ii) When one Cas12a effector targets multiple guide–target libraries with different crRNA scaffolds or PAM groups, the target cell lines stably expressing certain Cas12a effectors were established first by lentivirus infection, followed by the transduction of diverse lentiviruses containing various guide–target libraries. FACS was performed to sort through the cell population for dual-fluorescence positivity (EGFP+/mCherry+), which refers to cells containing both Cas12a effectors and integrated guide–target libraries. The collected cells were cultured until 7 days post-transduction/transfection. Then, genomic DNA were extracted, and full-length integrated guide–target fragments were amplified and analyzed using deep sequencing to evaluate the editing outcomes and indel frequencies (Figure 1b, Supplementary Figure S2).

The appropriate outgrowth time for measuring Cas12a editing efficiency was determined to be seven days post-editing based on the timeline of a cleavage assay built with multiple collection time points of the cell population during the cleavage assay (3 d, 5 d, 7 d, and 10 d post-editing). After 7 days of outgrowth from the lentiviral library infection, the editing rates of the target sites became nearly saturated and the repair outcome profiles tended to be stable (Supplementary Figure S4) [33]. After the cleavage assays, the deep-sequencing data showed that, among the 12,544 designed pairs, 12,037 (96.0%) (i) and 12,114 (96.6%) (ii) pairs were included in the cells, suggesting a relatively high quality of the guide–target library construction with integrity and uniformity (Supplementary Figure S1 and Table S1).

Next, to assess the reproducibility of the cleavage assay, we evaluated the correlations of indel frequencies measured at the same integrated target site between two independent biological replicates. By analyzing the data collected from four Cas12a effectors editing over 700 targets in Lib-A in three cell lines (HEK293T, RKO, and Hela), we observed a relatively strong correlation between the indel frequencies measured at the synthesized target sites from two replicates (Supplementary Figure S3; Pearson’s correlation coefficient r ranged from 0.55 to 0.73), indicating that the editing activities of various Cas12a orthologues and variants measured at these integrated target sites in a high-throughput manner are reproducible in diverse cell lines (Supplementary Figure S3).

Furthermore, to assess the reliability of the cleavage assay, next, we evaluated the correlations of the indel frequencies measured at integrated sequences and their corresponding endogenous sites, determined using individual tests. We selected 42 targets from Lib-A and observed a strong correlation between the indel frequencies measured at endogenous sites and their corresponding integrated target sequences (Figure 1c, Pearson’s correlation coefficient r = 0.71). However, the indel frequencies measured at endogenous target sites were overall lower compared with those of the corresponding integrated target sites. The lower activities at endogenous targets can be attributed to the highly variable chromatin accessibility, while lentivirally integrated fragments tended to be inserted into local hotspots, which were usually more active in gene expression and more accessible for Cas12a effectors to edit [34,35].

In addition, we selected specific repair outcome types and measured their frequencies in certain edited target sites to evaluate the reproducibility of the method in detail (Figure 1d). We compared the frequencies of specific repair types generated by the same guide RNA between two independent synthetic replicates (Synt.R1 v.s. Synt.R2) or between integrated sites and their corresponding endogenous target sites (Synt. v.s.Endo.) edited by AsCas12a in HEK293T cells. For example, at target site 4, a specific repair type with a 15-bp deletion (D15) made up 29.7% and 34.1% of the edited products generated by the guide in Synt.R1 and Synt.R2, respectively, while at target site 1, a specific repair type with a 12-bp deletion (D12) made up 21.8% and 38.3% of the edited products generated by the guide in Endo. and Synt., respectively. Moreover, without a DNA template, double-strand breaks (DSB) generated by Cas12a are mainly repaired through two repair pathways: NHEJ (non-homologous end joining) and MMEJ (microhomology-mediated end joining) (Supplementary Figure S5). During the analysis, we observed that the editing outcomes of Cas12a mediated by the MMEJ repair pathway were more reproducible than those mediated by NHEJ repair pathway because MMEJ-mediated repair is more predictable according to the microhomology units around the expected cleavage sites of Cas12a (Figure 1d).

In summary, the Cas12a editing efficiencies measured at the genome-integrated synthetic target sites through our modified evaluation method were demonstrated to be reproducible and reliable and to closely resemble endogenous editing profiles.

### 2.2. Comparative and Extensive Analysis of Editing Activities across 16 Cas12a Orthologues in Human Cells Revealed Three Potential Cas12a Candidates

**Figure 2:**
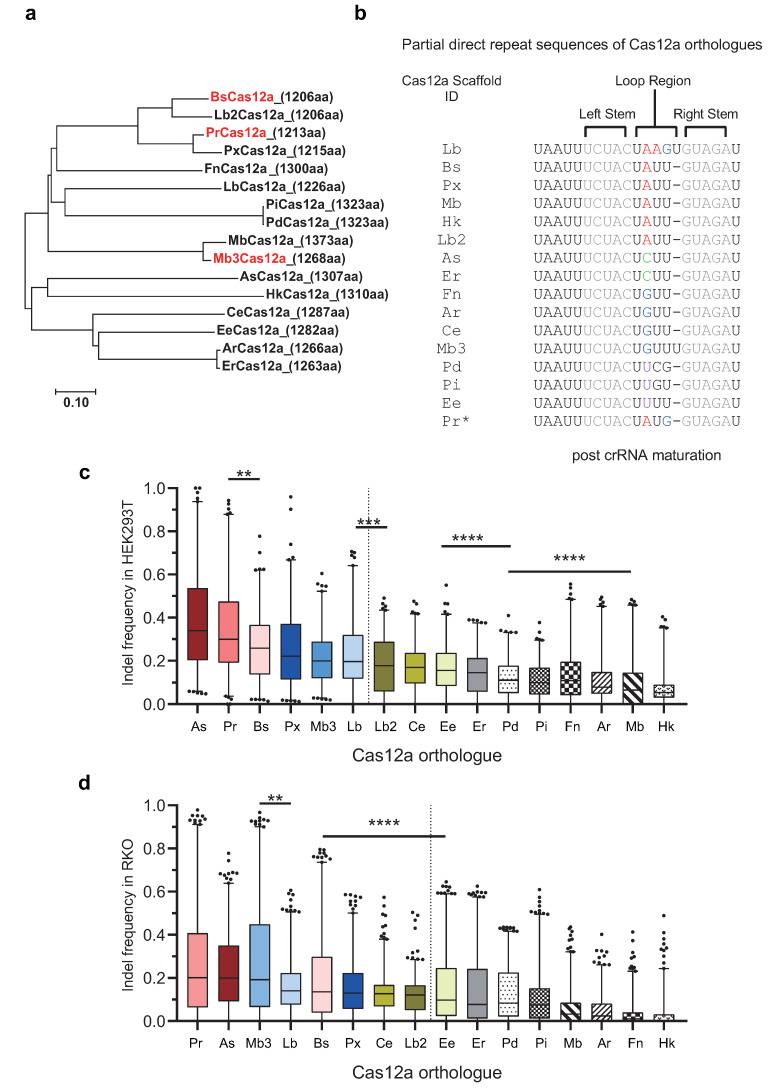
High-throughput evaluation of the editing activity of 16 Cas12a orthologues on target sites with canonical TTTV PAMs in HEK293T and RKO cells. (**a**) Phylogenetic tree of 16 Cas12a orthologues generated by MEGA7.0.26 (see Supplementary Figure S6). (**b**) Alignment of direct repeats (DRs) from the 16 Cas12a orthologues. The conserved stem duplex is highlighted in gray, and the non-conserved bases in the loop region are colored. (**c**,**d**) Editing activity evaluation among 10 Cas12a orthologues at target sites with TTTV PAMs in HEK293T (c, *n* = 500) and RKO (d, *n* = 1000) cells. V = A/C/G. Box-whisker plots: median line, quartiles for box edges, 1–99% whiskers. ** *p* < 0.01, *** *p* < 0.001, **** *p* < 0.0001. For details of sample size and of the statistical analyses, see the Supplementary Note.

To comparatively evaluate the editing activities among Cas12a effectors in human cells, we first selected 16 wild-type Cas12a orthologues, which were identified and demonstrated to have editing activities in human cells in previous studies [16,17,18,19,20,21,22,23,24,25]. Phylogenetic trees were constructed based on amino acid sequence similarity in the coding region of the Cas12a orthologues, with the percent identity ranging from 29.3% (between HkCas12a and PdCas12a) to 99.6% (between PiCas12a and PdCas12a) and a coding region length ranging from 1206 to 1373 amino acids (Figure 2a, Supplementary Figure S6).

Generally, when a novel wild-type Cas12a orthologue was identified, a corresponding crRNA scaffold-encoding sequence could be found from the direct repeat (DR) region in the genome of originated strains. The transcription of crRNA consists of a 5’ end handle of the scaffold (19–20 nt) forming a stem-loop structure, followed by a specific spacer (18–24 nt) for targeting. The wild-type crRNA scaffolds of diverse Cas12a orthologues are highly conserved, only with base substitutions leading to variants in the loop region (Figure 2b). Most Cas12a orthologues showed tolerance to the loop region variants, and the editing activity is not notably affected when Cas12a was combined with other crRNA scaffolds instead of the wild-type, such as AsCas12a, BsCas12a, PrCas12a, and Mb3Cas12a [18,20,36], while some Cas12a orthologues were stringently conserved on the loop region of the crRNA scaffold. Even a single-base substitution causes drastic impairment on the editing activity of Cas12a, such as LbCas12a [36]. Hence, we generated guide–target libraries with diverse loop region variants in the crRNA scaffold according to the wild-type DRs for combination with the corresponding Cas12a orthologues. Moreover, for a Cas12a less sensitive to loop region variants, we also attempted combinations with multiple crRNA scaffolds for further improvement in their editing activity (Figure 2b, Supplementary Table S5).

For an extensive comparison, we performed high-throughput evaluations of the editing activities of 16 Cas12a orthologues targeting 500 target sites with canonical TTTV PAMs (from Lib-A, V = A/C/G) in HEK293T cells. To increase the accuracy and reliability of evaluation, only guide–target pairs with read counts over 300 and present in the sequencing results of all 16 Cas12a were selected for the dataset. The 16 Cas12a were ranked by level of editing performance from high to low and divided into two categories. Category 1 included six Cas12a (As-, Pr-, Bs-, Px-, Mb3-, and LbCas12a) with relatively high editing efficiencies and average indel frequencies ranging from 22.7% to 39.0%. In the high-performance category, AsCas12a (39.0%) and PrCas12a (34.3%) exhibited notably higher editing efficiencies compared with the other four Cas12a. Category 2 included the other 10 Cas12a with relatively lower editing efficiencies and average indel frequencies of less than 20% (7.2–17.9%) (Figure 2c).

Furthermore, to validate the results of the editing activity comparison, we repeated the high-throughput evaluations of the 16 Cas12a on 1000 target sites with the canonical TTTV PAMs in RKO cells. The high-activity category contained eight Cas12a (Pr-, As-, Mb3-, Lb-, Bs-, Px, Ce-, and Lb2Cas12a) and average indel frequencies ranging from 12.3% to 26.2%, in which PrCas12a (26.2%), AsCas12a (23.0%), and Mb3Cas12a (27.4%) performed better than the other five Cas12a. The low-activity category included the remaining eight Cas12a and a median indel frequency of less than 10% (Figure 2d). Compared with HEK293T, the overall reduction in the editing activities in RKO may be attributed to the influence of the cell’s genetic background (Supplementary Figure S7) [37,38]. Therefore, although with slight fluctuations in the ranking, we observed that the overall trend in editing activities was similar to that in HEK293T cells, suggesting the reliability and universality of the evaluation results.

For the high-activity group, besides the well-characterized and widely used AsCas12a and LbCas12a, we found that three other Cas12a (PrCas12a, Mb3Cas12a, and BsCas12a) were consistently present, showing comparable editing activities on target sites with T-rich PAMs. In addition, these three Cas12a showed more compact structures (1206–1268 aa) compared with AsCas12a (1307 aa), which facilitates Cas12a delivery by reducing the difficulty of packaging full-length Cas12a encoding sequences (Figure 2a,c,d).

Mb- and FnCas12a, from the low-activity group, were previously identified at the same time as As- and LbCas12a, but their relatively low editing activities in human cells limited their application in various organisms. In previous activity studies, when targeting several common endogenous target sites for gene editing tests, such as from the gene loci of *DNMT1*, *EMX1*, and *VEGFA*, Mb- and FnCas12a showed relatively lower but moderate editing activities [39]. However, in our high-throughput evaluation, the editing activities of these two Cas12a were too low to be employed for efficient gene editing, suggesting an overestimation of their overall editing efficiency when evaluated using only a small number of well-known target sites. For other Cas12a orthologues such as HkCas12a and PiCas12a, although these Cas12a were determined to recognize T-rich PAMs, TTTV was not their optimal PAM anymore. Both showed extremely low activity on TTTV PAMs. As HkCas12a and PiCas12a were characterized as recognizing YYN and KKYV PAMs (N = A,C,G,T; K = G,T), respectively, it seems that HkCas12a preferred C-rich and PiCas12a preferred G-rich PAMs more than the canonical TTTV PAMs [20,22] (Figure 2c,d).

Together, we identified three potential Cas12a candidates (PrCas12a, Mb3Cas12a, and BsCas12a) with more compact structures and editing activities comparable with those of the well-characterized AsCas12a and LbCas12a after an extensive comparison of 16 Cas12a orthologues. These three Cas12a candidates were subjected to further engineering and characterization.

### 2.3. Engineering Pr-, Mb3-, and BsCas12a with Combination of Arginine Substitutions

**Figure 3:**
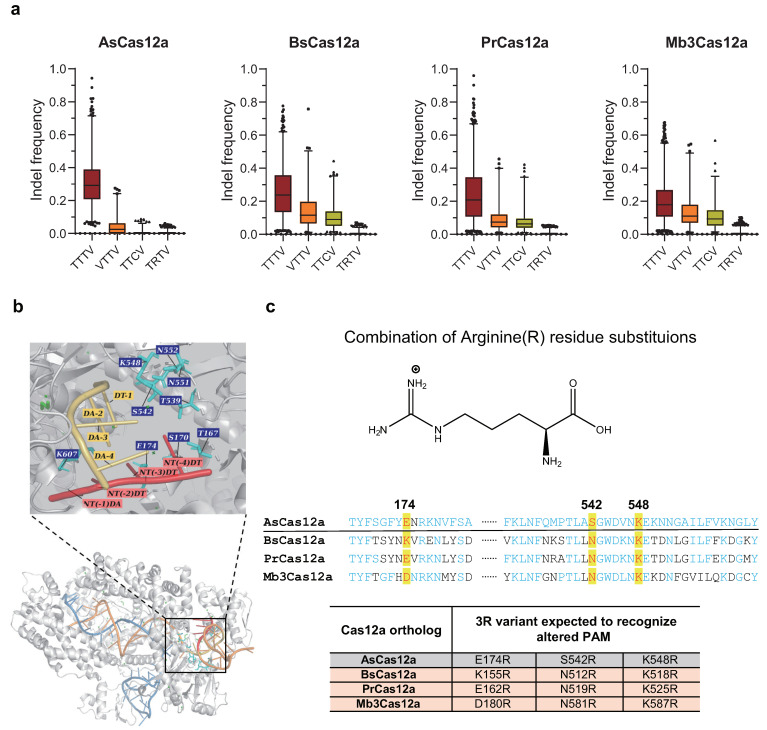
Engineered Cas12a variants with three arginine substitutions for enhanced editing activity and expanded PAM compatibility. (**a**) Editing activity assessment of four wild-type Cas12a (AsCas12a, BsCas12a, PrCas12a, and Mb3Cas12a) on targets with canonical and non-canonical PAMs. Box-whisker plots: median line, quartiles for box edges, 1–99% whiskers. (**b**,**c**) Structure-guided mutation design of three Cas12a variants for a wider PAM recognition range. (**b**) Structural representation of the AsCas12a (gray)–crRNA(orange)–target dsDNA(blue) ternary complex. The structural image was generated from PDBID:5B43 and visualized in PyMOL(version 2.1.0) [40] (Adapted with permission, Copyright © 2016, Elsevier). (**c**) Positive arginine residue and 3R variant design of the four Cas12a orthologues for an expanded target range. (**top**) Amino acid sequence alignment of the four Cas12a orthologues flanking the three PAM-proximal residues (E174, S542, and K548 in AsCas12a) verified as contributing to improved editing activity and a wider recognition range of AsCas12a, named enAsCas12a-HF (E174R/N282A/S542R/K548R) [27]. The yellow highlights with red letters represent mutation sites, blue letters represent a match with AsCas12a, and the black letters represent a mismatch with AsCas12a. The position numbers on top refer to the amino acid sequences of AsCas12a. (**bottom**) Three engineered Cas12a variants for enhanced editing efficiency and expanded target range. For more details, see the Supplementary Note.

PAM determination assays showed a relatively stringent recognition range for the four wild-type Cas12a effectors (AsCas12a, BsCas12a, PrCas12a, and Mb3Cas12a) (Figure 3a). All four Cas12a orthologues exhibited the most robust activity on targets with canonical TTTV PAMs. In contrast with the well-characterized AsCas12a, the three novel Cas12a candidates showed basic editing activity similar to that of the wild-type Cas12a for targets with non-canonical PAMs (VTTV and TTCV), while all four Cas12a exhibited nearly zero activity on targets with TRTV PAMs, indicating low tolerance to A or G substitution at position −3 of the PAM.

We used structure-guided protein engineering to enhance the editing activity and to expand the PAM recognition range of the three Cas12a candidates that we identified. A structural representation of the AsCas12a–crRNA–target dsDNA ternary complex (Cas12a paired with a crRNA and interacting with a target site encoding a canonical TTTA PAM) was used to determine the key amino acid positions that might contribute to target binding, PAM recognition, and DNA cleavage [40]. Nine amino acid positions adjacent to or with potential to interact with PAM sequences were identified: T167, S170, E174, T539, S542, K548, N551, N552, and K607 (Figure 3b). Nine AsCas12a variants bearing single amino acid substitutions of arginine at the corresponding positions were tested for activities on target sites with canonical TTTV PAMs. Three of them (E174R, S542R, and K548R) displayed higher editing activities on target sites with canonical PAMs relative to the wild-type AsCas12a. An additional AsCas12a variant harboring the combination of these three arginine substitutions exhibited robust editing activities on canonical sites while acquiring a wider target range by recognizing non-canonical PAMs such as VTTV, TTCV, and TRTV (V = A/C/G) [27].

Arginine was selected and used for amino acid substitutions when generating more robust Cas12a for several reasons. First, arginine residues possess a characteristic side chain containing guanidinium, which provides five potential hydrogen bond donors that interact with other polar groups. Second, arginine residues are the most common residues involved in the formation of protein/DNA complexes because they are positively charged at physiological pH, which means that they are more accessible for binding to molecules with negative charges, such as nucleic acids. Moreover, the relatively longer side chain of arginine residues improved the flexibility of the amino acid to interact with other molecules [41]. In addition, the introduction of arginine substitutions to alter PAM recognition has already been used in several studies of Cas12a and Cas9 engineering [42,43].

Hence, we introduced a combination of three arginine substitutions into the corresponding positions based on the amino acid sequence alignment of three Cas12a candidates with AsCas12a (Figure 3c). Three Cas12a variants containing three arginine substitutions (3Rv) were generated: BsCas12a-3Rv (K155R/N512R/K518R), PrCas12a-3Rv (E162R/N519R/K525R), and Mb3Cas12a-3Rv (D180R/N581R/K587R) (Figure 3c).

### 2.4. Characterization of Pr-, Mb3-, and BsCas12a Variants with Enhanced Editing Activity and Expanded Target Range

**Figure 4:**
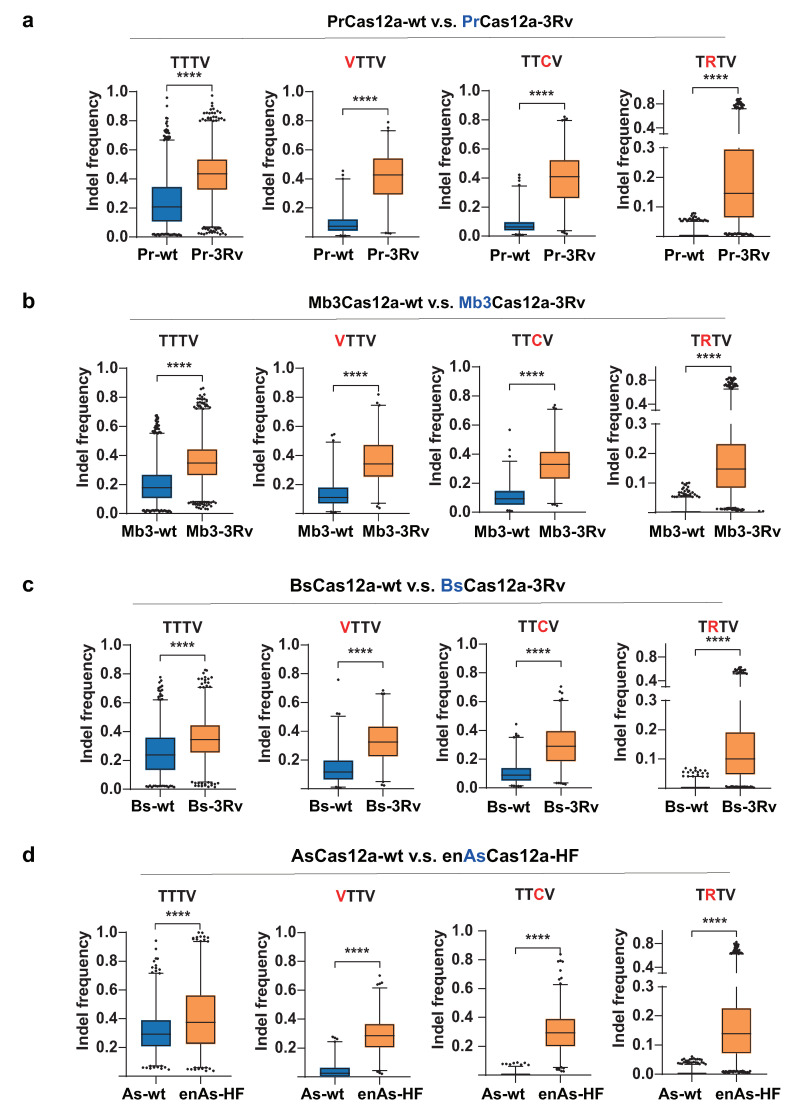
Editing activity comparison between the wild-type (WT) and variants with three arginine substitutions (3Rv) of two Cas12a orthologues (PrCas12a (**a**), Mb3Cas12a (**b**), BsCas12a (**c**), and AsCas12a (**d**)) at targets with canonical PAM sequences (TTTV) and non-canonical PAM sequences (VTTV, TTCV, and TRTV) in HEK293T cells. V = A/C/G; R = A/G. The same set of target sites was chosen to evaluate the editing efficiency between WT Cas12a and engineered Cas12a-3Rv mutants. Box-whisker plots: median line, quartiles for box edges, 1–99% whiskers. **** *p* < 0.0001. For details of sample size and of the statistical analyses, see the Supplementary Note.

To examine the editing activities and targeting range of the engineered Cas12a variants, we performed high-throughput profiling of the editing efficiencies generated by four Cas12a variants from the study by Kleinstiver [27]: PrCas12a-3Rv, Mb3Cas12a-3Rv, BsCas12a-3Rv, and enAsCas12a-HF. The target sites with four groups of PAMs were measured, including the canonical TTTV PAMs and non-canonical PAMs (VTTV, TTCV, and TRTV; V= A/C/G; R = A/G) expected to be edited with the altered PAM recognition of the Cas12a variants. The same set of target sites containing certain PAM types was chosen to evaluate the editing efficiency between wild-type Cas12a and engineered Cas12a mutants. The AsCas12a versus enAsCas12a-HF group was used as the control and tested for the reliability of our editing activity evaluation method (Figure 4d). The engineered Cas12a variants were first applied to target several endogenous target sites to demonstrate their editing activities (Supplementary Figure S8) and then subjected to integrated lentivirus libraries for extensive activity evaluation.

All three Cas12a variants generated in this work showed improved editing activity and expanded PAM recognition range compared with their wild-type Cas12a.

For target sites with canonical TTTV PAMs, all three Cas12a variants displayed higher editing activities. The average indel frequencies against TTTV PAMs increased from 24% (PrCas12a), 20% (Mb3Cas12a), and 26% (BsCas12a) to 43% (PrCas12a-3Rv), 36% (Mb3Cas12a-3Rv), and 35% (BsCas12a-3Rv) (Figure 4).

For target sites with non-canonical VTTV and TTCV PAMs, in contrast with AsCas12a, the three Cas12a candidates showed basic editing activities similar to that of the wild-type Cas12a. After the three arginine substitutions, all three Cas12a variants exhibited a further enhancement in editing activity on these altered PAMs. The average indel frequencies against VTTV PAMs increased from 9% (PrCas12a), 16% (Mb3Cas12a), and 15% (BsCas12a) to 41% (PrCas12a-3Rv), 36% (Mb3Cas12a-3Rv), and 33% (BsCas12a-3Rv). The average indel frequencies against TTCV PAMs increased from 8% (PrCas12a), 11% (Mb3Cas12a), and 10% (BsCas12a) to 39% (PrCas12a-3Rv), 33% (Mb3Cas12a-3Rv), and 29% (BsCas12a-3Rv) (Figure 4a–c).

For target sites with non-canonical TRTV PAMs, three wild-type Cas12a candidates showed almost zero activity, consistent with AsCas12a, while after engineering, all three Cas12a variants exhibited moderate improvement in their editing efficiencies. The average indel frequencies against TRTV PAMs increased to 20% (PrCas12a-3Rv), 18% (Mb3Cas12a-3Rv), and 14% (BsCas12a-3Rv) (Figure 4a–c).

Taken together, the three engineered Cas12a variants (PrCas12a-3Rv, Mb3Cas12a-3Rv, and BsCas12a-3Rv) exhibited improving editing activity on target sites with canonical TTTV PAMs and expanded targeting ranges to recognize non-canonical PAMs (VTTV, TTCV, and TRTV). Among the four Cas12a variants tested, PrCas12a-3Rv showed the most robust editing activity against both canonical and altered PAMs, followed by Mb3Cas12a-3Rv and then BsCas12a-3Rv.

### 2.5. Base Preference Determination in PAM Recognition among Four Cas12a Variants

**Figure 5:**
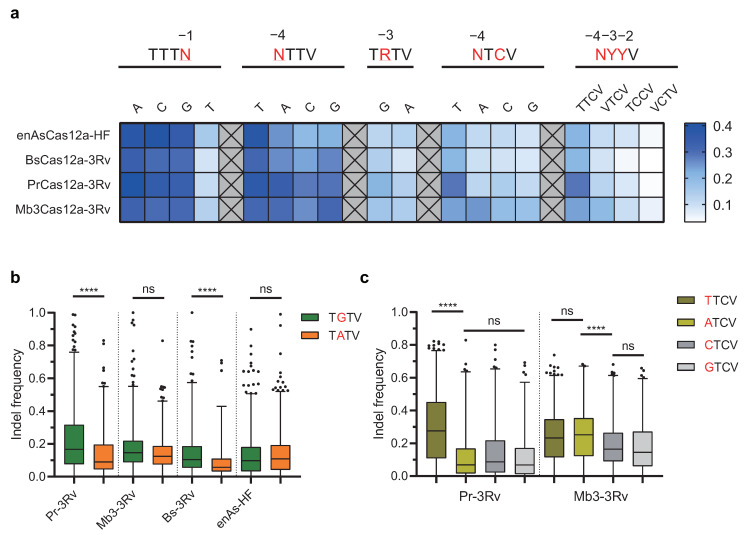
Characterization of base preferences in PAM recognition across four engineered Cas12a variants. (**a**) Base preference characterization in the 4-nt PAM sequences of four Cas12a variants (enAsCas12a-HF, BsCas12a-3Rv, PrCas12a-3Rv, and Mb3Cas12a-3Rv). The values in the heatmap show a comparison of the average indel frequencies induced by the four Cas12a variants at target sites with different PAM sequences. The white-to-dark-blue gradient represents increasing editing efficiency. Degenerate bases are highlighted in red with a number marked on top to indicate its positions in the PAM tested for base preference. For exact values of the average editing efficiencies, see the Supplementary Note. (**b**) Comparison of tolerance for nucleotides G and A at position −3 in PAM TRTV (R = A/G; V = A/C/G) among the four Cas12a variants. (**c**) Base preference at position −4 in the PAM NTCV (N = A/C/G/T) of PrCas12a-3Rv and Mb3Cas12a. (**b**,**c**) Box-whisker plots: median line, quartiles for box edges, 1–99% whiskers. **** *p* < 0.0001; ns: not statistically significant. For details of sample size and of the statistical analyses, see the Supplementary Note.

To comprehensively profile the PAM requirements of the three newly engineered Cas12a variants, we analyzed their base preferences in canonical and altered PAMs in comparison with the AsCas12a variant (enAsCas12a-HF). First, we characterized their base preferences in five degenerate PAM groups. Including position −1 in TTTN PAMs, position −4 in NTTV PAMs, position −3 in TRTV PAMs, position −4 in NTCV PAMs, and position −4 to −2 in C-rich NYYV PAMs. The average editing frequencies of the targets with a specific PAM sequence measured in the high-throughput evaluation were categorized and shown in a heatmap (Figure 5a, Supplementary Figure S9).

Since in their engineering, PrCas12a, Mb3Cas12a, and BsCas12a shared the same combination of substitutions transferred from the corresponding amino acid positions in AsCas12a, theoretically, they should also exhibit similar altered PAM recognition patterns, such as the RVR mutant of AsCas12a transferred into LbCas12a [28]. However, we observed notable differences in the base preferences of several PAMs between these Cas12a variants in the comparison.

At position −3 in the target sites with TRTV PAMs (R = A/G), PrCas12a-3Rv and BsCas12a-3Rv notably preferred nucleotide G over A, while Mb3Cas12a-3Rv exhibited less bias for nucleotide A and G, which was similar to enAsCas12a-HF (Figure 5b). PrCas12a-3Rv exhibited average indel frequencies of 21% against TGTV PAMs and 14% against TATV PAMs. BsCas12a-3Rv showed average indel frequencies of 14% against TGTV PAMs and 8% against TATV PAMs. In contrast, Mb3Cas12a-3Rv displayed comparable editing activities between TGTV and TATV PAMs, with average indel frequencies of 17% (TGTV) and 14% (TATV).

At position −4 in target sites with NTCV PAMs (N = A/C/G/T), PrCas12a-3Rv, BsCas12a-3Rv, and enAsCas12a-HF strongly favored nucleotide T over V (V = A/C/G). Take PrCas12a-3Rv for example; the average indel frequency against TTCV PAMs was 29% but was reduced by half when targeting ATCV (11%), CTCV (14%), and GTCV (11%) PAMs, while Mb3Cas12a-3Rv behaved differently and preferred nucleotide A over T, with average indel frequencies of 24% (TTCV), 25% (ATCV), 19% (CTCV), and 18% (GTCV) (Figure 5c). Of note, the base preference for nucleotide A at position −4 seemed restricted in NTCV PAMs because Mb3Cas12a-3Rv showed no sign of bias to A when targeting NTTV PAMs (Figure 5a).

Altogether, through the extensive base preference analysis, we found that Cas12a variants with the same mutant combination exhibited different recognition features. Among the four Cas12a variants examined, PrCas12a-3Rv displayed the most robust editing activities against canonical TTTV PAMs and non-canonical TTCV PAMs, while Mb3Cas12a-3Rv recognized the broadest targeting range by accommodating for more nucleotide A at position −3 for PAM TATV and at position −4 for PAM ATCV. Collectively, Mb3Cas12a-3Rv could target many PAMs including NTTV (TTTV/ATTV/CTTV/GTTV), NTCV (TTCV/ATCV/CTCV/GTCV), TRTV (TATV/TGTV), and others.

## 3. Discussion

CRISPR/Cas12a effectors are promising alternatives to the widely used Cas9 nucleases in multiple gene-editing-based applications. To provide more available options for Cas12a effectors in addition to the most employed As- and LbCas12a, here, we expanded the Cas12a toolkit by introducing three new Cas12a variants engineered from potential candidates in the high-throughput activity profiling of 16 Cas12a orthologues. Three Cas12a variants were generated through three arginine substitutions via structure-based protein engineering for improved editing activity and expanded targeting ranges, including PrCas12a-3Rv (E162R/N519R/K525R), Mb3Cas12a-3Rv (D180R/N581R/K587R), and BsCas12a-3Rv (K155R/N512R/K518R). An extensive base preference characterization revealed that PrCas12a-3Rv displayed the most robust editing activities against canonical TTTV PAMs and non-canonical TTCV PAMs, while Mb3Cas12a-3Rv recognized the broadest targeting range of PAMs including NTTV (TTTV/ATTV/CTTV/GTTV), NTCV (TTCV/ ATCV/CTCV/GTCV), TRTV (TATV/TGTV), and others by distinctively favoring nucleotide A at position −3 for PAM TATV and at position −4 for PAM ATCV. Furthermore, we identified two categories of intra-crRNA base pairing formation patterns that were detrimental to editing activity and provided simple but efficient strategies to rescue these low activity forms of guide RNAs. Together, we enhanced the overall editing activity of the Cas12a system by combining optimized guide RNA designs and the engineered Cas12a effectors with improved targeting capabilities.

In addition to the potential Cas12a candidates, the comparison of editing activities among the 16 Cas12a orthologues also showed that some Cas12a effectors, such as FnCas12a, MbCas12a, HkCas12a, and PiCas12a, are insufficient (Figure 2c,d), though they were demonstrated to be at least moderately effective in some endogenous activity studies [22]. When the Cas12a orthologues were first identified from new strains, the indel frequency at several endogenous target sites measured by T7E1 assays was considered the gold standard for genome editing activity in human cells. These target sites are generally from common gene loci that are adopted by many editing activity studies, such as *DNMT1*, *EMX1*, *CFTR*, and *RNF2* [20]. However, most of the editing activities evaluated using this small, limited number of target sites seemed to be overestimated, meaning that they are acceptable for verifying targets but insufficient for other practical target sites. We considered it sensitive for a quality analysis of Cas12a editing activity but not accurate enough for a quantity evaluation. Hence, we suggest that the in vivo high-throughput evaluation system could be extended to future newly identified CRISPR/Cas effectors for a thorough and extensive characterization of editing activity.

Furthermore, the PAM determination analysis revealed that, although capable of recognizing both TTCV and TATV PAMs, all three newly developed Cas12a variants exhibited higher editing activities against TTCV compared with TATV PAMs (Figure 4). The most robust AsCas12a variants reported, enAsCas12a-HF (E174R/N282A/S542R/K548R), showed a similar targeting bias, but another LbCas12a variant, LbCas12a-RVRR (G532R/K538V/ Y542R/K595R), exhibited a contrasting recognition pattern by preferring TATV over TTCV PAMs [27,29]. To explain this bias, we compared the mutant combination designs among the five other Cas12a variants and identified a key amino acid position responsible for this recognition bias. K548 from AsCas12a, corresponding to K538 in LbCas12a and K525 in PrCas12a, could be an incompatible position when subjected to amino acid substitutions in engineering for altered PAMs. When the Lysine (K) was replaced with arginine (R), Cas12a favored nucleotide C at position −2 in PAMs (TTCV) but only moderately loosened its restriction on position −3 in PAMs (TRTV) [27], while Cas12a with a Valine (V) substitution on this position remarkably improved the recognition of nucleotide A at position −3 (TATV), at the cost of barely no activity on TTCV PAMs, as validated in the LbCas12a-RVRR and AsCas12a-RVR variants [28,29]. Therefore, although diverse, engineered Cas12a variants could cover a similar expanded targeting range, they still have varied recognition priorities due to different mutant designs. We recommend selecting the optimal Cas12a variants for targeting specific PAMs to acquire the most robust activity in future Cas12a applications.

Moreover, during the high-throughput editing activity evaluation of Cas12a combined with variant crRNA scaffolds, we found that undesired folding structures of full-length crRNA contributed to an impairment in Cas12a editing efficiency due to two categories of base pairing formation—severe intra-spacer base pairing and scaffold-spacer base pairing— causing disrupted crRNA scaffold, suggesting limitations and an urgent need for improvement and optimization of the currently available guide-RNA predictors. Future work might include extensive analyses to decipher and provide optimization strategies and to develop an advanced prediction model for Cas12a guide designs to further improve the editing activity of the Cas12a system.

Collectively, our findings expanded the CRISPR/Cas12a genome editing toolbox and augmented the overall editing activity of Cas12a by generating novel robust Cas12a variants. The improved targeting capabilities and flexibility should encourage a broader adoption of the Cas12a system and should further enhance Cas12a-based genome engineering and therapeutic applications in human.

## 4. Materials and Methods

### 4.1. Oligonucleotide Library Design

Three oligonucleotide pools including 12,544 (Lib-A), 12,543 (Lib-B), and 12,363 (Lib-C) pairs of guide and their corresponding target sequences were synthesized using an in-house CustomArray™ B3 synthesizer combined with 12k slides (GenScript*®*, Nanjing, China). Each oligonucleotide was designed with a total length of 142 nt and contained the following elements: a 19 nt sequence of the 5 end handle and a 23 nt spacer for crRNA encoding, a 6 nt poly T to end the transcription of crRNA, a 12 nt unique barcode inserted for identifying the individual paired guide–target after deep sequencing, 42 nt of the target sequence corresponding to the guide that started with the PAM, and the local downstream context sequence extracted from the human genome. For all three pools, each oligonucleotide has a 20 nt constant part at either end for forward and reverse primer binding in oligonucleotide amplification (Figure 1b). Diverse forward primers (×8) with base substitutions in the loop region of the crRNA handle were designed to generate libraries with different crRNA scaffolds compatible with various Cas12a orthologues and variants for cleavage assay. For details of the combination between Cas12a effectors and paired guide–target libraries, see Figure 2b and Supplementary Table S4.

For synthetic guide–target pairs in all three pools, we selected 1383 hepatoma and colorectal cancer-associated human genes that were designed from transcripts (including 5’UTR, CDS, and 3’UTR) derived from the Ensembl database (http://grch37.ensembl.org/index.html (accessed on 10 May 2019)).

All of the targets in Lib-A containing canonical TTTV PAM (V = A, C, G) were designed for a comparative analysis of the editing activities among Cas12a orthologues. Targets with TTTV PAM were designed using an online guide predictor of Cas12a (CRISPR RGEN Tools [44,45]: http://www.rgenome.net/cpf1-database/ (accessed on 15 May 2019)). We assessed the following parameters (# of mismatches (1, 0, and 0), GC content between 20 and 80%, and four repeated thymidines excluded) and extracted the top 10 guide candidates (23 nt spacer) of each gene to form a paired guide–target library with an average of nine guides per gene (Python script: Extract TOP10 guide candidates with TTTN PAMs from RGEN).

To evaluate the editing efficiencies of Cas12a orthologues or variants on alternative PAMs, we designed targets with non-canonical PAM sequences in Lib-B and Lib-C. Lib-B contained targets with mostly C-rich PAMs such as TTCV, VTCV, VCTV, VCCV, and VTTV, while Lib-C contained TRTN and NTTT PAMs (R = A/G; N = A/C/G/T). Since few current online tools are available for designing guides of Cas12a targeting non-canonical PAMs, we prepared in-house Python scripts (Python 3.6.5) modified from the online predictor to design the guides and targets with altered PAMs (Python script: Design targets with altered PAM). As a subset of the target sequences in Lib-B and Lib-C, we also extracted ~200 targets with diverse PAMs of human-genome origin from previous Cas12a-activity studies as positive controls. For details of the PAM composition of the targets in the three libraries, see Supplementary Table S3.

To compare the indel frequencies between endogenous and integrated target sites, we selected 42 targets from Lib-A. Oligonucleotides containing the corresponding guide-RNA-encoding sequences were synthesized individually by Tsingke Biotechnology (Beijing, China).

### 4.2. Plasmid Library Preparation

To construct the paired guide–target libraries, array-synthesized oligonucleotides were amplified by PCR with KAPA HIFI HotStart ReadyMix (KK2602, KAPA Biosystems, Woburn, MA, USA) using corresponding primers for specific libraries (Supplementary Table S4). The PCR cycle numbers should be less than 20 to avoid amplification bias caused by over-amplification in the library while acquiring sufficient products. The PCR products were column-purified with the QIAquick Gel Extraction Kit (28706, Qiagen, Hilden, Germany). Backbone vector (Lenti-pU6-crtslib-EGFP, Supplementary Note) was linearized by digestion with type II restriction enzymes SphI and AvrII and assembled with purified PCR products using an NEBuilder HiFi DNA Assembly Master Mix (E2621, New England Biolabs, Ipswich, MA, USA). After assembly, 5 μL of the reaction was transformed into 100 μL of Trans1-T1 chemically competent cells (CD501-01, TransGen Biotech, Beijing, China). The transformed cells added with 400 μL antibiotic-free LB medium were recovered in 37 ${}^{\circ }$C for 1 h and seeded into LB liquid medium supplemented with 100 μg/mL ampicillin in a volume ratio of 1:100 for 14–16 h (overnight).

The resulting number of colonies was demonstrated to yield 20~30× library coverage in the pre-experiment, in which the transformed products were seeded on LB agar plates for colony number quantification. Plasmid DNA was extracted from the collected bacteria after centrifugation with EndoFree Maxi Plasmid Kit V2 (4992438, TIANGEN, Beijing, China). The extracted plasmid libraries were stored at −20 ℃ for the following lentivirus package.

crRNA transcription was initiated by an upstream U6 promoter on the vector, and a second coding unit initiated by the CMV promoter was used to express a fluorescent protein (EGFP) for convenient cell sorting and transduction efficiency evaluation.

### 4.3. Vector Cloning for Cas12a Expression

The coding sequences of 16 Cas12a orthologues (As-, Lb-, Fn-, Mb-, Bs-, Hk-, Ar-, Pr-, Px-, Mb3-, Pd-, Pi-, Lb2-, Er-, Ee-, and CeCas12a) that were reported to have editing activity in human cells were collected from published studies and are provided in the Supplementary Note under their GenBank accession numbers. For details of Cas12a (for example, original reports, targeting ranges, and source bacteria strains), see Supplementary Table S2 and Figure S6). Coding region sequences of Cas12a were synthesized by BGI Geneland Scientific (Yixing, Jiangsu, China). When synthesizing gene fragments, universal adapters were added on either side of the coding region for amplification and provided homologous arms for cloning into the expressing vector. The N-terminal adapter contains a partial EF1α promoter, the kozak sequence, and SV40 NLS, while the C-terminal adapter includes a C-terminal NLS and 3 × HA sequence (Supplementary Note). For Cas12a variant generation, primers containing base substitution encoding the mutant amino acid were prepared to amplify the wild-type Cas12a template into several fragments and then assembled into their full length using the NEBuilder Assembly kit (Supplementary Table S4).

Synthesized gene fragments were amplified by universal forward and reverse primers containing homologous arms corresponding to the ends of two linearized vectors (lentivirus and transfection vectors) (Supplementary Table S4).

To construct the plasmid expressing Cas12a effectors for the transfection and lentivirus package, amplified Cas12a fragments were cloned into the SphI-AvrII linearized backbone vector Lenti-pEF1α-Cas12a-P2A-mCherry using the NEBuilder Assembly kit.

### 4.4. Cell Culture and Lentivirus Production

HEK293T, RKO, A375, Hela, and U2OS cells were obtained from ATCC. The cell lines were cultured in Dulbecco’s Modified Eagle Medium (DMEM) supplemented with 10% fetal bovine serum (FBS) (10099141, Gibco, Thermo Fisher Scientific, Waltham, MA, USA) at 37 ${}^{\circ }$C and 5% CO${}_{2}$.

For the lentivirus package, ~7.0 × 10^6^ HEK293T cells (ATCC) were seeded on a 100 mm dish and cultured in DMEM supplemented with 10% FBS. The cell confluency needed to reach 90–95% before transfection. The cells were then transfected with the plasmid mixture using Lipofectamine™ 3000 Transfection Reagent (L3000150, Thermo Fisher Scientific, USA). According to the manufacturer’s instructions fit for the 100 mm dishes, the transfer plasmid (Lenti-pU6-crtslib-EGFP or Lenti-pEF1α-Cas12a-P2A-mCherry), pVSV-G (envelop vector, Addgene #138479), and pCMV-dR8.91 (packaging vector) [46] were mixed in Opti-MEM (31985062, Thermo Fisher Scientific, Waltham, MA, USA) for a total of 9 μg at a weight ratio of 4:1:4.

After 6 h of transfection, the culture medium was changed for 12 ml of fresh medium of DMEM supplemented with 20% FBS. Three batches of the virus-containing supernatant were collected at 24 h, 48 h, and 72 h after transfection. All batches of virus-containing medium were combined for lentivirus concentration. The virus-containing supernatant was collected in 50 ml EP tubes and centrifuged at 2000 r.p.m. for 5 min at 4 ${}^{\circ }$C. The supernatants were then filtered through a Millex™-HP Sterile Filter Unit with a pore size of 0.45 μm (SLHP033RB, MilliporeSigma™, Burlington, MA, USA) to remove cell debris. For lentivirus concentration, 5 × PEG 8000 was added to the supernatants at a volume ratio of 1 to 4, mixed, and then stored at 4 ${}^{\circ }$C for at least 16 h. After centrifugation at 3500 r.p.m for 5 min at 4 ${}^{\circ }$C, the supernatants were discarded and the pellets were resuspended in DMEM in 1.5 ml EP tubes and stored at −80 ${}^{\circ }$C until use.

### 4.5. Cell Library Generation and Cas12a Delivery into Target Cell Lines

Cell library generation: First, we evaluated the package efficiency between different virus production batches and searched for the appropriate infection volume. A small aliquot in a gradient (0, 5, 10, 20, and 40 μL) from each batch of concentrated virus-containing medium was used to transduce HEK293T or other targeting cell lines. Since both Cas12a-expressing and guide–target library lentiviruses contained fluorescent proteins (mCherry or EGFP), we estimated the titer of lentiviruses by measuring the percentage of fluorescence-positive cells.

On the day prior to transduction, 2 × 10^5^ targeting cells were plated per well into a 24-well plate in DMEM supplemented with 10% FBS. Gradient volumes of concentrated lentivirus were transduced into cells in the presence of 1 μL 10mg/mL polybrene (hexadimethrine bromide, H8761, Solarbio Life Sciences, Beijng, China), mixed, and cultured at 37 ${}^{\circ }$C and 5% CO${}_{2}$. The cells were harvested 72 h after transduction and analyzed for percentage of fluorescence-positive cells using BD FACSVerse™ Flow Cytometer (651154, BD Biosciences, NJ, USA). The MOI (multiplicity of infection, pfu number/cell) was calculated using the following formula:

(1) MOI=−ln(P(0))

(2) P(1)=1−P(0)

where *P*(*1*) refers to the percentage of positively infected cells (EGFP+ or mCherry+). We planned to transduce the lentivirus transferring guide–target library into cells at an MOI of 0.5–0.7. According to the conversion formula, the appropriate percentage of transduction determined by FACS should range from 39 to 50%. A standard curve between the percentage of positive transduction and the aliquot size of the lentivirus infection for every batch was generated, and the lentivirus usage for one cleavage assay of Cas12a performed on 4 × 10^6^ cells in 100 mm dishes was calculated.

On the day prior to transduction, 1.5~2.0 × 10^6^ targeting cells were seeded on 100 mm dishes. The lentivirus was transduced into targeting cell lines in the presence of 8 μL of 10 mg/mL Polybrene. After 3 days of transduction, the cells were collected to sort for EGFP-positive cells using fluorescence-activated cell sorting (FACS). The sorted cells were cultured in DMEM supplemented with 20% FBS, CFX (Ciprofloxacin Hydrochloride, 10 μg/mL, C5075, USBiologica, Swampscott, MA, USA) and penicillin (100 U/mL)-streptomycin (100 mg/mL) (10378016, Gibco,Thermo Fisher Scientific, Waltham, MA, USA) at 37 ${}^{\circ }$C and 5% CO${}_{2}$. The cells were cultured until a total cell number of 2 × 10^7^, frozen, and stored in −80 ${}^{\circ }$C until use.

Cas12a delivery: when subjected to multiple Cas12a effectors targeting one guide–target library (Strategy-i, Figure 1a), Cas12a-expressing plasmids were transfected into the established cell library containing integrated guide–target pools using Lipofectamine™ 3000 Transfection Reagent; 4 μg of the Cas12a-expressing plasmids were transfected into 4 × 10^6^ cells in 100 mm dishes.

When subjected to the strategy of one Cas12a effector targeting multiple guide–target libraries with different crRNA scaffolds or PAM groups (Strategy-ii, Figure 1a), the lentivirus-expressing Cas12a were transduced into targeting cells, as previously described, to establish cell lines stably expressing the Cas12a effector. After cell sorting by FACS, the positive transduced cells were cultured until a total cell number of 2 × 10^7^, which was adequate for both storage and the following cleavage assay.

### 4.6. Cleavage Assay to Evaluate Editing Activity of Cas12a

Timeline cleavage assays were conducted to search for the optimal outgrowth time for cleavage assay. For all cleavage assays, at least two independent biological replicates were conducted in each group. The lentivirus containing a guide–target library (Lib-A) was transduced into AsCas12a-expressing HEK293T or RKO cells (4.0 × 10^6^ cells/dish, 100 mm dishes) in the presence of polybrene. The growth medium was replaced by fresh medium 24 h after lentivirus infection. Approximately 3 days post-transduction, the cells were harvested to sort for dual-fluorescence-positive cells (EGFP+/mCherry+) using FACS, which indicated the cells containing both a Cas12a effector and integrated target sites. Half of the collected cells were directly subjected to genome extraction as the sample for cleavage assay after 3 days, while the remaining cells were cultured in DMEM supplemented with 20% FBS, CFX, and penicillin-streptomycin and then harvested 5, 7, and 10 days after initial transduction, respectively. The collected cultures were subjected to genomic DNA isolation and target fragment amplification (including full-length integrated fragments) and prepared for deep sequencing. After 7 days of outgrowth from the lentiviral library infection, the editing rates of the target sites became nearly saturated and the repair outcome profiles tended to be stable. Therefore, the optimal outgrowth time for the following extensive cleavage assays used was 7 days.

For Cas12a editing assays at genomic integrated target sites: When following Strategy-i, Cas12a-expressing plasmids were transfected into an established cell library, as described for Cas12a delivery. The growth medium was replaced by a fresh medium 6 h after plasmid transfection. When following Strategy-ii, the lentivirus containing various guide–target libraries was transduced into established cell lines stably expressing certain Cas12a effectors. Cells from both strategies were cultured, and FACS was performed 3 days post-transfection/transduction, as described earlier for timeline cleavage assay. The sorted positive cells were cultured in DMEM supplemented with 20% FBS, CFX, and penicillin-streptomycin and then harvested at 7 days after plasmid transfection or lentivirus transduction. For each cell line, a control group was collected after the cell library was established but in the absence of Cas12a delivery.

For Cas12a editing assays at endogenous target sites: We selected 42 target sites with TTTV PAMs from Lib-A and synthesized their crRNA-encoding sequences individually from Tsingke Biotechnology (Beijing, China). The synthesized fragments were amplified into double strands with homologous arms and cloned into the pGPU6/GFP/Neo vector downstream from the U6 promoter. Prior to transfection, 1.5 × 10^6^ HEK293T cells were seeded into 48-well plates and cultured overnight until they were well attached. Plasmid-expressing Cas12a effectors and matched crRNAs were co-transfected into HEK293T cells using Lipofectamine™ 3000 Transfection Reagent, and the medium was replaced with a fresh medium of DMEM with 10% FBS. After 3 days of transfection, the cells were harvested for genome extraction and target fragment amplification (160–200 bp in total length including the target site edited by Cas12a) and were prepared for deep sequencing.

### 4.7. Deep Sequencing

Genomic DNA was isolated from the collected cell library using the TIANamp Genomic DNA Kit (DP304, TIANGEN, Bejing, China) for indel frequency analysis. Genome-integrated fragments including the guide and target sequences were PCR-amplified using KAPA HIFI HotStart ReadyMix and purified with a QIAquick Gel Extraction Kit.

To achieve >100× coverage over the guide–target library, we used 12 μg of genomic DNA per sample as the template for the first amplification. For a comparison between indel frequencies at integrated and endogenous sites, 100 ng of genomic DNA per sample was used to amplify the endogenous target sequences. For each sample, we prepared eight separate 20 μL reactions, with 1.5 μg genomic DNA for integrated target sites or with 12.5 ng for endogenous target sites as the template per reaction. The thermocycler was set for 1 cycle at 98 ${}^{\circ }$C for 45 s; for 25 cycles at 98 ${}^{\circ }$C for 15 s, 50 ${}^{\circ }$C for 20 s, and 72 ${}^{\circ }$C for 30 s; and for 1 cycle at 72 ${}^{\circ }$C for 1 min and held at 4 ${}^{\circ }$C. All of the resulting amplicons from the eight separate tubes were combined and purified as one sample.

A second amplification was performed to anneal both Illumina adapters and barcode sequences for deep sequencing. The resulting products were purified, mixed, and sequenced using Hiseq X Ten or NovaSeq 6000 (PE150) (Illumina, San Diego, CA, USA).

### 4.8. Analysis of Indel Frequency

For an analysis of the indel frequencies at endogenous target sites, we used CRISPResso2 (available online, https://crispresso.pinellolab.partners.org/submission (accessed on 22 January 2020)) [47] to analyze the genome editing outcomes and indel frequencies from deep sequencing data of the endogenous cleavage assays.

For the analysis of indel frequencies at genomic integrated target sites, given the specific design of a guide–target adjacent structure, current amplicon pool analyzers, such as CRISPResso2 or TIDE, were not capable of processing our data. Since the 23 nt spacer at both the guide region and target region were mapped as targets and confused the results of the editing outcome analysis, here, we provided customized Python scripts (Python script: Indel frequency analysis of Cas12a editing) to process and analyze the deep sequence data of the genome-integrated cleavage assays. A schematic workflow of the sorting and processing of deep-sequencing data and of further analyses is described in Supplementary Figure S2. The amplicons were aligned with a reference sequence of the crRNA–target libraries to count unedited and edited reads. Both the 42 nt crRNA-encoding sequence and the following 12 nt barcode were used to identify a unique guide RNA–target pair. Amplicons with mismatches in the crRNA-encoding region were considered invalid and excluded because editing efficiency could be impaired due to an imperfect match of crRNA with the target DNA strand.

For a primary analysis of the editing outcomes, deletions or insertions located inside a 42-bp frame around the expected cleavage site were recognized. Single-base substitutions were considered valid editing. We used a larger frame here to acquire a more complete observation of the overall Cas12a editing outcomes, for example, by avoiding missing large deletions spanning the expected cleavage region. The editing outcomes were categorized due to different repair types, and counted and recorded in the format of [DEL_start_pos, DEL_len]:read counts. For example, [(20, 6)]:111 referred to a repair type with a 6 bp deletion from positions 20 to 25 (the 5′ end of PAM sequence was set as position 0) with a read count of 111 in the processed data.

A secondary mutational analysis was performed based on the primary editing result by filtering out amplicons with only small indels outside the 8 bp frame centered on the middle of the cleavage site. These indels were most likely to be background noise that were not generated by Cas12a editing.

Both the control and experimental groups of the cleavage assays were subjected to the two-round mutational analysis above and resulted in a profile containing the editing outcome types and read counts of unedited and edited amplicons for each guide–target pair in the whole library.

To eliminate the background indels, we performed a differential analysis between the experimental and corresponding control group by removing all background mutated types. Only editing types that occurred uniquely in the experimental groups were considered Cas12a-induced mutations. The background indels mainly originated from oligonucleotide synthesis or were introduced during library construction and sequencing. After normalization, the final indel frequency of a certain target was calculated as edited read counts/(edited read counts + unedited read counts).

### 4.9. Statistical Significance

Statistical significance was determined using Graphpad Prism version 8.0.1(244). To select the appropriate statistical test for a certain set of data, descriptive statistics were conducted to measure the sample size, distribution type, dispersion, and centrality of the data.

To compare the indel frequencies between the two sets of data, we used the two-tailed Mann–Whitney U Test (U test) under the null hypothesis (H0) that the average indel frequencies of the two groups are the same (Figure 4).

To compare the indel frequencies among Cas12a orthologues or between different PAM groups, we used a non-parametric Kruskal–Wallis test (H test), followed by Dunn’s multiple comparison post hoc test (Figure 2c,d and Figure 5b,c).

The criterion for statistical significance was * *p* < 0.05, ** *p* <0.01, *** *p* < 0.001, and **** *p* < 0.0001. Since a *p* value less than 0.0001 was not provided by the software, only detailed *p* values above 0.0001 are shown in the figure captions.

### 4.10. Phylogenetic Tree Generated by MEGA 7.0.26v

Evolutionary analyses were conducted using MEGA 7.0.26v, and phylogenetic trees among the 16 Cas12a orthologues were constructed [48]. The coding data were translated into amino acid sequences assuming a standard genetic code table. All positions containing gaps and missing data were eliminated. Multiple-sequence alignment was carried out using the CLUSTAL W method in DNAstar MegAlign. The evolutionary history was inferred using the Neighbor-Joining method. The tree was drawn to scale, with the branch lengths in the same units as those of the evolutionary distances used to infer the phylogenetic tree. The evolutionary distances were computed using the Poisson correction method and were in the units of the number of amino acid substitutions per site.

### 4.11. Data and Code Availability

The authors declare that all data supporting the results in this study are available within the Supplementary Materials (Supplementary Note). The source code for the customized Python scripts used for guide RNA/target site design, and analysis of the Cas12a editing outcome and indel frequency are all available in the Supplementary Files: Python Scripts. The deep sequencing raw data from this study are available in the NCBI Sequence Read Archive (SRA) under BioProject accession code PRJNA773803.

## Data Availability

The data presented in this study are available in the manuscript and Supplementary Materials.

## Supplementary Materials

The following are available online at https://www.mdpi.com/article/10.3390/ijms222413301/s1.

## Abbreviations

PAM	Protospacer adjacent motif
crRNA	CRISPR RNA, short guide RNAs for Cas effector targeting
DR	Direct repeats, repetitive sequence identified before spacers
DSB	Double-strand break
Indel	Insertions or/and deletions, generated at target sites by Cas12a editing
NHEJ	Non-homologous end joining
MMEJ	Microhomology-mediated end joining
3Rv	Cas12a variants with a combination of three arginine substitutions
