# Supplementary material for: High-Throughput Profiling of Cas12a Orthologues and Engineered Variants for Enhanced Genome Editing Activity
Source: Int J Mol Sci. 2021 Dec 10;22(24):13301. doi: 10.3390/ijms222413301 (PMC8706968; doi:10.3390/ijms222413301)
Supplement: Supplementary file 1 [file ijms-22-13301-s001.zip › ijms-1460346-supplementary/Supplementary Figure S1-S9 +Table S1-S4.pdf]

Supplementary Figures S1-S10

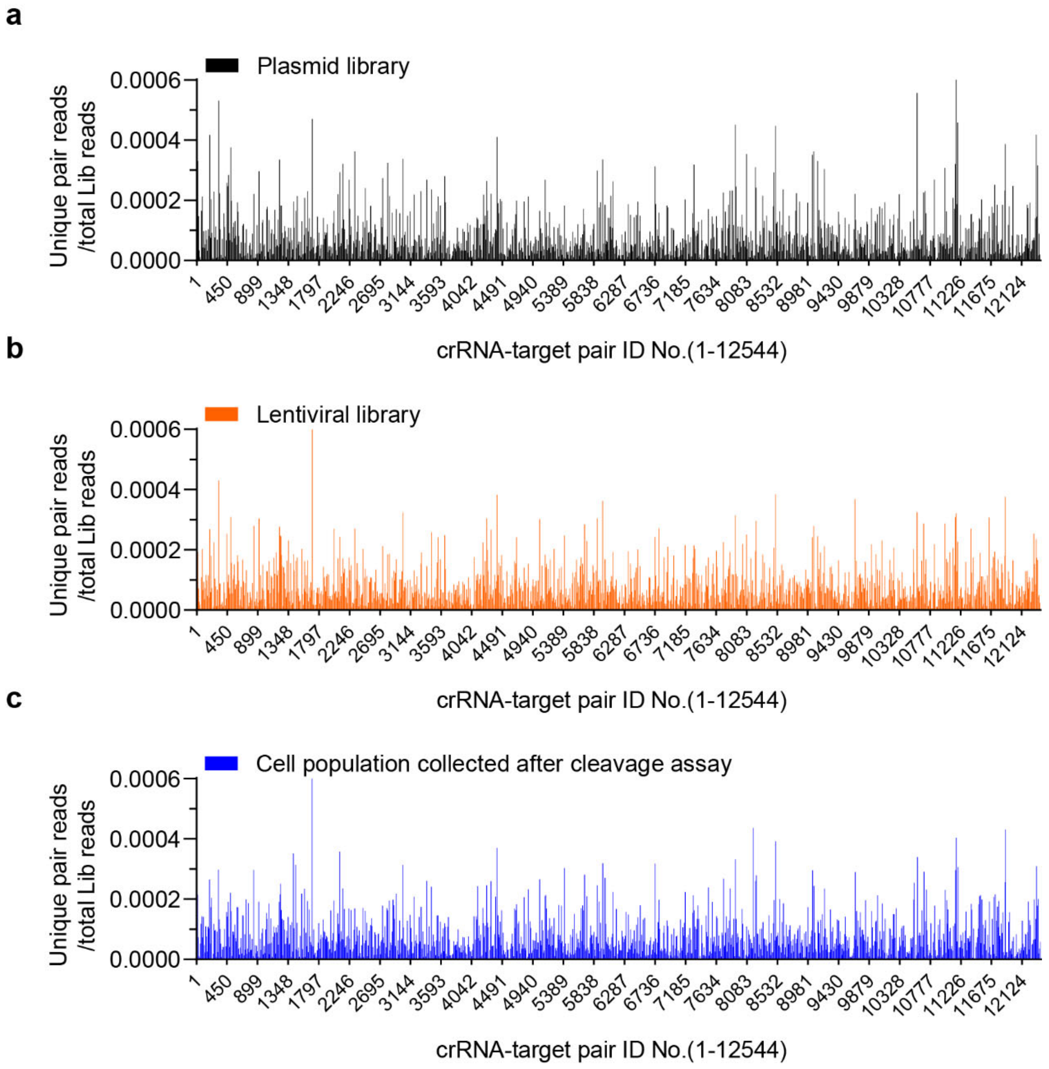

Supplementary Figure S1. Related to Figure 1a: Uniformity assessment of the constructed plasmid library and lentiviral library integrated in a cell genome by deep sequencing.

The composition of three libraries (plasmid library, and lentivirus library before and after cleavage assay) exhibited similar patterns on the read ratios of unique pairs in the whole library, which means no apparent enrichment or depletion of crRNA–target pairs occurred during the procedure, hence maintaining the uniformity and diversity of the library.

Supplementary Figure S2. Schematic workflow of high-throughput sequencing (HTS) data

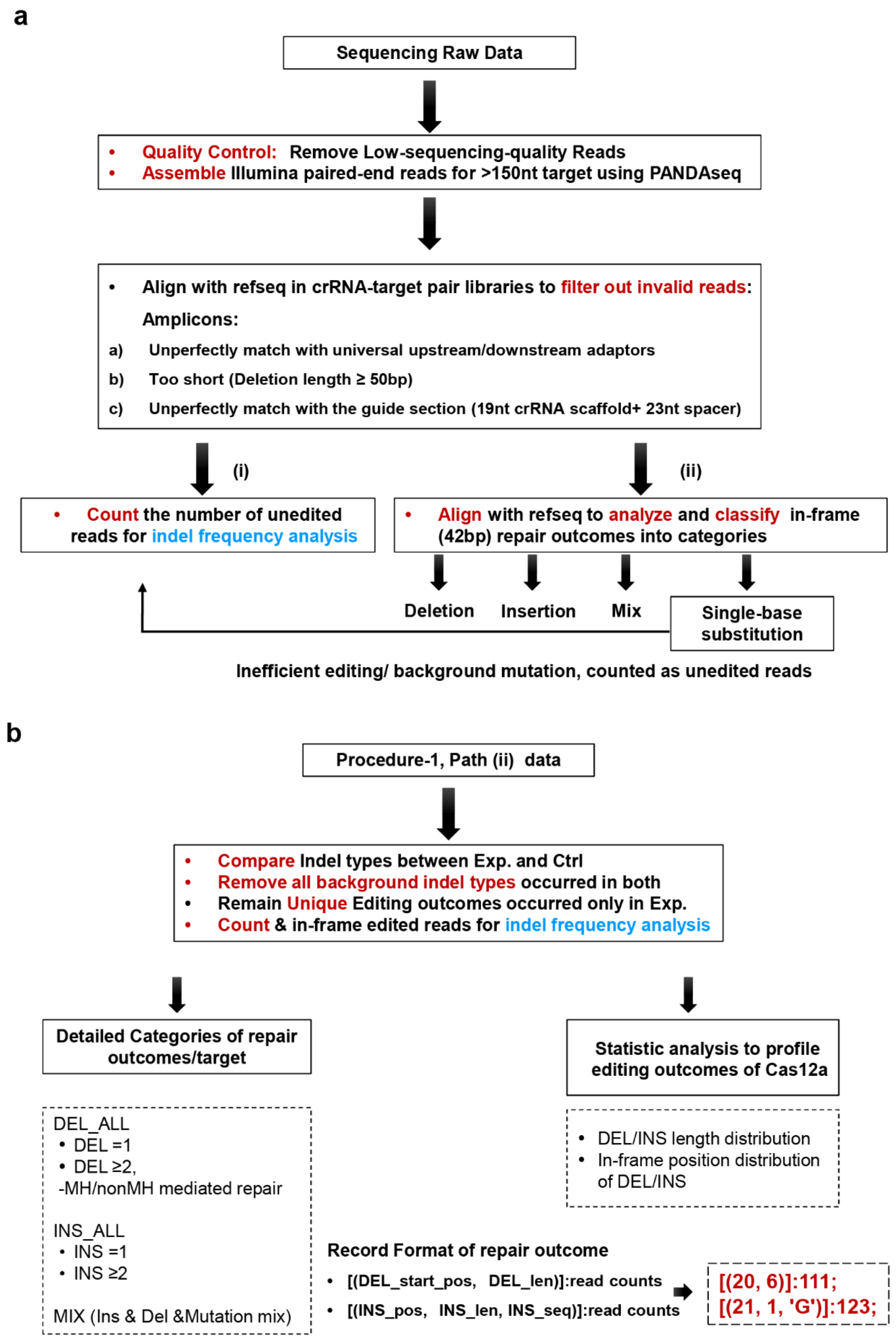

processing for editing outcome profiling and indel efficiency evaluation of Cas12a effectors.

(a) Procedure 1: Filter for high-confidence sequencing reads, align the reads with the reference

sequences of crRNA–target libraries to count unedited and edited reads, and categorize mutational outcomes in all control and experimental groups. (QC: Q20>95%). **(b)** Procedure 2: Conduct a differential analysis between the experimental and corresponding control groups to remove all background mutated types. The background indels mainly originated from oligonucleotide synthesis or were introduced during library construction and sequencing.

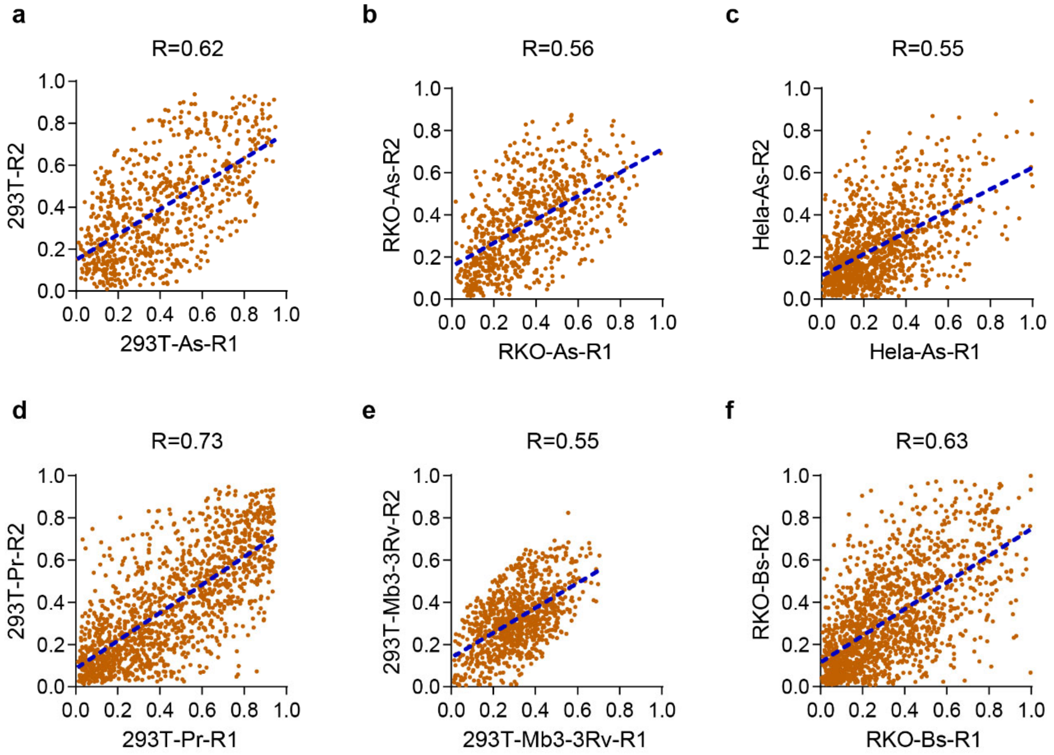

Supplementary Figure S3. Related to Figure 1: Editing activities of various Cas12a orthologues and variants measured at integrated target sites in a high-throughput manner are reproducible in diverse cell lines.

The scatter plots indicate measurements of indel frequencies for the same target site generated by Cas12a orthologues or variants from two independent biological replicates (R1 and R2). Cell type, Cas12a orthologue/variant and crRNA–target pair number involved in each plot: **(a)** HEK293T, AsCas12a, *n*=748; **(b)** RKO, AsCas12a, *n*=755; **(c)** Hela, AsCas12a, *n*=1,156; **(d)** HEK293T, PrCas12a, *n*=1,421; **(e)** HEK293T, Mb3Cas12a-3Rv (D180R/N581R/K587R), *n*=933; and **(f)** RKO, BsCas12a, *n*=1,527. The indel frequencies range from 0 to 1. Pearson (r) correlation coefficients are shown.

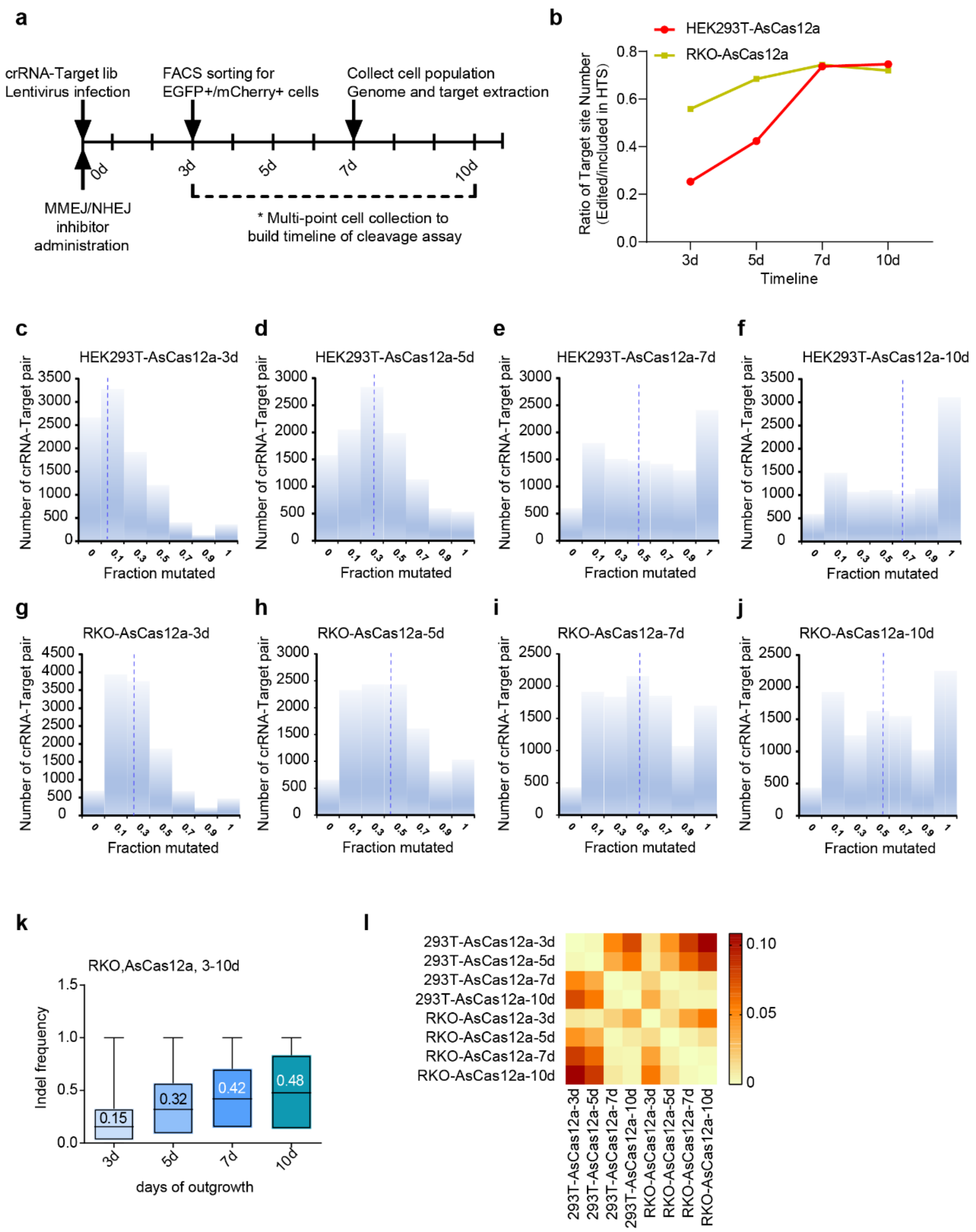

Supplementary Figure S4. Timeline of the Cas12a cleavage assay： The editing rates are saturated, and the repair outcomes are stable after 7-10 days of outgrowth. The assay was used to determine the appropriate outgrowth time for editing efficiency measurement.

(a) Schematic of a workflow for multiple-time-point cell collection to build an editing timeline generated by Cas12a effectors. Cell population collection was performed after 3, 5, 7, and 10 days of Cas12a editing, followed by genome extraction and target fragment amplification for deep sequencing. **(b)** Ratio of the change in edited target site/total designed crRNA–target pairs generated

by different effectors for AsCas12a in HEK293T or RKO cells after different outgrowth times. **(c–j)** Histogram of editing rate measurements generated by AsCas12a at four time points (3, 5, 7, and 10 days) after the lentivirus library of crRNA–target pairs infected HEK293T (c–f) and RKO (g–j). y-axis: number of crRNA–target pairs; x-axis: fraction of mutated reads. The dashed line denotes the median fraction of mutated reads. **(k)** Indel frequency distribution of the whole crRNA–target library after different outgrowth times (corresponding to the dataset in (g–j) is shown as a box-whisker plot. Median line with the median value marked, quartiles for box edges, and min and max for the bottom and top whiskers. **(l)** Heatmap of symmetric KL divergence: difference between editing outcomes generated by AsCas12a as outgrowth time prolonged. KL divergence (Kullback–Leibler Divergence) was introduced to measure the difference between two editing outcome profiles at different time points of the Cas12a cleavage assay [1]. The probability distribution of editing outcome profiles consisted of six values referring to the frequencies of certain repair types. The KLd values were calculated using the philentropy package in R project (version 4.1.1) as follows: symmetry KL = KL(P||Q) + KL(Q||P).

To determine the appropriate outgrowth time for measuring Cas12a editing efficiency, a timeline of cleavage assay was built by setting multiple collection time points of the cell population during the cleavage assay (3, 5, 7, and 10 days post-editing). After 7 days of outgrowth from the lentiviral library infection, the editing rates of target sites became nearly saturated and the repair outcome profiles tended to be stable. If collection was performed too early, such as less than 5 days of outgrowth, the editing of target sites in the library may be far from sufficient due to the different editing efficiencies of the Cas12a effectors, hence leading to false or invalid evaluation results. If collection was performed too late, such as over 10 days of outgrowth, over-expansion of the cell population may cause biased enrichment or depletion of certain crRNA–target pairs, hence damaging the uniformity and diversity of the library. Therefore, an appropriate outgrowth time for the extensive profiling of Cas12a editing activity and repair outcomes is 7 days.

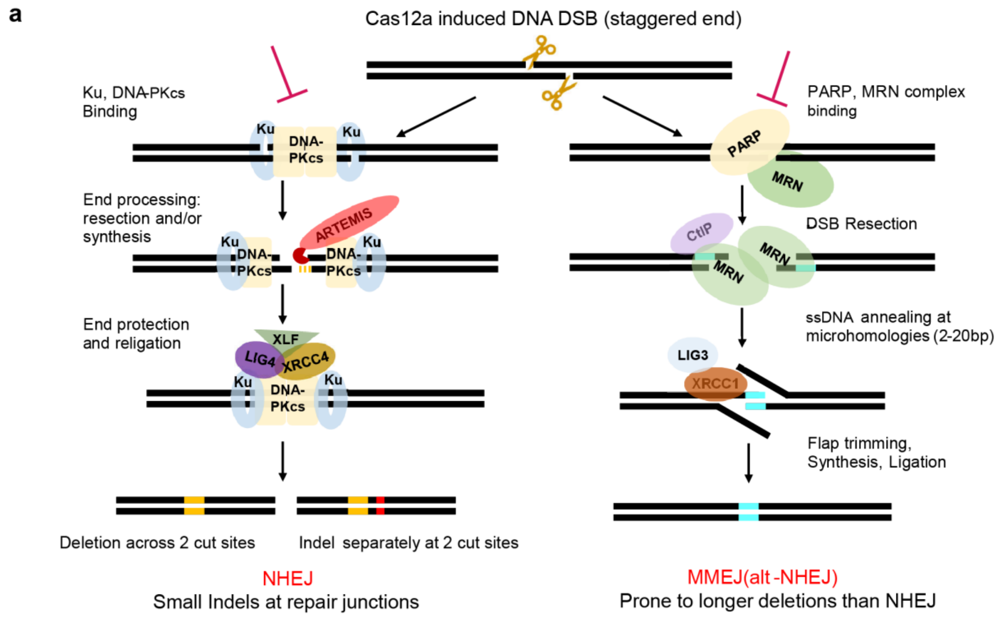

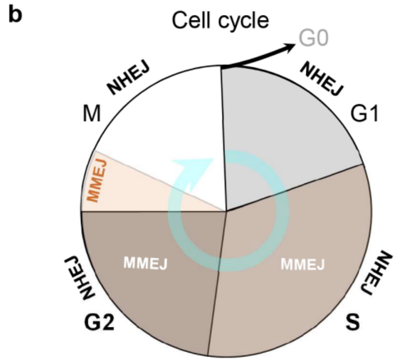

c

NHEJ	MMEJ
• Ku70/80	• PARP1
• DNA-PKcs	• MRN complex
• DNA Ligase IV	• CtIP
• XRCC4	• DNA Ligase III
• XLF	• XRCC1
• Artemis	• NBS1
• DNA pol μ	• FEN1
• DNA pol λ	• RPA
• PNKP	• DNA pol θ
• Aprataxin	
• APLF	
• BRCA1	
• BRCA2	
• CYREN	

Supplementary Figure S5. Related to Figure 1: Alternative DNA repair pathways involved in the repair of DSB generated by template-free Cas12a editing.

(a) NHEJ starts with rapid recognition of DNA breaks by the Ku70/80 complex, which recruits DNA-PKcs. Sequential autophosphorylation of DNA-PKcs recruits nucleases such as ARTEMIS to initialize processing of DNA ends, followed by DNA-PKcs-dependent end protection and broken end regulation promoted by the XRCC4–DNA Ligase IV–XLF complex. NHEJ prefers small insertions/deletions (1-5 bp) at the repair junctions, which leads to single or separate repairs at two or more cut sites with an over 5 bp interval generated by Cas12a. **Alt-NHEJ/MMEJ** functions as a backup repair mechanism for c-NHEJ. In MMEJ, PARP competes with the Ku complex to bind DNA breaks, which promotes MRN complex accumulation. CtIP, which interacted with the MRN complex, carries out limited DSB resection to generate ssDNA for microhomology region annealing. XRCC1-DNA Ligase III promotes post-annealing flap trimming, strand extension, and regulation. After Cas12a cleavage MMEJ yields repair products with various deletion sizes (2-20 bp), favoring

longer deletions compared with NHEJ products. **(b)** Preferred repair mechanisms during the cell cycle after DSB. NHEJ is active during the whole cell cycle, while MMEJ activity is low in G1 and significantly elevated in S/G2 and the early M stages. **(c)** Proteins involved in the mechanism or regulation of NHEJ and MMEJ in human cells. The red text denotes the key functional proteins in NHEJ or MMEJ targeted by the three inhibitors used in this work (Ni-1, Ni-2: DNA-PKcs; and Mi: PARP1) tested for the influence of genetic background on the editing efficiency of Cas12a.

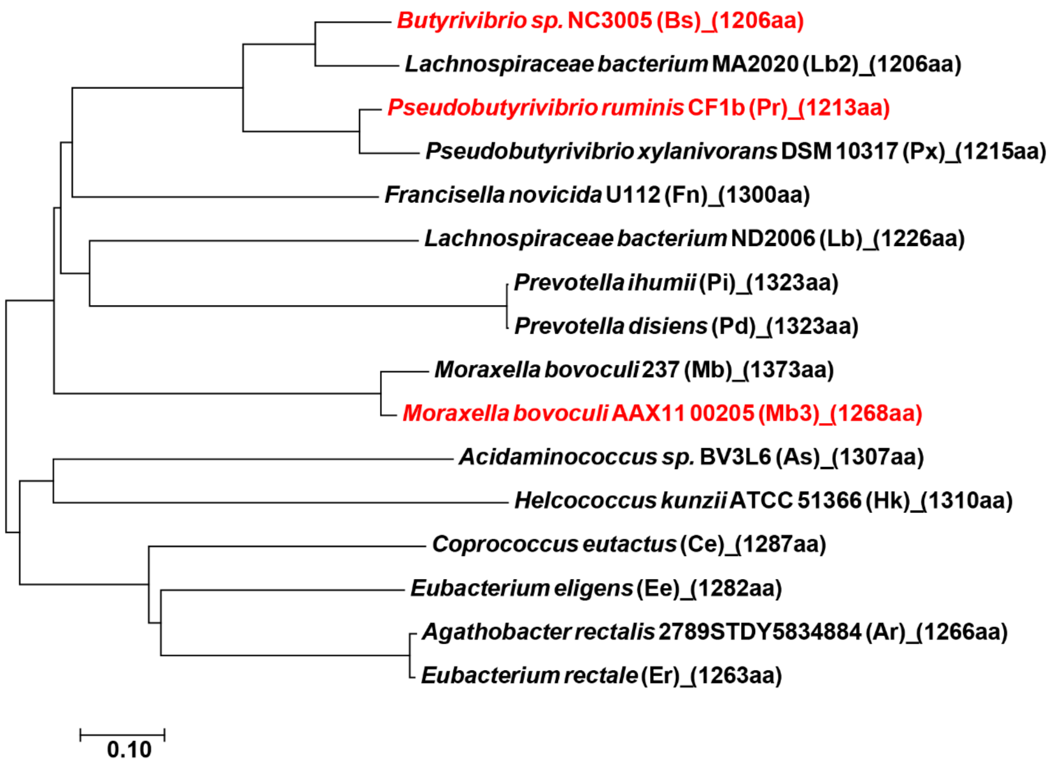

Supplementary Figure S6. Related to Figure 2a: Detailed phylogenetic tree of 16 Cas12a orthologues analyzed in this work was constructed using MEGA7.0.26.

All of the 16 Cas12a orthologues evaluated in this work have been previously confirmed with editing activity for gene manipulation in human cells. The source bacteria strain and the protein size of Cas12a effectors are presented. Three Cas12a, shown in red, were candidates chosen to generate the engineered Cas12a variants in this work. Multiple sequence alignment method Clustal W was performed to measure the evolutionary distance between proteins based on the divergence of amino acid sequences. The evolutionary history was inferred using the Neighbor-Joining method. The evolutionary distances between Cas12a effectors are indicated by branch length. The evolutionary distances were computed using the Poisson correction method and drawn to scale with branch lengths.

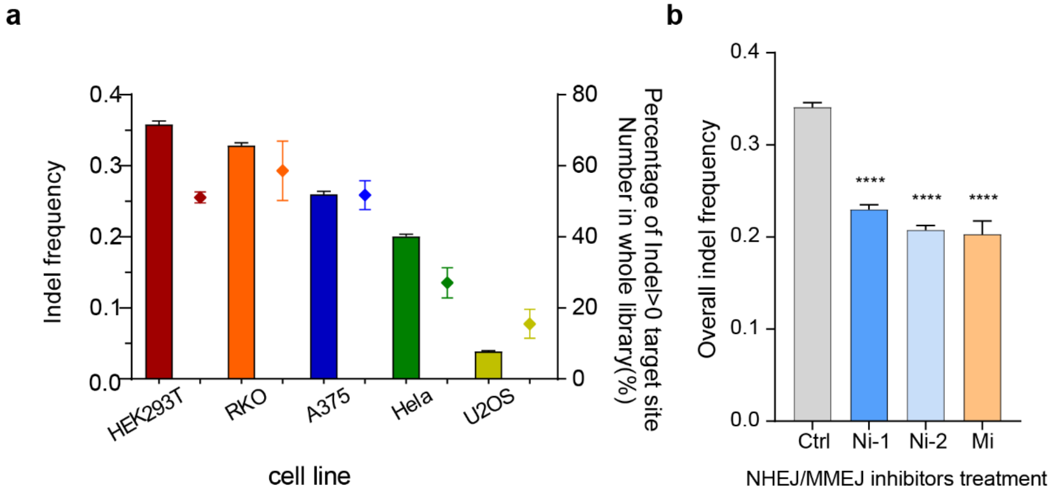

Supplementary Figure S7. Related to Figure 2a: Genetic background has influence on Cas12a editing.

(a) Editing activity assessment of AsCas12a at 3,303 target sites with TTTV PAMs presented in all five cell lines (HEK293T, RKO, U2OS, Hela, and A375). Left y-axis: Bar plots show average editing frequencies (Mean ± s.e.m.). Right y-axis: mean of the edited target sites ratio (%) of two independent biological replicates shown in a diamond symbol, also reflecting the overall AsCas12a editing activity in certain cell lines. **(b)** Editing activity assessment of AsCas12a at 727 target sites with TTTV PAMs presented in both biological replicates of the control and experimental groups treated with NHEJ/MMEJ inhibitors. Inhibitor administration created a biased genetic background featured as varying preferences for the repair pathway for DSB after Cas12a cleavage. Ni-1 denotes NU7441 (2μM), Ni-2 denotes Ku-0060648 (250nM), and Mi denotes Rucaparib (10μM). The target sites involved here had at least 100 read counts per crRNA–target pair in a synthetic sample with indel frequency >0. Differences between the control and inhibitor treated groups were determined by a two-tailed Mann–Whitney test (U test). ****$p < 0.0001$.

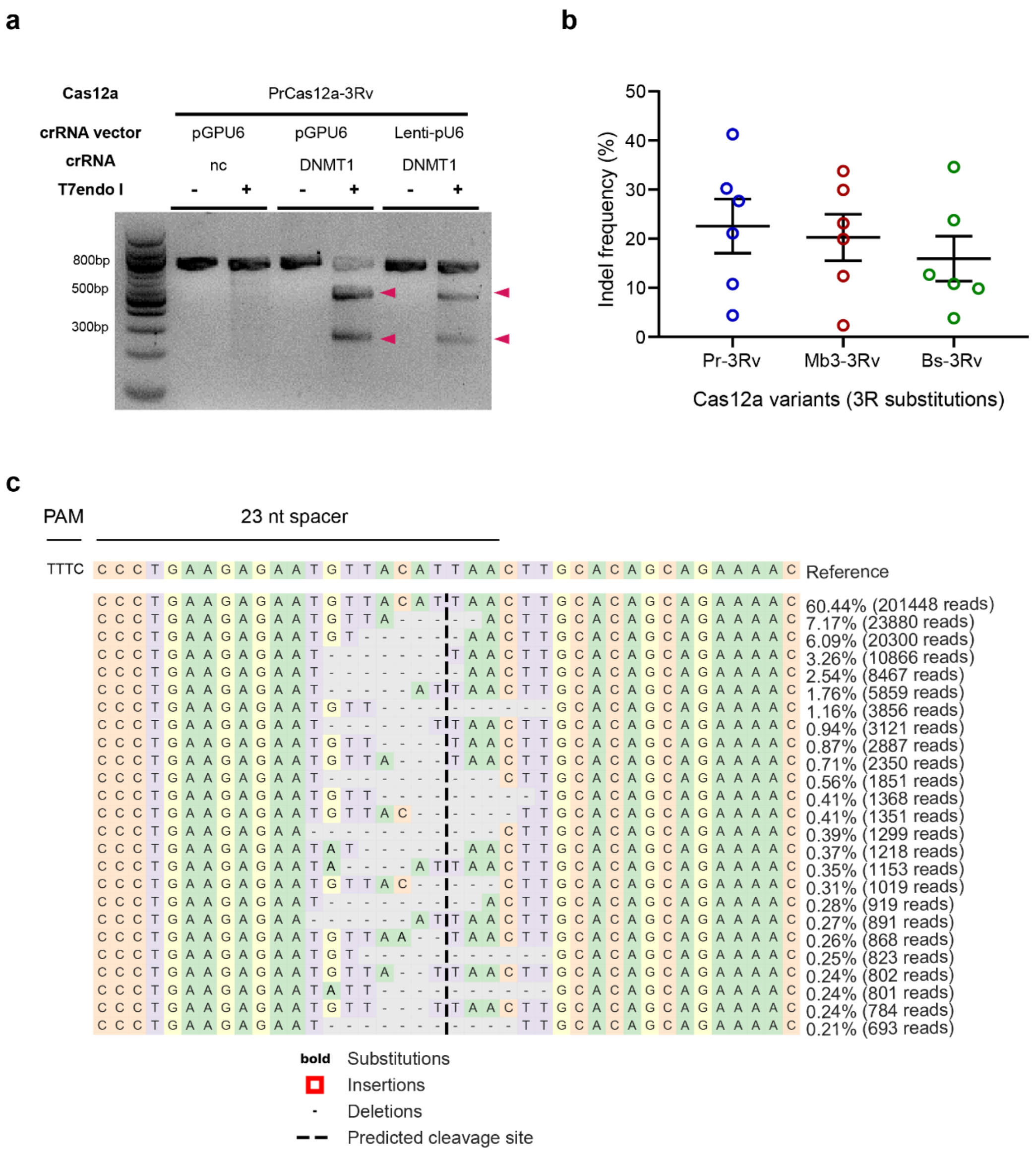

Supplementary Figure S8. Related to Figure 4: Editing activity of engineered Cas12a (3Rv) on endogenous target sites in HEK293T.

(**a**) Two crRNA-expressing vectors (pGPU6-crRNA or Lenti-pU6-crRNA) were tested for endogenous cleavage assays. Plasmid expressing PrCas12a-3Rv and vector encoding crRNA were co-transfected to HEK293T cells and targeted an endogenous site in *DNMT1*. Indel frequencies were detected by T7E1 assay after 3 days of editing. The red triangles indicate cleaved fragments (485bp/256bp). The pGPU6-crRNA group exhibited the most robust editing efficiency and was adopted for further endogenous cleavage assay in (**b**). **(b)** PrCas12a-3Rv, Mb3Cas12a-3Rv, and BsCas12a-3Rv were tested for endogenous editing activity in HEK293T using six guides from Lib-A towards corresponding endogenous target sites with TTTV PAMs (V = A/C/G). Indel frequencies were determined by deep sequencing and analyzed using CRISPResso2. Each data point represents

the average of two biological replicates for one guide. The data are shown as means with SEMs. For exact values of the average indel frequencies, see the Supplementary Dataset. (**c**) The schematic of the CRISPResso2 analysis result was represented as an alleles frequency table around the guide RNA, using the editing outcome of PrCas12a-3Rv targeting *ALCAM* (target site sequence (4 nt PAM + 23 nt spacer): TTTCCCCTGAAGAGAATGTTACATTAA).

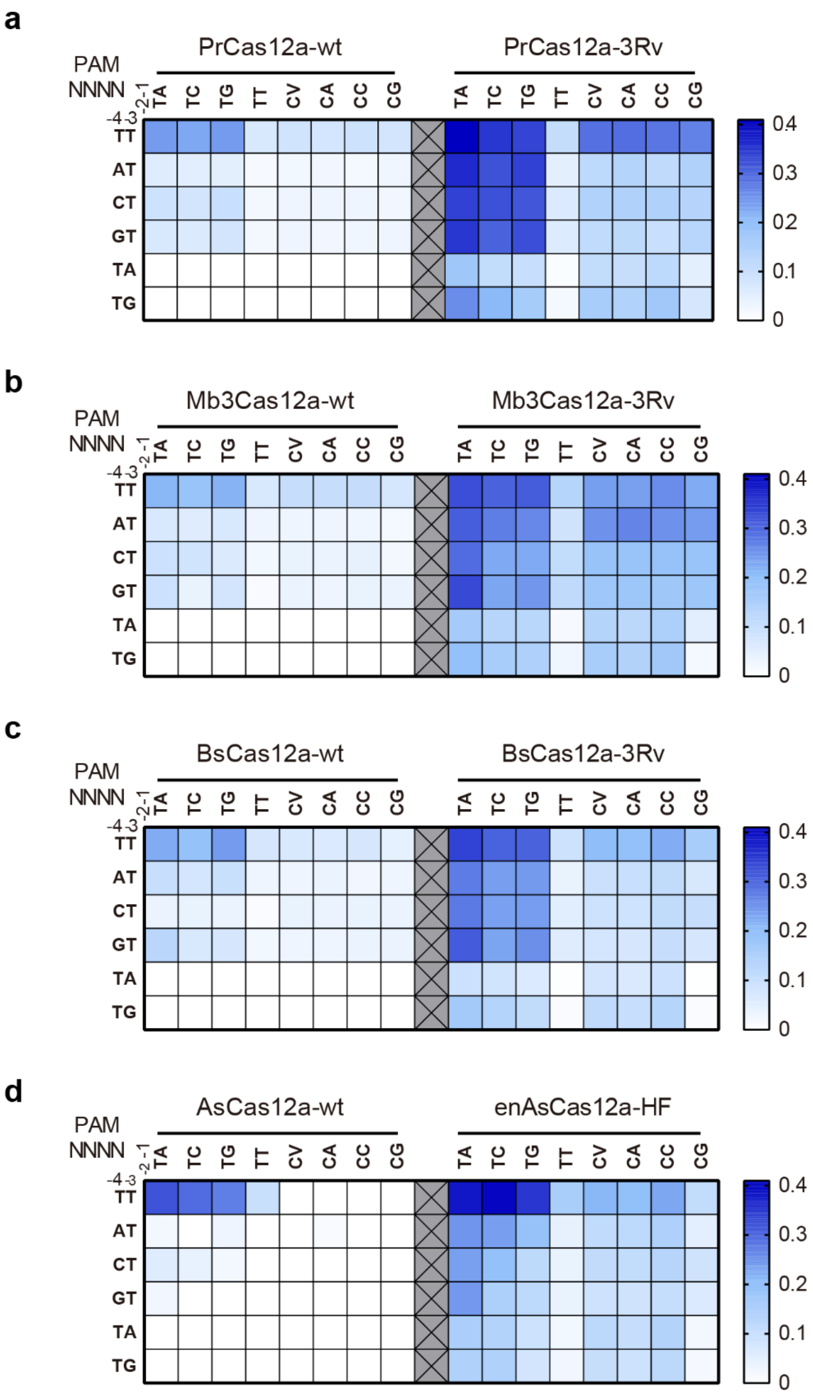

Supplementary Figure S9. Related to Figure 5a: Comparison of recognition range between wild-type Cas12a and engineered variants.

Extensive comparison of PAM compatibility between engineered Cas12a-3Rv and corresponding wild-type Cas12a effectors. **(a)** PrCas12a versus PrCas12a-3Rv; **(b)** Mb3Cas12a versus Mb3Cas12a-3Rv; **(c)** BsCas12a versus BsCas12a-3Rv; and **(d)** AsCas12a versus enAsCas12a-HF.

enAsCas12a-HF (E174R/N282A/S542R/K548R) contains the combination of arginine substitutions; hence, it was adopted here as the 3R version of AsCas12a. V = A/C/G. The values in the heatmap indicate the indel frequencies of Cas12a at target sites with different PAM sequences. The white-to-dark-blue gradient represents increasing editing efficiency. The indel frequencies were determined by deep sequencing. The target sites involved here had at least 300 read counts per crRNA–target pair in one sample. For exact values of the average indel frequencies, see the Supplementary Dataset.

Supplementary Table S1-4

Supplementary Table S1. Related to Figure 1a: Quality assessment of the library construction, assay design, and sequencing: measured with library coverage, and reads counts before and after filtering.

Library for quality assessment	Paired guide–target library construction		Cell population collected after cleavage assay	
	Plasmid Library	Lentiviral Library	Two Cleavage assay designs	
			Design (i)	Design (ii)
**Number of designed pairs**	12,544			
**Number of included pairs (read count ≥ 10/pair)**	12,449	12,390	12,037	12,114
**A percentile of included/designed (%)**	99.2%	98.8%	96.0%	96.6%
**Number of total valid reads by deep sequencing (after filtering)**	7,107,414	4,795,554	4,782,716	4,569,404
**Raw data read count/Lib**	12,419,408	17,043,942	16,581,918	16,485,590
**A percentile of available reads for downstream indel analysis (%)**	57.2%	28.1%	28.8%	27.2%

Design (i): first, a lentivirus infection was used to construct a cell line with the genome-integrated crRNA–target library, followed by transfection of a plasmid expressing diverse Cas12a effectors. **Design (ii)**: first, a lentivirus infection was used to construct a Cas12a-expressing cell line, followed by lentivirus integration of the crRNA–target library. The data in the table were collected through the construction of a paired crRNA–target library with TTTV PAMs and cleavage assays with AsCas12a following two strategies. Coverage for the crRNA–target library and available read counts after three critical steps (plasmid library construction, lentivirus library construction, and cleavage assay) in the workflow is depicted. Designs (i) and (ii) exhibited similar coverages for the crRNA–target library (96.0% versus 96.6%). After filtering the raw data, 4.6–4.8 million read counts

remained available for each library (~12k pairs), which means about 400 effective reads per crRNA–target pair.

To assess the quality of the guide–target library construction, we collected the plasmid library, the cell library (integrated into HEK293T cells) of Lib-A, and the samples after cleavage assays following two strategies for high-throughput sequencing. The deep-sequencing data showed that, among the 12,544 designed pairs, 12,449 (99.2%) and 12,390 (98.8%) pairs were included in the plasmid and cell library, respectively. In contrast, after the cleavage assays, 12,037 (96.0%) (i) and 12,114 (96.6%) (ii) pairs were included in the cells, suggesting a relatively high quality of library construction with integrity and uniformity.

Supplementary Table S2. Summary of Cas12a effectors (orthologues and variants) involved in this work.

Code	Category	Cas12 orthologues/variants	PAM recognition
P1	Cas12a orthologue	AsCas12a[2]	TTTV
P2		LbCas12a[2]	TTTV
P3		FnCas12a[2]	NTTV
P4		MbCas12a[2]	NTTV
P5		BsCas12a[3]	NTTN
P6		HkCas12a[3]	YTN,TYN,TTYN,TCCN
P7		ArCas12a[3]	NTTN
P8		PrCas12a[3]	NTTN
P9		PxCas12a[3]	NTTN
P10		Mb3Cas12a[4,5]	NTTN
P11		PdCas12a[6]	TTTV

P12		PiCas12a[6]	KKYV
P13		Lb2Cas12a[7]	TTTV,CTTN
P14		ErCas12a[8,9]	YTTN
P15		EeCas12a[10]	TTTN
P16		CeCas12a[11]	TTTV
P17	**Cas12a variant**	enAsCas12a-HF (E174R/N282A/S542R/K548R）[12]	NTTV,TTCV,TRTV
P18	**Cas12a variants generated in this work**	BsCas12a-3Rv(K155R/N512R/K518R)	NTTV,TTCV,TGTV
P19		PrCas12a-3Rv(E162R/N519R/K525R)	NTTV,TTCV,TRTV
P20		Mb3Cas12a3Rv(D180R/N581R/K587R)	NTTV,NTCV,TRTV

P1–P17 are the previously reported 16 Cas12a orthologues and 1 engineered Cas12a variant, which were employed for genome editing on endogenous sites in human cells. P18–P20 are Cas12a variants with three arginine substitutions generated in this study for improved editing efficiency and expanded PAM recognition range. V = A/C/G; N = A/C/G/T; K = G/T; R = A/G.

Supplementary Table S3. Summary of guide RNA design and target site selection of crRNA–target pair libraries with diverse PAM sequences.

Library ID	Lib-A	Lib-B	Lib-C
**Cas12a effector**	All 16 wild-type Cas12a orthologues	4 Cas12a variants	
**5' end scaffold design of crRNA**	Wild-type crRNA scaffold corresponding to each Cas12a orthologue [a] or optimized crRNA scaffold with a variant loop region [UAUG] [b]		
**Target site design**	Top 10 crRNA candidates/genes designed by an online guide predictor (CRISPR RGEN Tools) [c]	In-house Python scripts (Python 3.6.5) modified to design target sites with non-canonical PAMs [d]	

**PAM composition/ Library**	All TTTV(12,544)	VTTV(1,508), TTCV(2,411), VTCV(2,890), VCTV(3,013), VCCV(2,552), PCtrl(169) [e]	TATN(5,429), TGTN(6,071), NTTT(662), PCtrl(201) [e]
**Target gene**	Transcripts (5'UTR, CDS, 3'UTR) of 1,383 hepatoma and colorectal cancer-associated human genes, derived from the Ensembl database		

In the ‘PAM composition column’, the numbers inside the parentheses represent the number of target sites with a certain type of PAM sequence in one library (total 12,544 target sites/library). The letter codes for base degeneracy in PAMs: V = A/C/G; N = A/C/G/T; R = A/G. For details of **a** and **b**, see Supplementary Table S5. The wild-type crRNA scaffolds derived from the DR repeat region of original strains for 16 Cas12a orthologues are listed in Figure 2b [2–11].
c. The CRISPR RGEN Tools->Cpf1-database contains genome-wide available targets with 5’-TTTN-3’ PAM sequences in coding sequence (CDS) regions recognized by Cas12a effectors. (http://www.rgenome.net/cpf1-database/) [13,14].
d. For the in-house code used to design guide RNA for Cas12a with non-canonical PAMs, see the Python script for design targets with an altered PAM package in the Supplementary Materials.
e. PCtrl means positive control target sites, which are human genome-derived target sites that have already been reported to have good editing efficiencies in human cells in previous Cas12a activity studies [3].

Reference:

1. Allen, F.; Crepaldi, L.; Alsinet, C.; Strong, A.J.; Kleshchevnikov, V.; De Angeli, P.; Páleníková, P.; Khodak, A.; Kiselev, V.; Kosicki, M.; Jackson, S.P.; Parts, L. Predicting the Mutations Generated by Repair of Cas9-Induced Double-Strand Breaks. *Nat. Biotechnol.* **2019**, *37* (1), 64–82.
2. Zetsche, B.; Gootenberg, J.S.; Abudayyeh, O.O.; Slaymaker, I.M.; Makarova, K.S.; Essletzbichler, P.; Volz, S.E.; Joung, J.; Van Der Oost, J.; Regev, A.; Koonin, E. V.; Zhang, F. Cpf1 Is a Single RNA-Guided Endonuclease of a Class 2 CRISPR-Cas System. *Cell* **2015**, *163* (3), 759–771.
3. Teng, F.; Li, J.; Cui, T.; Xu, K.; Guo, L.; Gao, Q.; Feng, G.; Chen, C.; Han, D.; Zhou, Q.; Zhou, Q.; Li, W. Enhanced Mammalian Genome Editing by New Cas12a Orthologs with Optimized CrRNA Scaffolds. *Genome Biol.* **2019**, *20* (1), 15.
4. Zetsche, B.; Strecker, J.; Abudayyeh, O.O.; Gootenberg, J.S.; Scott, D.A.; Zhang, F. A Survey of Genome Editing Activity for 16 CAS12A Orthologs. *Keio J. Med.* **2020**, *69* (3), 59–65.
5. Wang, Z.; Wang, Y.; Wang, S.; Gorzalski, A.J.; McSwiggin, H.; Yu, T.; Castaneda-Garcia, K.;

Prince, B.; Wang, H.; Zheng, H.; Yan, W. Efficient Genome Editing by CRISPR-Mb3Cas12a in Mice. *J. Cell Sci.* **2020**, *133* (9), jcs240705.

6. Jacobsen, T.; Ttofali, F.; Liao, C.; Manchalu, S.; Gray, B.N.; Beisel, C.L. Characterization of Cas12a Nucleases Reveals Diverse PAM Profiles between Closely-Related Orthologs. *Nucleic Acids Res.* **2020**, *48* (10), 5624–5638.
7. Tran, M.H.; Park, H.; Nobles, C.L.; Karunadharma, P.; Pan, L.; Zhong, G.; Wang, H.; He, W.; Ou, T.; Crynen, G.; Sheptack, K.; Stiskin, I.; Mou, H.; Farzan, M. A More Efficient CRISPR-Cas12a Variant Derived from Lachnospiraceae Bacterium MA2020. *Mol. Ther. Nucleic Acids* **2021**, *24*, 40–53.
8. Wierson, W.A.; Simone, B.W.; WareJoncas, Z.; Mann, C.; Welker, J.M.; Kar, B.; Emch, M.J.; Friedberg, I.; Gendron, W.A.C.; Barry, M.A.; Clark, K.J.; Dobbs, D.L.; McGrail, M.A.; Ekker, S.C.; Essner, J.J. Expanding the CRISPR Toolbox with ErCas12a in Zebrafish and Human Cells. *Cris. J.* **2019**, *2* (6), 417–433.
9. Liu, Z.; Schiel, J.A.; Maksimova, E.; Strezoska, Ž.; Zhao, G.; Anderson, E.M.; Wu, Y.; Warren, J.; Bartels, A.; van Brabant Smith, A.; Lowe, C.E.; Forbes, K.P. ErCas12a CRISPR-MAD7 for Model Generation in Human Cells, Mice, and Rats. *Cris. J.* **2020**, *3* (2), 97–108.
10. Ahn, W.C.; Park, K.H.; Bak, I.S.; Song, H.N.; An, Y.; Lee, S.J.; Jung, M.; Yoo, K.W.; Yu, D.Y.; Kim, Y.S.; Oh, B.H.; Woo, E.J. In Vivo Genome Editing Using the Cpf1 Ortholog Derived from Eubacterium Eligens. *Sci. Rep.* **2019**, *9* (1), 13911.
11. Chen, P.; Zhou, J.; Wan, Y.; Liu, H.; Li, Y.; Liu, Z.; Wang, H.; Lei, J.; Zhao, K.; Zhang, Y.; Wang, Y.; Zhang, X.; Yin, L. A Cas12a Ortholog with Stringent PAM Recognition Followed by Low Off-Target Editing Rates for Genome Editing. *Genome Biol.* **2020**, *21* (1), 78.
12. Kleinstiver, B.P.; Sousa, A.A.; Walton, R.T.; Tak, Y.E.; Hsu, J.Y.; Clement, K.; Welch, M.M.; Horng, J.E.; Malagon-Lopez, J.; Scarfò, I.; Maus, M. V.; Pinello, L.; Aryee, M.J.; Joung, J.K. Engineered CRISPR–Cas12a Variants with Increased Activities and Improved Targeting Ranges for Gene, Epigenetic and Base Editing. *Nat. Biotechnol.* **2019**, *37* (3), 276–282.
13. Park, J.; Kim, J.S.; Bae, S. Cas-Database: Web-Based Genome-Wide Guide RNA Library Design for Gene Knockout Screens Using CRISPR-Cas9. *Bioinformatics* **2016**, *32* (13), 2017–2023.
14. Park, J.; Bae, S. Cpf1-Database: Web-Based Genome-Wide Guide RNA Library Design for Gene Knockout Screens Using CRISPR-Cpf1. *Bioinformatics* **2018**, *34* (6), 1077–1079.
