# Supplementary material for: High-Throughput Profiling of Cas12a Orthologues and Engineered Variants for Enhanced Genome Editing Activity
Source: Int J Mol Sci. 2021 Dec 10;22(24):13301. doi: 10.3390/ijms222413301 (PMC8706968; doi:10.3390/ijms222413301)
Supplement: Supplementary file 1 [file ijms-22-13301-s001.zip › ijms-1460346-supplementary/Supplementary Note.pdf]

Supplementary Note

Plasimid maps and sequences 1
Humanized Coding sequences of 20 Cas12a effectors........ 6
Detailed legend for Figures 1-5 27
Related to Figure 1 27
Related to Figure 2 28
Related to Figure 3 28
Related to Figure 4 28
Related to Figure 5 29

Plasmid maps and sequences

(1) Lenti-pU6-crtslib-EGFP (b): Backbone plasmid for the construction of paired guide–target libraries, modified from the following plasmid: P08-pU6-PDL1-pCMV-EGFP (a).

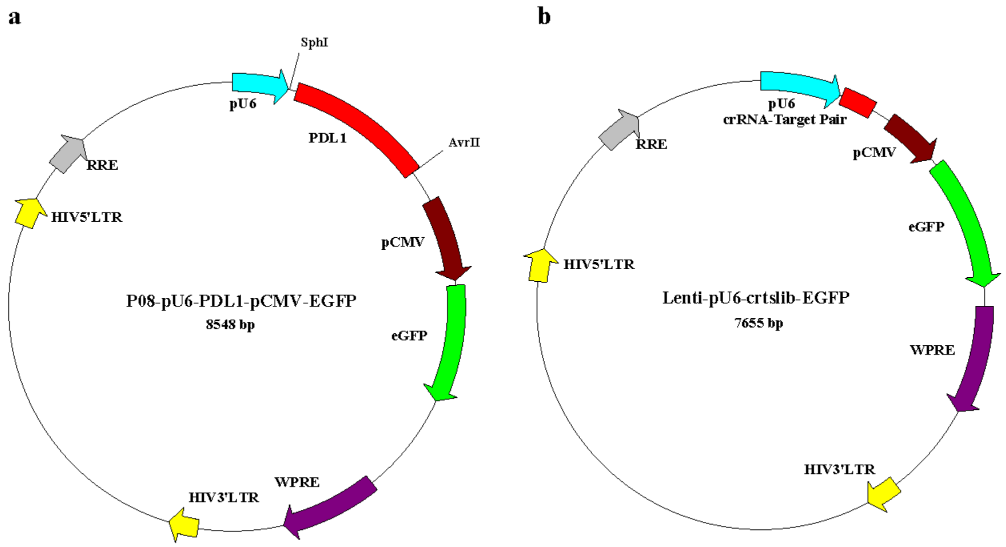

- U6 promoter
- SphI restriction site
- AvrII restriction site
- EcoRI restriction site
- XbaI restriction site

GAGGGCCTATTTCCCATGATTCCTTCATATTTGCATATACGATACAAGGCTGTTAGAGAGATAATTAGAATTAATT
TGACTGTAAACACAAAGATATTAGTACAAAATACGTGACGTAGAAAGTAATAATTTCTTGGGTAGTTTGCAGTTTT

TAAAATTATGTTTTAAAATGGACTATCATATGCTTACCGTAACTTGAAAGTATTTCGATTTCTTGGCTTTATATAT
CTTGTGGAAAGGACGCATGCGATGACTGATCATGACCCTCGAGGTCGACGGTATCGATAAGCTCGCTTCACGAGAT
TCCAGCAGGTCGAGGGACCTAATAACTTCGTATAGCATACATTATACGAAGTTATATTAAGGGTTCCAAGCTTAAG
CGGCCGCGCCACCATGAGGATATTTGCTGTCTTTATATTCATGACCTACTGGCATTTGCTGAACGCATTTACTGTC
ACGGTTCCCAAGGACCTATATGTGGTAGAGTATGGTAGCAATATGACAATTGAATGCAAATTCCCAGTAGAAAAAC
AATTAGACCTGGCTGCACTAATTGTCTATTGGGAAATGGAGGATAAGAACATTATTCAATTTGTGCATGGAGAGGA
AGACCTGAAGGTTCAGCATAGTAGCTACAGACAGAGGGCCCGGCTGTTGAAGGACCAGCTCTCCCTGGGAAATGCT
GCACTTCAGATCACAGATGTGAAATTGCAGGATGCAGGGGTGTACCGCTGCATGATCAGCTATGGTGGTGCCGACT
ACAAGCGAATTACTGTGAAAGTCAATGCCCCATACAACAAAATCAACCAAAGAATTTTGGTTGTGGATCCAGTCAC
CTCTGAACATGAACTGACATGTCAGGCTGAGGGCTACCCCAAGGCCGAAGTCATCTGGACAAGCAGTGACCATCAA
GTCCTGAGTGGTAAGACCACCACCACCAATTCCAAGAGAGAGGAGAAGCTTTTCAATGTGACCAGCACACTGAGAA
TCAACACAACAACTAATGAGATTTTCTACTGCACTTTTAGGAGATTAGATCCTGAGGAAAACCATACAGCTGAATT
GGTCATCCCAGAACTACCTCTGGCACATCCTCCAAATGAAAGGACTCACTTGGTAATTCTGGGAGCCATCTTATTA
TGCCTTGGTGTAGCACTGACATTCATCTTCCGTTTAAGAAAAGGGAGAATGATGGATGTGAAAAAATGTGGCATCC
AAGATACAAACTCAAAGAAGCAAAGTGATACACATTTGGAGGAGACGTAACCTAGGGATGGATCCGACACTCTCTA
CTGATACTCATCACTAACGTTACTGACATAACGAAGTAGCTTGGAATAAGGCCGGTGTGCGTTTGTCTATATGTTA
TTTTCCACCATATTGCCGTCTTTTGGCAATGTGAGGCTGCATTAGTTATTAATAGTAATCAATTACGGGGTCATTA
GTTCATAGCCCATATATGGAGTTCCGCGTTACATAACTTACGGTAAATGGCCCGCCTGGCTGACCGCCCAACGACC
CCCGCCCATTGACGTCAATAATGACGTATGTTCCCATAGTAACGCCAATAGGGACTTTCCATTGACGTCAATGGGT
GGAGTATTTACGGTAAACTGCCCACTTGGCAGTACATCAAGTGTATCATATGCCAAGTACGCCCCCTATTGACGTC
AATGACGGTAAATGGCCCGCCTGGCATTATGCCCAGTACATGACCTTATGGGACTTTCCTACTTGGCAGTACATCT
ACGTATTAGTCATCGCTATTACCATGGTGATGCGGTTTTGGCAGTACATCAATGGGCGTGGATAGCGGTTTGACTC
ACGGGGATTTCCAAGTCTCCACCCCATTGACGTCAATGGGAGTTTGTTTTGGCACCAAAATCAACGGGACTTTCCA
AAATGTCGTAACAACTCCGCCCCATTGACGCAAATGGGCGGTAGGCGTGTACGGTGGGAGGTCTATATAAGCAGAG
CTGGTTTAGTGAACCGTCAGATCCGCTAGCATGTTCACACTCGAAGATTTCGTTGGGGACTGGCGACAGACAGCCG
GCTACAACCTGGACCAAGTCCTTGAACAGGGAGGTGTGTCCAGTTTGTTTCAGAATCTCGGGGTGTCCGTAACTCC
GATCCAAAGGATTGTCCTGAGCGGTGAAAATGGGCTGAAGATCGACATCCATGTCATCATCCCGTATGAAGGTCTG
AGCGGCGACCAAATGGGCCAGATCGAAAAAATTTTTAAGGTGGTGTACCCTGTGGATGATCATCACTTTAAGGTGA
TCCTGCACTATGGCACACTGGTAATCGACGGGGTTACGCCGAACATGATCGACTATTTCGGACGGCCGTATGAAGG
CATCGCCGTGTTCGACGGCAAAAAGATCACTGTAACAGGGACCCTGTGGAACGGCAACAAAATTATCGACGAGCGC
CTGATCAACCCCGACGGCTCCCTGCTGTTCCGAGTAACCATCAACGGAGTGACCGGCTGGCGGCTGTGCGAACGCA
TTCTGGCGGGAGCTGGCGCCGGAGCTAGATCTGTGAGCAAGGGCGAGGAGCTGTTCACCGGGGTGGTGCCCATCCT
GGTCGAGCTGGACGGCGACGTAAACGGCCACAAGTTCAGCGTGTCCGGCGAGGGCGAGGGCGATGCCACCTACGGC
AAGCTGACCCTGAAGTTCATCTGCACCACCGGCAAGCTGCCCGTGCCCTGGCCCACCCTCGTGACCACCCTGACCT
ACGGCGTGCAGTGCTTCAGCCGCTACCCCGACCACATGAAGCAGCACGACTTCTTCAAGTCCGCCATGCCCGAAGG
CTACGTCCAGGAGCGCACCATCTTCTTCAAGGACGACGGCAACTACAAGACCCGCGCCGAGGTGAAGTTCGAGGGC
GACACCCTGGTGAACCGCATCGAGCTGAAGGGCATCGACTTCAAGGAGGACGGCAACATCCTGGGGCACAAGCTGG
AGTACAACTACAACAGCCACAACGTCTATATCATGGCCGACAAGCAGAAGAACGGCATCAAGGTGAACTTCAAGAT
CCGCCACAACATCGAGGACGGCAGCGTGCAGCTCGCCGACCACTACCAGCAGAACACCCCCATCGGCGACGGCCCC
GTGCTGCTGCCCGACAACCACTACCTGAGCACCCAGTCCGCCCTGAGCAAAGACCCCAACGAGAAGCGCGATCACA
TGGTCCTGCTGGAGTTCGTGACCGCCGCCGGGATCACTCTCGGCATGGACGAGCTGTACGTCGACTGAGAATTCGT
CGAGGGACCTAATAACTTCGTATAGCATACATTATACGAAGTTATACATGTTTAAGGGTTCCGGTTCCACTAGGTA
CAATTCGATATCAAGCTTATCGATAATCAACCTCTGGATTACAAAATTTGTGAAAGATTGACTGGTATTCTTAACT
ATGTTGCTCCTTTTACGCTATGTGGATACGCTGCTTTAATGCCTTTGTATCATGCTATTGCTTCCCGTATGGCTTT

CATTTTCTCCTCCTTGTATAAATCCTGGTTGCTGTCTCTTTATGAGGAGTTGTGGCCCGTTGTCAGGCAACGTGGC
GTGGTGTGCACTGTGTTTGCTGACGCAACCCCCACTGGTTGGGGCATTGCCACCACCTGTCAGCTCCTTTCCGGGA
CTTTCGCTTTCCCCCTCCCTATTGCCACGGCGGAACTCATCGCCGCCTGCCTTGCCCGCTGCTGGACAGGGGCTCG
GCTGTTGGGCACTGACAATTCCGTGGTGTTGTCGGGGAAATCATCGTCCTTTCCTTGGCTGCTCGCCTGTGTTGCC
ACCTGGATTCTGCGCGGGACGTCCTTCTGCTACGTCCCTTCGGCCCTCAATCCAGCGGACCTTCCTTCCCGCGGCC
TGCTGCCGGCTCTGCGGCCTCTTCCGCGTCTTCGCCTTCGCCCTCAGACGAGTCGGATCTCCCTTTGGGCCGCCTC
CCCGCATCGATACCGTCGACCTCGATCGAGACCTAGAAAAACATGGAGCAATCACAAGTAGCAATACAGCAGCTAC
CAATGCTGATTGTGCCTGGCTAGAAGCACAAGAGGAGGAGGAGGTGGGTTTTCCAGTCACACCTCAGGTACCTTTA
AGACCAATGACTTACAAGGCAGCTGTAGATCTTAGCCACTTTTTAAAAGAAAAGGGGGGACTGGAAGGGCTAATTC
ACTCCCAACGAAGACAAGATATCCTTGATCTGTGGATCTACCACACACAAGGCTACTTCCCTGATTGGCAGAACTA
CACACCAGGGCCAGGGATCAGATATCCACTGACCTTTGGATGGTGCTACAAGCTAGTACCAGTTGAGCAAGAGAAG
GTAGAAGAAGCCAATGAAGGAGAGAACACCCGCTTGTTACACCCTGTGAGCCTGCATGGGATGGATGACCCGGAGA
GAGAAGTATTAGAGTGGAGGTTTGACAGCCGCCTAGCATTTCATCACATGGCCCGAGAGCTGCATCCGGACTGTAC
TGGGTCTCTCTGGTTAGACCAGATCTGAGCCTGGGAGCTCTCTGGCTAACTAGGGAACCCACTGCTTAAGCCTCAA
TAAAGCTTGCCTTGAGTGCTTCAAGTAGTGTGTGCCCGTCTGTTGTGTGACTCTGGTAACTAGAGATCCCTCAGAC
CCTTTTAGTCAGTGTGGAAAATCTCTAGCAGCATGTGAGCAAAAGGCCAGCAAAAGGCCAGGAACCGTAAAAAGGC
CGCGTTGCTGGCGTTTTTCCATAGGCTCCGCCCCCCTGACGAGCATCACAAAAATCGACGCTCAAGTCAGAGGTGG
CGAAACCCGACAGGACTATAAAGATACCAGGCGTTTCCCCCTGGAAGCTCCCTCGTGCGCTCTCCTGTTCCGACCC
TGCCGCTTACCGGATACCTGTCCGCCTTTCTCCCTTCGGGAAGCGTGGCGCTTTCTCATAGCTCACGCTGTAGGTA
TCTCAGTTCGGTGTAGGTCGTTCGCTCCAAGCTGGGCTGTGTGCACGAACCCCCCGTTCAGCCCGACCGCTGCGCC
TTATCCGGTAACTATCGTCTTGAGTCCAACCCGGTAAGACACGACTTATCGCCACTGGCAGCAGCCACTGGTAACA
GGATTAGCAGAGCGAGGTATGTAGGCGGTGCTACAGAGTTCTTGAAGTGGTGGCCTAACTACGGCTACACTAGAAG
AACAGTATTTGGTATCTGCGCTCTGCTGAAGCCAGTTACCTTCGGAAAAAGAGTTGGTAGCTCTTGATCCGGCAAA
CAAACCACCGCTGGTAGCGGTGGTTTTTTTGTTTGCAAGCAGCAGATTACGCGCAGAAAAAAAGGATCTCAAGAAG
ATCCTTTGATCTTTTCTACGGGGTCTGACGCTCAGTGGAACGAAAACTCACGTTAAGGGATTTTGGTCATGAGATT
ATCAAAAAGGATCTTCACCTAGATCCTTTTAAATTAAAAATGAAGTTTTAAATCAATCTAAAGTATATATGAGTAA
ACTTGGTCTGACAGTTACCAATGCTTAATCAGTGAGGCACCTATCTCAGCGATCTGTCTATTTCGTTCATCCATAG
TTGCCTGACTCCCCGTCGTGTAGATAACTACGATACGGGAGGGCTTACCATCTGGCCCCAGTGCTGCAATGATACC
GCGAGACCCACGCTCACCGGCTCCAGATTTATCAGCAATAAACCAGCCAGCCGGAAGGGCCGAGCGCAGAAGTGGT
CCTGCAACTTTATCCGCCTCCATCCAGTCTATTAATTGTTGCCGGGAAGCTAGAGTAAGTAGTTCGCCAGTTAATA
GTTTGCGCAACGTTGTTGCCATTGCTGCAGGCATCGTGGTGTCACGCTCGTCGTTTGGTATGGCTTCATTCAGCTC
CGGTTCCCAACGATCAAGGCGAGTTACATGATCCCCCATGTTGTGCAAAAAAGCGGTTAGCTCCTTCGGTCCTCCG
ATCGTTGTCAGAAGTAAGTTGGCCGCAGTGTTATCACTCATGGTTATGGCAGCACTGCATAATTCTCTTACTGTCA
TGCCATCCGTAAGATGCTTTTCTGTGACTGGTGAGTACTCAACCAAGTCATTCTGAGAATAGTGTATGCGGCGACC
GAGTTGCTCTTGCCCGGCGTCAATACGGGATAATACCGCGCCACATAGCAGAACTTTAAAAGTGCTCATCATTGGA
AAACGTTCTTCGGGGCGAAAACTCTCAAGGATCTTACCGCTGTTGAGATCCAGTTCGATGTAACCCACTCGTGCAC
CCAACTGATCTTCAGCATCTTTTACTTTCACCAGCGTTTCTGGGTGAGCAAAAACAGGAAGGCAAAATGCCGCAAA
AAAGGGAATAAGGGCGACACGGAAATGTTGAATACTCATACTCTTCCTTTTTCAATATTATTGAAGCATTTATCAG
GGTTATTGTCTCATGAGCGGATACATATTTGAATGTATTTAGAAAAATAAACAAATAGGGGTTCCGCGCACATTTC
CCCGAAAAGTGCCACCTGACGTCGACGGATCGGGAGATCTCCCGATCCCCTATGGTGCACTCTCAGTACAATCTGC
TCTGATGCCGCATAGTTAAGCCAGTATCTGCTCCCTGCTTGTGTGTTGGAGGTCGCTGAGTAGTGCGCGAGCAAAA
TTTAAGCTACAACAAGGCAAGGCTTGACCGACAATTGCATGAAGAATCTGCTTAGGGTTAGGCGTTTTGCGCTGCT
TCGCGATGTACGGGCCAGATATACGCGTTGACATTGATTATTGACTAGTTATTAATAGTAATCAATTACGGGGTCA
TTAGTTCATAGCCCATATATGGAGTTCCGCGTTACATAACTTACGGTAAATGGCCCGCCTGGCTGACCGCCCAACG

ACCCCCGCCCATTGACGTCAATAATGACGTATGTTCCCATAGTAACGCCAATAGGGACTTTCCATTGACGTCAATG
GGTGGAGTATTTACGGTAAACTGCCCACTTGGCAGTACATCAAGTGTATCATATGCCAAGTACGCCCCCTATTGAC
GTCAATGACGGTAAATGGCCCGCCTGGCATTATGCCCAGTACATGACCTTATGGGACTTTCCTACTTGGCAGTACA
TCTACGTATTAGTCATCGCTATTACCATGGTGATGCGGTTTTGGCAGTACATCAATGGGCGTGGATAGCGGTTTGA
CTCACGGGGATTTCCAAGTCTCCACCCCATTGACGTCAATGGGAGTTTGTTTTGGCACCAAAATCAACGGGACTTT
CCAAAATGTCGTAACAACTCCGCCCCATTGACGCAAATGGGCGGTAGGCGTGTACGGTGGGAGGTCTATATAAGCA
GCGCGTTTTGCCTGTACTGGGTCTCTCTGGTTAGACCAGATCTGAGCCTGGGAGCTCTCTGGCTAACTAGGGAACC
CACTGCTTAAGCCTCAATAAAGCTTGCCTTGAGTGCTTCAAGTAGTGTGTGCCCGTCTGTTGTGTGACTCTGGTAA
CTAGAGATCCCTCAGACCCTTTTAGTCAGTGTGGAAAATCTCTAGCAGTGGCGCCCGAACAGGGACTTGAAAGCGA
AAGGGAAACCAGAGGAGCTCTCTCGACGCAGGACTCGGCTTGCTGAAGCGCGCACGGCAAGAGGCGAGGGGCGGCG
ACTGGTGAGTACGCCAAAAATTTTGACTAGCGGAGGCTAGAAGGAGAGAGATGGGTGCGAGAGCGTCAGTATTAAG
CGGGGGAGAATTAGATCGCGATGGGAAAAAATTCGGTTAAGGCCAGGGGGAAAGAAAAAATATAAATTAAAACATAT
AGTATGGGCAAGCAGGGAGCTAGAACGATTCGCAGTTAATCCTGGCCTGTTAGAAACATCAGAAGGCTGTAGACAA
ATACTGGGACAGCTACAACCATCCCTTCAGACAGGATCAGAAGAACTTAGATCATTATATAATACAGTAGCAACCC
TCTATTGTGTGCATCAAAGGATAGAGATAAAAGACACCAAGGAAGCTTTAGACAAGATAGAGGAAGAGCAAAACAA
AAGTAAGACCACCGCACAGCAAGCGGCCGGCCGCTGATCTTCAGACCTGGAGGAGGAGATATGAGGGACAATTGGA
GAAGTGAATTATATAAATATAAAGTAGTAAAAATTGAACCATTAGGAGTAGCACCCACCAAGGCAAAGAGAAGAGT
GGTGCAGAGAGAAAAAAGAGCAGTGGGAATAGGAGCTTTGTTCCTTGGGTTCTTGGGAGCAGCAGGAAGCACTATG
GGCGCAGCGTCAATGACGCTGACGGTACAGGCCAGACAATTATTGTCTGGTATAGTGCAGCAGCAGAACAATTTGC
TGAGGGCTATTGAGGCGCAACAGCATCTGTTGCAACTCACAGTCTGGGGCATCAAGCAGCTCCAGGCAAGAATCCT
GGCTGTGGAAAGATACCTAAAGGATCAACAGCTCCTGGGGATTTGGGGTTGCTCTGGAAAACTCATTTGCACCACT
GCTGTGCCTTGGAATGCTAGTTGGAGTAATAAATCTCTGGAACAGATTTGGAATCACACGACCTGGATGGAGTGGG
ACAGAGAAATTAACAATTACACAAGCTTAATACACTCCTTAATTGAAGAATCGCAAAACCAGCAAGAAAAGAATGA
ACAAGAATTATTGGAATTAGATAAATGGGCAAGTTTGTGGAATTGGTTTAACATAACAAATTGGCTGTGGTATATA
AAATTATTCATAATGATAGTAGGAGGCTTGGTAGGTTTAAGAATAGTTTTTGCTGTACTTTCTATAGTGAATAGAG
TTAGGCAGGGATATTCACCATTATCGTTTCAGACCCACCTCCCAACCCCGAGGGGACCCGACAGGCCCGAAGGAAT
AGAAGAAGAAGGTGGAGAGAGAGACAGAGACAGATCCATTCGATTAGTGAACGGATCGGCACTGCGTGCGCCAATT
CTGCAGACAAATGGCAGTATTCATCCACAATTTTAAAAGAAAAGGGGGGATTGGGGGGTACAGTGCAGGGGAAAGA
ATAGTAGACATAATAGCAACAGACATACAAACTAAAGAATTACAAAAACAAATTACAAAAATTCAAAATTTTCGGG
TTTATTACAGGGACAGCAGAGATCCAGTTTGGTTAGTACCGGGCCCGCTCTAGA

(2) Lenti-pEF1α-Cas12a-P2A-mCherry: Backbone plasmid for the construction of Cas12a-expressing lentivirus vectors, modified from the following plasmid: P08-pEF1α-spacer -P2A-mCherry.

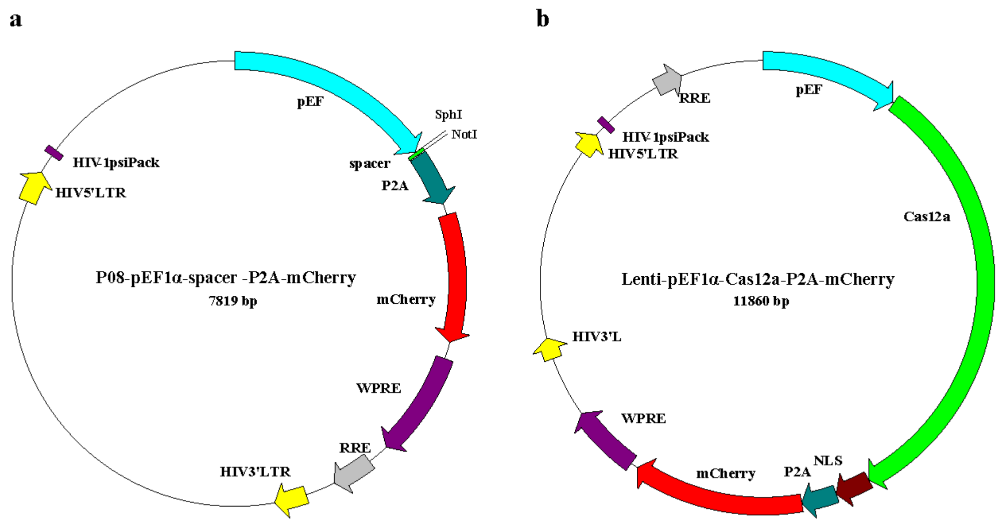

1) 5 end adaptor of the Cas12a coding region

- **Kozak sequence**
- **N-terminal SV40 NLS**

ACCGGTGCCACCATGGCCCCAAAGAAGAAGCGGAAGGTC

2) 3 end adaptor of the Cas12a coding region (without stop codon)

- **C-terminal NLS: Nucleoplasmin NLS (bipartite nuclear localization signal from nucleoplasmin)**
- **3×HA tag**

AAACGTCCGGCGGCCACGAAAAAGCCGGCCAGGCAAAAAAGAAAAAAGGCTCCTACCCGTACGATGTTCCGGATT
ACGCGTATCCGTACGACGTGCCGGATTATGCATACCCGTATGATGTCCCGGACTATGCC

The vector backbone sequences between restriction sites EcoRI and XbaI are the same using P08-pU6-PDL1-pCMV-EGFP. Here, we show the different parts used to express Cas12a.

- EF1α promoter
- SphI restriction site
- 20bp spacer: between two adjacent restriction sites to avoid steric hindrance impairing digestion efficiency in a double digestion reaction
- NotI restriction site
- P2A linker
- mCherry

GCCCGTCAGTGGGCAGAGCGCACATCGCCCACAGTCCCCGAGAAGTTGGGGGGAGGGGTCGGCAATTGAACCGGT
GCCTAGAGAAGGTGGCGCGGGGTAAACTGGGAAAGTGATGTCGTGTACTGGCTCCGCCTTTTTCCCGAGGGTGGG
GGAGAACCGTATATAAGTGCAGTAGTCGCCGTGAACGTTCTTTTTCGCAACGGGTTTGCCGCCAGAACACAGGTA
AGTGCCGTGTGTGGTTCCCGCGGGCCTGGCCTCTTTACGGGTTATGGCCCTTGCGTGCCTTGAATTACTTCCACC
TGGCTGCAGTACGTGATTCTTGATCCCGAGCTTCGGGTTGGAAGTGGGTGGGAGAGTTCGAGGCCTTGCGCTTAA
GGAGCCCCTTCGCCTCGTGCTTGAGTTGAGGCCTGGCCTGGGCGCTGGGGCCGCCGCGTGCGAATCTGGTGGCAC

CTTCGCGCCTGTCTCGCTGCTTTCGATAAGTCTCTAGCCATTTAAAATTTTTGATGACCTGCTGCGACGCTTTTT
TTCTGGCAAGATAGTCTTGTAAATGCGGGCCAAGATCTGCACACTGGTATTTCGGTTTTTGGGGCCGCGGGCGGC
GACGGGGCCCGTGCGTCCCAGCGCACATGTTCGGCGAGGCGGGGCCTGCGAGCGCGGCCACCGAGAATCGGACGG
GGGTAGTCTCAAGCTGGCCGGCCTGCTCTGGTGCCTGGCCTCGCGCCGCCGTGTATCGCCCCGCCCTGGGCGGCA
AGGCTGGCCCGGTCGGCACCAGTTGCGTGAGCGGAAAGATGGCCGCTTCCCGGCCCTGCTGCAGGGAGCTCAAAA
TGGAGGACGCGGCGCTCGGGAGAGCGGGCGGGTGAGTCACCCACACAAAGGAAAAGGGCCTTTCCGTCCTCAGCC
GTCGCTTCATGTGACTCCACGGAGTACCGGGCGCCGTCCAGGCACCTCGATTAGTTCTCGAGCTTTTGGAGTACG
TCGTCTTTAGGTTGGGGGGAGGGGTTTTATGCGATGGAGTTTCCCCACACTGAGTGGGTGGAGACTGAAGTTAGG
CCAGCTTGGCACTTGATGTAATTCTCCTTGGAATTTGCCCTTTTTGAGTTTGGATCTTGGTTCATTCTCAAGCCT
CAGACAGTGGTTCAAAGTTTTTTTCTTCCATTTCAGGTGTCGTGAGCATGCGATGACTGATCATGAGCGGCCGCG
GTAGCGGAGCTACTAACTTCAGCCTGCTGAAGCAGGCTGGAGACGTGGAGGAGAACCCTGGACCTATGGTGAGCA
AGGGCGAGGAGGATAACTCCGCCATCATCAAGGAGTTCCTGCGCTTCAAGGTGCACATGGAGGGCTCCGTGAACG
GCCACGAGTTCGAGATCGAGGGCGAGGGCGAGGGCCGCCCCTACGAGGGCACCCAGACCGCCAAGCTGAAGGTGA
CCAAGGGTGGCCCCCTGCCCTTCGCCTGGGACATCCTGTCCCCTCAGTTCATGTACGGCTCCAAGGCCTACGTGA
AGCACCCCGCCGACATCCCCGACTACTTGAAGCTGTCCTTCCCCGAGGGCTTCAAGTGGGAGCGCGTGATGAACT
TCGAGGACGGCGGCGTGGTGACCGTGACCCAGGACTCCTCTCTGCAGGACGGCGAGTTCATCTACAAGGTGAAGC
TGCGCGGCACCAACTTCCCCTCCGACGGCCCCGTAATGCAGAAGAAGACCATGGGCTGGGAGGCCTCCTCCGAGC
GGATGTACCCCGAGGACGGCGCCCTGAAGGGCGAGATCAAGCAGAGGCTGAAGCTGAAGGACGGCGGCCACTACG
ACGCTGAGGTCAAGACCACCTACAAGGCCAAGAAGCCCGTGCAGCTGCCCGGCGCCTACAACGTCAACATCAAGT
TGGACATCACCTCCCACAACGAGGACTACACCATCGTGGAACAGTACGAACGCGCCGAGGGCCGCCACTCCACCG
GCGGCATGGACGAGCTGTACAAGTGGTAA

Humanized Coding Sequences of 20 Cas12a Effectors

The coding region sequences of 16 Cas12a orthologues and 4 Cas12a variants used in this study, corresponding to P1–P20 in Supplementary Table S2, are presented here.

The full coding regions of 16 Cas12a orthologues were synthesized by BGI Geneland Scientific (China). When synthesizing gene fragments, universal adaptors were added on either side of the coding region for amplification and to provide homologous arms for cloning into the expressing vector. The N-terminal adaptor contains a partial EF1α promoter, a kozak sequence, and SV40 NLS, while the C-terminal adaptor includes a C-terminal NLS and the 3×HA sequence.

N-terminal adaptor:

CAAAGTTTTTTTCTTCCATTTCAGGTGTCGTGAGCATGCACCGGTGCCACCATGGCCCCAAAGAAGAAGCGGAAG
GTC

C-terminal adaptor:

AAACGTCCGGCGGCCACGAAAAAAGCCGGCCAGGCAAAAAAGAAAAAAGGCTCCTACCCGTACGATGTTCCGGAT
TACGCGTATCCGTACGACGTGCCGGATTATGCATACCCGTATGATGTCCCGGACTATGCC

The coding region sequences of 16 Cas12a orthologues without stop codons are listed as follows, with their corresponding GenBank accession numbers:

Cas12a orthologue	GenBank accession number
AsCas12a	OK557997
LbCas12a	OK557998
FnCas12a	OK557999
MbCas12a	OK558000
BsCas12a	OK558001
HkCas12a	OK558002
ArCas12a	OK558003
PrCas12a	OK558004
PxCas12a	OK558005
Mb3Cas12a	OK557996
PdCas12a	OK558006
PiCas12a	OK558007
Lb2Cas12a	OK558008
ErCas12a	OK558009
EeCas12a	OK558010
CeCas12a	OK558011

>AaCas12a

ATGACCCAGTTCGAGGGCTTTACCAACCTGTATCAGGTGAGCAAAACCCTGCGTTTTGAGCTGATCCCGCAGGGC
AAAACCCTGAAACATATCCAGGAGCAGGGCTTCATCGAGGAGGACAAAGCCCGCAATGATCATTACAAAGAGCTG
AAACCGATCATCGATCGTATCTACAAAACCTATGCCGACCAGTGCCTGCAGCTGGTGCAGCTGGATTGGGAGAAC
CTGAGCGCCGCCATCGACTCCTATCGCAAAGAGAAAACCGAGGAGACCCGTAACGCCCTGATCGAGGAGCAGGCC
ACCTATCGCAATGCCATCCATGACTACTTCATCGGCCGTACCGACAACCTGACCGATGCCATCAATAAACGCCAT
GCCGAGATCTACAAAGGCCTGTTCAAAGCCGAGCTGTTTAATGGCAAAGTGCTGAAACAGCTGGGCACCGTGACC
ACCACCGAGCATGAGAACGCCCTGCTGCGTAGCTTCGACAAATTTACCACCTACTTCTCCGGCTTTTATGAGAAC
CGTAAAAACGTGTTCAGCGCCGAGGATATCAGCACCGCCATCCCGCATCGCATCGTGCAGGACAACTTCCCGAAA
TTTAAAGAGAATTGTCATATCTTCACCCGCCTGATCACCGCCGTGCCGAGCCTGCGTGAGCATTTTGAGAACGTG
AAAAAAGCCATCGGCATCTTCGTGAGCACCTCCATCGAGGAGGTGTTTTCCTTCCCGTTTTATAACCAGCTGCTG
ACCCAGACCCAGATCGACCTGTATAACCAGCTGCTGGGCGGCATCTCTCGTGAGGCAGGCACCGAGAAAATCAAA
GGCCTGAACGAGGTGCTGAATCTGGCCATCCAGAAAAATGATGAGACCGCCCATATCATCGCCTCCCTGCCGCAT
CGCTTCATCCCGCTGTTTAAACAGATCCTGTCCGATCGTAACACCCTGTCTTTCATCCTGGAGGAGTTTAAAAGC
GACGAGGAAGTGATCCAGTCCTTCTGCAAATACAAAACCCTGCTGCGCAACGAGAACGTGCTGGAGACCGCCGAG
GCCCTGTTTAACGAGCTGAACAGCATCGACCTGACCCATATCTTCATCAGCCATAAAAAACTGGAGACCATCAGC
AGCGCCCTGTGCGACCATTGGGATACCCTGCGTAATGCCCTGTATGAGCGTCGCATCTCCGAGCTGACCGGCAAA
ATCACCAAATCTGCCAAAGAGAAAGTGCAGCGCAGCCTGAAACATGAGGATATCAACCTGCAGGAGATCATCTCT
GCCGCAGGCAAAGAGCTGAGCGAGGCCTTCAAACAGAAAACCAGCGAGATCCTGTCCCATGCACATGCCGCCCTG
GATCAGCCGCTGCCGACCACCCTGAAAAAACAGGAGGAGAAAGAGATCCTGAAATCTCAGCTGGACAGCCTGCTG
GGCCTGTACCATCTGCTGGACTGGTTTGCCGTGGATGAGTCCAACGAGGTGGACCCGGAGTTCTCTGCCCGTCTG

ACCGGCATCAAACTGGAGATGGAGCCGTCTCTGAGCTTCTACAACAAAGCCCGCAATTATGCCACCAAAAAACCG
TACTCCGTGGAGAAATTCAAACTGAACTTTCAGATGCCGACCCTGGCCTCTGGCTGGGACGTGAATAAAGAGAAA
AACAATGGCGCCATCCTGTTTGTGAAAAACGGCCTGTACTATCTGGGCATCATGCCGAAACAGAAAGGCCGTTAT
AAAGCCCTGAGCTTCGAGCCGACCGAGAAAACCAGCGAGGGCTTTGATAAAATGTACTATGACTACTTCCCGGAT
GCCGCCAAAATGATCCCGAAATGCAGCACCCAGCTGAAAGCCGTGACCGCCCATTTTCAGACCCATACCACCCCG
ATCCTGCTGTCCAACAATTTCATCGAGCCGCTGGAGATCACCAAAGAGATCTACGACCTGAACAATCCGGAGAAA
GAGCCGAAAAAATTTCAGACCGCCTACGCCAAAAAAACCGGCGACCAGAAAGGCTACCGCGAGGCCCTGTGCAAA
TGGATCGACTTCACCCGTGATTTTCTGTCCAAATATACCAAAACCACCTCTATCGATCTGTCTAGCCTGCGTCCG
TCCTCTCAGTATAAAGACCTGGGCGAGTACTATGCCGAGCTGAATCCGCTGCTGTACCATATCAGCTTCCAGCGC
ATCGCCGAGAAAGAGATCATGGATGCCGTGGAGACCGGCAAACTGTACCTGTTCCAGATCTATAACAAAGACTTT
GCCAAAGGCCATCATGGCAAACCGAATCTGCATACCCTGTATTGGACCGGCCTGTTTTCTCCGGAGAACCTGGCC
AAAACCAGCATCAAACTGAATGGCCAGGCCGAGCTGTTCTACCGCCCGAAATCCCGTATGAAACGTATGGCACAT
CGTCTGGGCGAGAAAATGCTGAACAAAAAACTGAAAGATCAGAAAACCCCGATCCCGGACACCCTGTACCAGGAG
CTGTACGACTATGTGAATCATCGCCTGTCCCATGACCTGTCTGATGAGGCCCGTGCCCTGCTGCCGAACGTGATC
ACCAAAGAGGTGTCTCATGAGATCATCAAAGATCGTCGCTTTACCAGCGACAAATTCTTTTTCCATGTGCCGATC
ACCCTGAACTATCAGGCCGCCAATTCCCCGTCTAAATTCAACCAGCGTGTGAATGCCTACCTGAAAGAGCATCCG
GAGACCCCGATCATCGGCATCGATCGTGGCGAGCGCAACCTGATCTATATCACCGTGATCGACTCCACCGGCAAA
ATCCTGGAGCAGCGTAGCCTGAACACCATCCAGCAGTTTGATTACCAGAAAAAACTGGACAACCGTGAGAAAGAG
CGTGTGGCAGCACGTCAGGCCTGGTCTGTGGTGGGCACCATCAAAGATCTGAAACAGGGCTATCTGAGCCAGGTC
ATCCATGAGATCGTGGACCTGATGATCCATTACCAGGCCGTGGTGGTGCTGGAGAACCTGAATTTCGGCTTTAAA
AGCAAACGTACCGGCATCGCCGAGAAAGCCGTGTACCAGCAGTTCGAGAAAATGCTGATCGATAAACTGAATTGC
CTGGTGCTGAAAGACTATCCGGCAGAGAAAGTGGGCGGCGTGCTGAACCCGTACCAGCTGACCGACCAGTTCACC
TCCTTTGCCAAAATGGGCACCCAGTCTGGCTTCCTGTTTTACGTGCCGGCCCCGTATACCTCTAAAATCGATCCG
CTGACCGGCTTCGTGGACCCGTTCGTGTGGAAAACCATCAAAAATCATGAGAGCCGCAAACATTTCCTGGAGGGC
TTCGACTTTCTGCATTACGACGTGAAAACCGGCGACTTCATCCTGCATTTTAAAATGAACCGCAATCTGTCCTTC
CAGCGTGGCCTGCCGGGCTTTATGCCGGCATGGGATATCGTGTTCGAGAAAAACGAGACCCAGTTTGACGCCAAA
GGCACCCCGTTCATCGCCGGCAAACGCATCGTGCCGGTGATCGAGAATCATCGCTTCACCGGCCGCTACCGTGAC
CTGTATCCGGCCAACGAGCTGATCGCCCTGCTGGAGGAGAAAGGCATCGTGTTCCGTGATGGCTCCAACATCCTG
CCGAAACTGCTGGAGAATGACGATTCTCATGCCATCGACACCATGGTGGCCCTGATCCGCAGCGTGCTGCAGATG
CGTAACTCCAATGCCGCCACCGGCGAGGACTATATCAACAGCCCGGTGCGCGATCTGAATGGCGTGTGCTTCGAC
TCCCGTTTTCAGAACCCGGAGTGGCCGATGGACGCCGATGCCAATGGCGCCTACCATATCGCCCTGAAAGGCCAG
CTGCTGCTGAATCATCTGAAAGAGAGCAAAGATCTGAAACTGCAGAACGGCATCTCCAATCAGGACTGGCTGGCC
TACATCCAGGAGCTGCGCAAC

>LbCas12a

ATGAGCAAGCTGGAGAAGTTTACAAACTGCTACTCCCTGTCTAAGACCCTGAGGTTCAAGGCCATCCCTGTGGGC
AAGACCCAGGAGAACATCGACAATAAGCGGCTGCTGGTGGAGGACGAGAAGAGAGCCGAGGATTATAAGGGCGTG
AAGAAGCTGCTGGATCGCTACTATCTGTCTTTTATCAACGACGTGCTGCACAGCATCAAGCTGAAGAATCTGAAC
AATTACATCAGCCTGTTCCGGAAGAAAACCAGAACCGAGAAGGAGAATAAGGAGCTGGAGAACCTGGAGATCAAT
CTGCGGAAGGAGATCGCCAAGGCCTTCAAGGGCAACGAGGGCTACAAGTCCCTGTTTAAGAAGGATATCATCGAG
ACAATCCTGCCAGAGTTCCTGGACGATAAGGACGAGATCGCCCTGGTGAACAGCTTCAATGGCTTTACCACAGCC
TTCACCGGCTTCTTTGATAACAGAGAGAATATGTTTTCCGAGGAGGCCAAGAGCACATCCATCGCCTTCAGGTGT
ATCAACGAGAATCTGACCCGCTACATCTCTAATATGGACATCTTCGAGAAGGTGGACGCCATCTTTGATAAGCAC

GAGGTGCAGGAGATCAAGGAGAAGATCCTGAACAGCGACTATGATGTGGAGGATTTCTTTGAGGGCGAGTTCTTT
AACTTTGTGCTGACACAGGAGGGCATCGACGTGTATAACGCCATCATCGGCGGCTTCGTGACCGAGAGCGGCGAG
AAGATCAAGGGCCTGAACGAGTACATCAACCTGTATAATCAGAAAACCAAGCAGAAGCTGCCTAAGTTTAAGCCA
CTGTATAAGCAGGTGCTGAGCGATCGGGAGTCTCTGAGCTTCTACGGCGAGGGCTATACATCCGATGAGGAGGTG
CTGGAGGTGTTTAGAAACACCCTGAACAAGAACAGCGAGATCTTCAGCTCCATCAAGAAGCTGGAGAAGCTGTTC
AAGAATTTTGACGAGTACTCTAGCGCCGGCATCTTTGTGAAGAACGGCCCCGCCATCAGCACAATCTCCAAGGAT
ATCTTCGGCGAGTGGAACGTGATCCGGGACAAGTGGAATGCCGAGTATGACGATATCCACCTGAAGAAGAAGGCC
GTGGTGACCGAGAAGTACGAGGACGATCGGAGAAAGTCCTTCAAGAAGATCGGCTCCTTTTCTCTGGAGCAGCTG
CAGGAGTACGCCGACGCCGATCTGTCTGTGGTGGAGAAGCTGAAGGAGATCATCATCCAGAAGGTGGATGAGATC
TACAAGGTGTATGGCTCCTCTGAGAAGCTGTTCGACGCCGATTTTGTGCTGGAGAAGAGCCTGAAGAAGAACGAC
GCCGTGGTGGCCATCATGAAGGACCTGCTGGATTCTGTGAAGAGCTTCGAGAATTACATCAAGGCCTTCTTTGGC
GAGGGCAAGGAGACAAACAGGGACGAGTCCTTCTATGGCGATTTTGTGCTGGCCTACGACATCCTGCTGAAGGTG
GACCACATCTACGATGCCATCCGCAATTATGTGACCCAGAAGCCCTACTCTAAGGATAAGTTCAAGCTGTATTTT
CAGAACCCTCAGTTCATGGGCGGCTGGGACAAGGATAAGGAGACAGACTATCGGGCCACCATCCTGAGATACGGC
TCCAAGTACTATCTGGCCATCATGGATAAGAAGTACGCCAAGTGCCTGCAGAAGATCGACAAGGACGATGTGAAC
GGCAATTACGAGAAGATCAACTATAAGCTGCTGCCCGGCCCTAATAAGATGCTGCCAAAGGTGTTCTTTTCTAAG
AAGTGGATGGCCTACTATAACCCCAGCGAGGACATCCAGAAGATCTACAAGAATGGCACATTCAAGAAGGGCGAT
ATGTTTAACCTGAATGACTGTCACAAGCTGATCGACTTCTTTAAGGATAGCATCTCCCGGTATCCAAAGTGGTCC
AATGCCTACGATTTCAACTTTTCTGAGACAGAGAAGTATAAGGACATCGCCGGCTTTTACAGAGAGGTGGAGGAG
CAGGGCTATAAGGTGAGCTTCGAGTCTGCCAGCAAGAAGGAGGTGGATAAGCTGGTGGAGGAGGGCAAGCTGTAT
ATGTTCCAGATCTATAACAAGGACTTTTCCGATAAGTCTCACGGCACACCCAATCTGCACACCATGTACTTCAAG
CTGCTGTTTGACGAGAACAATCACGGACAGATCAGGCTGAGCGGAGGAGCAGAGCTGTTCATGAGGCGCGCCTCC
CTGAAGAAGGAGGAGCTGGTGGTGCACCCAGCCAACTCCCCTATCGCCAACAAGAATCCAGATAATCCCAAGAAA
ACCACAACCCTGTCCTACGACGTGTATAAGGATAAGAGGTTTTCTGAGGACCAGTACGAGCTGCACATCCCAATC
GCCATCAATAAGTGCCCCAAGAACATCTTCAAGATCAATACAGAGGTGCGCGTGCTGCTGAAGCACGACGATAAC
CCCTATGTGATCGGCATCGATAGGGGCGAGCGCAATCTGCTGTATATCGTGGTGGTGGACGGCAAGGGCAACATC
GTGGAGCAGTATTCCCTGAACGAGATCATCAACAACTTCAACGGCATCAGGATCAAGACAGATTACCACTCTCTG
CTGGACAAGAAGGAGAAGGAGAGGTTCGAGGCCCGCCAGAACTGGACCTCCATCGAGAATATCAAGGAGCTGAAG
GCCGGCTATATCTCTCAGGTGGTGCACAAGATCTGCGAGCTGGTGGAGAAGTACGATGCCGTGATCGCCCTGGAG
GACCTGAACTCTGGCTTTAAGAATAGCCGCGTGAAGGTGGAGAAGCAGGTGTATCAGAAGTTCGAGAAGATGCTG
ATCGATAAGCTGAACTACATGGTGGACAAGAAGTCTAATCCTTGTGCAACAGGCGGCGCCCTGAAGGGCTATCAG
ATCACCAATAAGTTCGAGAGCTTTAAGTCCATGTCTACCCAGAACGGCTTCATCTTTTACATCCCTGCCTGGCTG
ACATCCAAGATCGATCCATCTACCGGCTTTGTGAACCTGCTGAAAACCAAGTATACCAGCATCGCCGATTCCAAG
AAGTTCATCAGCTCCTTTGACAGGATCATGTACGTGCCCGAGGAGGATCTGTTCGAGTTTGCCCTGGACTATAAG
AACTTCTCTCGCACAGACGCCGATTACATCAAGAAGTGGAAGCTGTACTCCTACGGCAACCGGATCAGAATCTTC
CGGAATCCTAAGAAGAACAACGTGTTCGACTGGGAGGAGGTGTGCCTGACCAGCGCCTATAAGGAGCTGTTCAAC
AAGTACGGCATCAATTATCAGCAGGGCGATATCAGAGCCCTGCTGTGCGAGCAGTCCGACAAGGCCTTCTACTCT
AGCTTTATGGCCCTGATGAGCCTGATGCTGCAGATGCGGAACAGCATCACAGGCCGCACCGACGTGGATTTTCTG
ATCAGCCCTGTGAAGAACTCCGACGGCATCTTCTACGATAGCCGGAACTATGAGGCCCAGGAGAATGCCATCCTG
CCAAAGAACGCCGACGCCAATGGCGCCTATAACATCGCCAGAAAGGTGCTGTGGGCCATCGGCCAGTTCAAGAAG
GCCGAGGACGAGAAGCTGGATAAGGTGAAGATCGCCATCTCTAACAAGGAGTGGCTGGAGTACGCCCAGACCAGC
GTGAAGCAC

>FnCas12a

ATGAGCAAGCTGGAGAAGTTTACAAACTGCTACTCCCTGTCTAAGACCCTGAGGTTCAAGGCCATCCCTGTGGGC
AAGACCCAGGAGAACATCGACAATAAGCGGCTGCTGGTGGAGGACGAGAAGAGAGCCGAGGATTATAAGGGCGTG
AAGAAGCTGCTGGATCGCTACTATCTGTCTTTTATCAACGACGTGCTGCACAGCATCAAGCTGAAGAATCTGAAC
AATTACATCAGCCTGTTCCGGAAGAAAACCAGAACCGAGAAGGAGAATAAGGAGCTGGAGAACCTGGAGATCAAT
CTGCGGAAGGAGATCGCCAAGGCCTTCAAGGGCAACGAGGGCTACAAGTCCCTGTTTAAGAAGGATATCATCGAG
ACAATCCTGCCAGAGTTCCTGGACGATAAGGACGAGATCGCCCTGGTGAACAGCTTCAATGGCTTTACCACAGCC
TTCACCGGCTTCTTTGATAACAGAGAGAATATGTTTTCCGAGGAGGCCAAGAGCACATCCATCGCCTTCAGGTGT
ATCAACGAGAATCTGACCCGCTACATCTCTAATATGGACATCTTCGAGAAGGTGGACGCCATCTTTGATAAGCAC
GAGGTGCAGGAGATCAAGGAGAAGATCCTGAACAGCGACTATGATGTGGAGGATTTCTTTGAGGGCGAGTTCTTT
AACTTTGTGCTGACACAGGAGGGCATCGACGTGTATAACGCCATCATCGGCGGCTTCGTGACCGAGAGCGGCGAG
AAGATCAAGGGCCTGAACGAGTACATCAACCTGTATAATCAGAAAACCAAGCAGAAGCTGCCTAAGTTTAAGCCA
CTGTATAAGCAGGTGCTGAGCGATCGGGAGTCTCTGAGCTTCTACGGCGAGGGCTATACATCCGATGAGGAGGTG
CTGGAGGTGTTTAGAAACACCCTGAACAAGAACAGCGAGATCTTCAGCTCCATCAAGAAGCTGGAGAAGCTGTTC
AAGAATTTTGACGAGTACTCTAGCGCCGGCATCTTTGTGAAGAACGGCCCCGCCATCAGCACAATCTCCAAGGAT
ATCTTCGGCGAGTGGAACGTGATCCGGGACAAGTGGAATGCCGAGTATGACGATATCCACCTGAAGAAGAAGGCC
GTGGTGACCGAGAAGTACGAGGACGATCGGAGAAAGTCCTTCAAGAAGATCGGCTCCTTTTCTCTGGAGCAGCTG
CAGGAGTACGCCGACGCCGATCTGTCTGTGGTGGAGAAGCTGAAGGAGATCATCATCCAGAAGGTGGATGAGATC
TACAAGGTGTATGGCTCCTCTGAGAAGCTGTTCGACGCCGATTTTGTGCTGGAGAAGAGCCTGAAGAAGAACGAC
GCCGTGGTGGCCATCATGAAGGACCTGCTGGATTCTGTGAAGAGCTTCGAGAATTACATCAAGGCCTTCTTTGGC
GAGGGCAAGGAGACAAACAGGGACGAGTCCTTCTATGGCGATTTTGTGCTGGCCTACGACATCCTGCTGAAGGTG
GACCACATCTACGATGCCATCCGCAATTATGTGACCCAGAAGCCCTACTCTAAGGATAAGTTCAAGCTGTATTTT
CAGAACCCTCAGTTCATGGGCGGCTGGGACAAGGATAAGGAGACAGACTATCGGGCCACCATCCTGAGATACGGC
TCCAAGTACTATCTGGCCATCATGGATAAGAAGTACGCCAAGTGCCTGCAGAAGATCGACAAGGACGATGTGAAC
GGCAATTACGAGAAGATCAACTATAAGCTGCTGCCCGGCCCTAATAAGATGCTGCCAAAGGTGTTCTTTTCTAAG
AAGTGGATGGCCTACTATAACCCCAGCGAGGACATCCAGAAGATCTACAAGAATGGCACATTCAAGAAGGGCGAT
ATGTTTAACCTGAATGACTGTCACAAGCTGATCGACTTCTTTAAGGATAGCATCTCCCGGTATCCAAAGTGGTCC
AATGCCTACGATTTCAACTTTTCTGAGACAGAGAAGTATAAGGACATCGCCGGCTTTTACAGAGAGGTGGAGGAG
CAGGGCTATAAGGTGAGCTTCGAGTCTGCCAGCAAGAAGGAGGTGGATAAGCTGGTGGAGGAGGGCAAGCTGTAT
ATGTTCCAGATCTATAACAAGGACTTTTCCGATAAGTCTCACGGCACACCCAATCTGCACACCATGTACTTCAAG
CTGCTGTTTGACGAGAACAATCACGGACAGATCAGGCTGAGCGGAGGAGCAGAGCTGTTCATGAGGCGCGCCTCC
CTGAAGAAGGAGGAGCTGGTGGTGCACCCAGCCAACTCCCCTATCGCCAACAAGAATCCAGATAATCCCAAGAAA
ACCACAACCCTGTCCTACGACGTGTATAAGGATAAGAGGTTTTCTGAGGACCAGTACGAGCTGCACATCCCAATC
GCCATCAATAAGTGCCCCAAGAACATCTTCAAGATCAATACAGAGGTGCGCGTGCTGCTGAAGCACGACGATAAC
CCCTATGTGATCGGCATCGATAGGGGCGAGCGCAATCTGCTGTATATCGTGGTGGTGGACGGCAAGGGCAACATC
GTGGAGCAGTATTCCCTGAACGAGATCATCAACAACTTCAACGGCATCAGGATCAAGACAGATTACCACTCTCTG
CTGGACAAGAAGGAGAAGGAGAGGTTCGAGGCCCGCCAGAACTGGACCTCCATCGAGAATATCAAGGAGCTGAAG
GCCGGCTATATCTCTCAGGTGGTGCACAAGATCTGCGAGCTGGTGGAGAAGTACGATGCCGTGATCGCCCTGGAG
GACCTGAACTCTGGCTTTAAGAATAGCCGCGTGAAGGTGGAGAAGCAGGTGTATCAGAAGTTCGAGAAGATGCTG
ATCGATAAGCTGAACTACATGGTGGACAAGAAGTCTAATCCTTGTGCAACAGGCGGCGCCCTGAAGGGCTATCAG
ATCACCAATAAGTTCGAGAGCTTTAAGTCCATGTCTACCCAGAACGGCTTCATCTTTTACATCCCTGCCTGGCTG
ACATCCAAGATCGATCCATCTACCGGCTTTGTGAACCTGCTGAAAACCAAGTATACCAGCATCGCCGATTCCAAG
AAGTTCATCAGCTCCTTTGACAGGATCATGTACGTGCCCGAGGAGGATCTGTTCGAGTTTGCCCTGGACTATAAG

AACTTCTCTCGCACAGACGCCGATTACATCAAGAAGTGGAAGCTGTACTCCTACGGCAACCGGATCAGAATCTTC
CGGAATCCTAAGAAGAACAACGTGTTCGACTGGGAGGAGGTGTGCCTGACCAGCGCCTATAAGGAGCTGTTCAAC
AAGTACGGCATCAATTATCAGCAGGGCGATATCAGAGCCCTGCTGTGCGAGCAGTCCGACAAGGCCTTCTACTCT
AGCTTTATGGCCCTGATGAGCCTGATGCTGCAGATGCGGAACAGCATCACAGGCCGCACCGACGTGGATTTTCTG
ATCAGCCCTGTGAAGAACTCCGACGGCATCTTCTACGATAGCCGGAACTATGAGGCCCAGGAGAATGCCATCCTG
CCAAAGAACGCCGACGCCAATGGCGCCTATAACATCGCCAGAAAGGTGCTGTGGGCCATCGGCCAGTTCAAGAAG
GCCGAGGACGAGAAGCTGGATAAGGTGAAGATCGCCATCTCTAACAAGGAGTGGCTGGAGTACGCCCAGACCAGC
GTGAAGCAC

>MbCas12a

ATGCTGTTCCAGGACTTTACCCACCTGTATCCACTGTCCAAGACAGTGAGATTTGAGCTGAAGCCCATCGATAGG
ACCCTGGAGCACATCCACGCCAAGAACTTCCTGTCTCAGGACGAGACAATGGCCGATATGCACCAGAAGGTGAAA
GTGATCCTGGACGATTACCACCGCGACTTCATCGCCGATATGATGGGCGAGGTGAAGCTGACCAAGCTGGCCGAG
TTCTATGACGTGTACCTGAAGTTTCGGAAGAACCCAAAGGACGATGAGCTGCAGAAGCAGCTGAAGGATCTGCAG
GCCGTGCTGAGAAAGGAGATCGTGAAGCCCATCGGCAATGGCGGCAAGTATAAGGCCGGCTACGACAGGCTGTTC
GGCGCCAAGCTGTTTAAGGACGGCAAGGAGCTGGGCGATCTGGCCAAGTTCGTGATCGCACAGGAGGGAGAGAGC
TCCCCAAAGCTGGCCCACCTGGCCCACTTCGAGAAGTTTTCCACCTATTTCACAGGCTTTCACGATAACCGGAAG
AATATGTATTCTGACGAGGATAAGCACACCGCCATCGCCTACCGCCTGATCCACGAGAACCTGCCCCGGTTTATC
GACAATCTGCAGATCCTGACCACAATCAAGCAGAAGCACTCTGCCCTGTACGATCAGATCATCAACGAGCTGACC
GCCAGCGGCCTGGACGTGTCTCTGGCCAGCCACCTGGATGGCTATCACAAGCTGCTGACACAGGAGGGCATCACC
GCCTACAATACACTGCTGGGAGGAATCTCCGGAGAGGCAGGCTCTCCTAAGATCCAGGGCATCAACGAGCTGATC
AATTCTCACCACAACCAGCACTGCCACAAGAGCGAGAGAATCGCCAAGCTGAGGCCACTGCACAAGCAGATCCTG
TCCGACGGCATGAGCGTGTCCTTCCTGCCCTCTAAGTTTGCCGACGATAGCGAGATGTGCCAGGCCGTGAACGAG
TTCTATCGCCACTACGCCGACGTGTTCGCCAAGGTGCAGAGCCTGTTCGACGGCTTTGACGATCACCAGAAGGAT
GGCATCTACGTGGAGCACAAGAACCTGAATGAGCTGTCCAAGCAGGCCTTCGGCGACTTTGCACTGCTGGGACGC
GTGCTGGACGGATACTATGTGGATGTGGTGAATCCAGAGTTCAACGAGCGGTTTGCCAAGGCCAAGACCGACAAT
GCCAAGGCCAAGCTGACAAAGGAGAAGGATAAGTTCATCAAGGGCGTGCACTCCCTGGCCTCTCTGGAGCAGGCC
ATCGAGCACTATACCGCAAGGCACGACGATGAGAGCGTGCAGGCAGGCAAGCTGGGACAGTACTTCAAGCACGGC
CTGGCCGGAGTGGACAACCCCATCCAGAAGATCCACAACAATCACAGCACCATCAAGGGCTTTCTGGAGAGGGAG
CGCCCTGCAGGAGAGAGAGCCCTGCCAAAGATCAAGTCCGGCAAGAATCCTGAGATGACACAGCTGAGGCAGCTG
AAGGAGCTGCTGGATAACGCCCTGAATGTGGCCCACTTCGCCAAGCTGCTGACCACAAAGACCACACTGGACAAT
CAGGATGGCAACTTCTATGGCGAGTTTGGCGTGCTGTACGACGAGCTGGCCAAGATCCCCACCCTGTATAACAAG
GTGAGAGATTACCTGAGCCAGAAGCCTTTCTCCACCGAGAAGTACAAGCTGAACTTTGGCAATCCAACACTGCTG
AATGGCTGGGACCTGAACAAGGAGAAGGATAATTTCGGCGTGATCCTGCAGAAGGACGGCTGCTACTATCTGGCC
CTGCTGGACAAGGCCCACAAGAAGGTGTTTGATAACGCCCCTAATACAGGCAAGAGCATCTATCAGAAGATGATC
TATAAGTACCTGGAGGTGAGGAAGCAGTTCCCCAAGGTGTTCTTTTCCAAGGAGGCCATCGCCATCAACTACCAC
CCTTCTAAGGAGCTGGTGGAGATCAAGGACAAGGGCCGGCAGAGATCCGACGATGAGCGCCTGAAGCTGTATCGG
TTTATCCTGGAGTGTCTGAAGATCCACCCTAAGTACGATAAGAAGTTCGAGGGCGCCATCGGCGACATCCAGCTG
TTTAAGAAGGATAAGAAGGGCAGAGAGGTGCCAATCAGCGAGAAGGACCTGTTCGATAAGATCAACGGCATCTTT
TCTAGCAAGCCTAAGCTGGAGATGGAGGACTTCTTTATCGGCGAGTTCAAGAGGTATAACCCAAGCCAGGACCTG
GTGGATCAGTATAATATCTACAAGAAGATCGACTCCAACGATAATCGCAAGAAGGAGAATTTCTACAACAATCAC
CCCAAGTTTAAGAAGGATCTGGTGCGGTACTATTACGAGTCTATGTGCAAGCACGAGGAGTGGGAGGAGAGCTTC
GAGTTTTCCAAGAAGCTGCAGGACATCGGCTGTTACGTGGATGTGAACGAGCTGTTTACCGAGATCGAGACACGG

AGACTGAATTATAAGATCTCCTTCTGCAACATCAATGCCGACTACATCGATGAGCTGGTGGAGCAGGGCCAGCTG
TATCTGTTCCAGATCTACAACAAGGACTTTTCCCCAAAGGCCCACGGCAAGCCCAATCTGCACACCCTGTACTTC
AAGGCCCTGTTTTCTGAGGACAACCTGGCCGATCCTATCTATAAGCTGAATGGCGAGGCCCAGATCTTCTACAGA
AAGGCCTCCCTGGACATGAACGAGACAACAATCCACAGGGCCGGCGAGGTGCTGGAGAACAAGAATCCCGATAAT
CCTAAGAAGAGACAGTTCGTGTACGACATCATCAAGGATAAGAGGTACACACAGGACAAGTTCATGCTGCACGTG
CCAATCACCATGAACTTTGGCGTGCAGGGCATGACAATCAAGGAGTTCAATAAGAAGGTGAACCAGTCTATCCAG
CAGTATGACGAGGTGAACGTGATCGGCATCGATCGGGGCGAGAGACACCTGCTGTACCTGACCGTGATCAATAGC
AAGGGCGAGATCCTGGAGCAGTGTTCCCTGAACGACATCACCACAGCCTCTGCCAATGGCACACAGATGACCACA
CCTTACCACAAGATCCTGGATAAGAGGGAGATCGAGCGCCTGAACGCCCGGGTGGGATGGGGCGAGATCGAGACA
ATCAAGGAGCTGAAGTCTGGCTATCTGAGCCACGTGGTGCACCAGATCAGCCAGCTGATGCTGAAGTACAACGCC
ATCGTGGTGCTGGAGGACCTGAATTTCGGCTTTAAGAGGGGCCGCTTTAAGGTGGAGAAGCAGATCTATCAGAAC
TTCGAGAATGCCCTGATCAAGAAGCTGAACCACCTGGTGCTGAAGGACAAGGCCGACGATGAGATCGGCTCTTAC
AAGAATGCCCTGCAGCTGACCAACAATTTCACAGATCTGAAGAGCATCGGCAAGCAGACCGGCTTCCTGTTTTAT
GTGCCCGCCTGGAACACCTCTAAGATCGACCCTGAGACAGGCTTTGTGGATCTGCTGAAGCCAAGATACGAGAAC
ATCGCCCAGAGCCAGGCCTTCTTTGGCAAGTTCGACAAGATCTGCTATAATGCCGACAAGGATTACTTCGAGTTT
CACATCGACTACGCCAAGTTTACCGATAAGGCCAAGAATAGCCGCCAGATCTGGACAATCTGTTCCCACGGCGAC
AAGCGGTACGTGTACGATAAGACAGCCAACCAGAATAAGGGCGCCGCCAAGGGCATCAACGTGAATGATGAGCTG
AAGTCCCTGTTCGCCCGCCACCACATCAACGAGAAGCAGCCCAACCTGGTCATGGACATCTGCCAGAACAATGAT
AAGGAGTTTCACAAGTCTCTGATGTACCTGCTGAAAACCCTGCTGGCCCTGCGGTACAGCAACGCCTCCTCTGAC
GAGGATTTCATCCTGTCCCCCGTGGCAAACGACGAGGGCGTGTTCTTTAATAGCGCCCTGGCCGACGATACACAG
CCTCAGAATGCCGATGCCAACGGCGCCTACCACATCGCCCTGAAGGGCCTGTGGCTGCTGAATGAGCTGAAGAAC
TCCGACGATCTGAACAAGGTGAAGCTGGCCATCGACAATCAGACCTGGCTGAATTTCGCCCAGAACAGG

>BsCas12a

ATGTACTACCAGAACCTGACCAAGAAGTACCCCGTGAGCAAGACCATCCGCAACGAGCTGATCCCCATCGGCAAG
ACCCTGGAGAACATCCGCAAGAACAACATCCTGGAGAGCGACGTGAAGCGCAAGCAGGACTACGAGCACGTGAAG
GGCATCATGGACGAGTACCACAAGCAGCTGATCAACGAGGCCCTGGACAACTACATGCTGCCCAGCCTGAACCAG
GCCGCCGAGATCTACCTGAAGAAGCACGTGGACGTGGAGGACCGCGAGGAGTTCAAGAAGACCCAGGACCTGCTG
CGCCGCGAGGTGACCGGCCGCCTGAAGGAGCACGAGAACTACACCAAGATCGGCAAGAAGGACATCCTGGACCTG
CTGGAGAAGCTGCCCAGCATCAGCGAGGAGGACTACAACGCCCTGGAGAGCTTCCGCAACTTCTACACCTACTTC
ACCAGCTACAACAAGGTGCGCGAGAACCTGTACAGCGACGAGGAGAAGAGCAGCACCGTGGCCTACCGCCTGATC
AACGAGAACCTGCCCAAGTTCCTGGACAACATCAAGAGCTACGCCTTCGTGAAGGCCGCCGGCGTGCTGGCCGAC
TGCATCGAGGAGGAGGAGCAGGACGCCCTGTTCATGGTGGAGACCTTCAACATGACCCTGACCCAGGAGGGCATC
GACATGTACAACTACCAGATCGGCAAGGTGAACAGCGCCATCAACCTGTACAACCAGAAGAACCACAAGGTGGAG
GAGTTCAAGAAGATCCCCAAGATGAAGGTGCTGTACAAGCAGATCCTGAGCGACCGCGAGGAGGTGTTCATCGGC
GAGTTCAAGGACGACGAGACCCTGCTGAGCAGCATCGGCGCCTACGGCAACGTGCTGATGACCTACCTGAAGAGC
GAGAAGATCAACATCTTCTTCGACGCCCTGCGCGAGAGCGAGGGCAAGAACGTGTACGTGAAGAACGACCTGAGC
AAGACCACCATGAGCAACATCGTGTTCGGCAGCTGGAGCGCCTTCGACGAGCTGCTGAACCAGGAGTACGACCTG
GCCAACGAGAACAAGAAGAAGGACGACAAGTACTTCGAGAAGCGCCAGAAGGAGCTGAAGAAGAACAAGAGCTAC
ACCCTGGAGCAGATGAGCAACCTGAGCAAGGAGGACATCAGCCCCATCGAGAACTACATCGAGCGCATCAGCGAG
GACATCGAGAAGATCTGCATCTACAACGGCGAGTTCGAGAAGATCGTGGTGAACGAGCACGACAGCAGCCGCAAG
CTGAGCAAGAACATCAAGGCCGTGAAGGTGATCAAGGACTACCTGGACAGCATCAAGGAGCTGGAGCACGACATC
AAGCTGATCAACGGCAGCGGCCAGGAGCTGGAGAAGAACCTGGTGGTGTACGTGGGCCAGGAGGAGGCCCTGGAG

CAGCTGCGCCCCGTGGACAGCCTGTACAACCTGACCCGCAACTACCTGACCAAGAAGCCCTTCAGCACCGAGAAG
GTGAAGCTGAACTTCAACAAGAGCACCCTGCTGAACGGCTGGGACAAGAACAAGGAGACCGACAACCTGGGCATC
CTGTTCTTCAAGGACGGCAAGTACTACCTGGGCATCATGAACACCACCGCCAACAAGGCCTTCGTGAACCCCCCC
GCCGCCAAGACCGAGAACGTGTTCAAGAAGGTGGACTACAAGCTGCTGCCCGGCAGCAACAAGATGCTGCCCAAG
GTGTTCTTCGCCAAGAGCAACATCGGCTACTACAACCCCAGCACCGAGCTGTACAGCAACTACAAGAAGGGCACC
CACAAGAAgggcccCAGCTTCAGCATCGACGACTGCCACAACCTGATCGACTTCTTCAAGGAGAGCATCAAGAAG
CACGAGGACTGGAGCAAGTTCGGCTTCGAGTTCAGCGACACCGCCGACTACCGCGACATCAGCGAGTTCTACCGC
GAGGTGGAGAAGCAGGGCTACAAGCTGACCTTCACCGACATCGACGAGAGCTACATCAACGACCTGATCGAGAAG
AACGAGCTGTACCTGTTCCAGATCTACAACAAGGACTTCAGCGAGTACAGCAAGGGCAAGCTGAACCTGCACACC
CTGTACTTCATGATGCTGTTCGACCAGCGCAACCTGGACAACGTGGTGTACAAGCTGAACGGCGAGGCCGAGGTG
TTCTACCGCCCCGCCAGCATCGCCGAGAACGAGCTGGTGATCCACAAGGCCGGCGAGGGCATCAAGAACAAGAAC
CCCAACCGCGCCAAGGTGAAGGAGACCAGCACCTTCAGCTACGACATCGTGAAGGACAAGCGCTACAGCAAGTAC
AAGTTCACCCTGCACATCCCCATCACCATGAACTTCGGCGTGGACGAGGTGCGCCGCTTCAACGACGTGATCAAC
AACGCCCTGCGCACCGACGACAACGTGAACGTGATCGGCATCGACCGCGGCGAGCGCAACCTGCTGTACGTGGTG
GTGATCAACAGCGAGGGCAAGATCCTGGAGCAGATCAGCCTGAACAGCATCATCAACAAGGAGTACGACATCGAG
ACCAACTACCACGCCCTGCTGGACGAGCGCGAGGACGACCGCAACAAGGCCCGCAAGGACTGGAACACCATCGAG
AACATCAAGGAGCTGAAGACCGGCTACCTGAGCCAGGTGGTGAACGTGGTGGCCAAGCTGGTGCTGAAGTACAAC
GCCATCATCTGCCTGGAGGACCTGAACTTCGGCTTCAAGCGCGGCCGCCAGAAGGTGGAGAAGCAGGTGTACCAG
AAGTTCGAGAAGATGCTGATCGAGAAGCTGAACTACCTGGTGATCGACAAGAGCCGCGAGCAGGTGAGCCCCGAG
AAGATGGGCGGCGCCCTGAACGCCCTGCAGCTGACCAGCAAGTTCAAGAGCTTCGCCGAGCTGGGCAAGCAGAGC
GGCATCATCTACTACGTGCCCGCCTACCTGACCAGCAAGATCGACCCCACCACCGGCTTCGTGAACCTGTTCTAC
ATCAAGTACGAGAACATCGAGAAGGCCAAGCAGTTCTTCGACGGCTTCGACTTCATCCGCTTCAACAAGAAGGAC
GACATGTTCGAGTTCAGCTTCGACTACAAGAGCTTCACCCAGAAGGCCTGCGGCATCCGCAGCAAGTGGATCGTG
TACACCAACGGCGAGCGCATCATCAAGTACCCCAACCCCGAGAAGAACAACCTGTTCGACGAGAAGGTGATCAAC
GTGACCGACGAGATCAAGGGCCTGTTCAAGCAGTACCGCATCCCCTACGAGAACGGCGAGGACATCAAGGAGATC
ATCATCAGCAAGGCCGAGGCCGACTTCTACAAGCGCCTGTTCCGCCTGCTGCACCAGACCCTGCAGATGCGCAAC
AGCACCAGCGACGGCACCCGCGACTACATCATCAGCCCCGTGAAGAACGACCGCGGCGAGTTCTTCTGCAGCGAG
TTCAGCGAGGGCACCATGCCCAAGGACGCCGACGCCAACGGCGCCTACAACATCGCCCGCAAGGGCCTGTGGGTG
CTGGAGCAGATCCGCCAGAAGGACGAGGGCGAGAAGGTGAACCTGAGCATGACCAACGCCGAGTGGCTGAAGTAC
GCCCAGCTGCACCTGCTG

>HkCas12a

ATGTTCGAGAAGCTGAGCAACATCGTGAGCATCAGCAAGACCATCCGCTTCAAGCTGATCCCCGTGGGCAAGACC
CTGGAGAACATCGAGAAGCTGGGCAAGCTGGAGAAGGACTTCGAGCGCAGCGACTTCTACCCCATCCTGAAGAAC
ATCAGCGACGACTACTACCGCCAGTACATCAAGGAGAAGCTGAGCGACCTGAACCTGGACTGGCAGAAGCTGTAC
GACGCCCACGAGCTGCTGGACAGCAGCAAGAAGGAGAGCCAGAAGAACCTGGAGATGATCCAGGCCCAGTACCGC
AAGGTGCTGTTCAACATCCTGAGCGGCGAGCTGGACAAGAGCGGCGAGAAGAACAGCAAGGACCTGATCAAGAAC
AACAAGGCCCTGTACGGCAAGCTGTTCAAGAAGCAGTTCATCCTGGAGGTGCTGCCCGACTTCGTGAACAACAAC
GACAGCTACAGCGAGGAGGACCTGGAGGGCCTGAACCTGTACAGCAAGTTCACCACCCGCCTGAAGAACTTCTGG
GAGACCCGCAAGAACGTGTTCACCGACAAGGACATCGTGACCGCCATCCCCTTCCGCGCCGTGAACGAGAACTTC
GGCTTCTACTACGACAACATCAAGATCTTCAACAAGAACATCGAGTACCTGGAGAACAAGATCCCCAACCTGGAG
AACGAGCTGAAGGAGGCCGACATCCTGGACGACAACCGCAGCGTGAAGGACTACTTCACCCCCAACGGCTTCAAC
TACGTGATCACCCAGGACGGCATCGACGTGTACCAGGCCATCCGCGGCGGCTTCACCAAGGAGAACGGCGAGAAG

GTGCAGGGCATCAACGAGATCCTGAACCTGACCCAGCAGCAGCTGCGCCGCAAGCCCGAGACCAAGAACGTGAAG
CTGGGCGTGCTGACCAAGCTGCGCAAGCAGATCCTGGAGTACAGCGAGAGCACCAGCTTCCTGATCGACCAGATC
GAGGACGACAACGACCTGGTGGACCGCATCAACAAGTTCAACGTGAGCTTCTTCGAGAGCACCGAGGTGAGCCCC
AGCCTGTTCGAGCAGATCGAGCGCCTGTACAACGCCCTGAAGAGCATCAAGAAGGAGGAGGTGTACATCGACGCC
CGCAACACCCAGAAGTTCAGCCAGATGCTGTTCGGCCAGTGGGACGTGATCCGCCGCGGCTACACCGTGAAGATC
ACCGAGGGCAGCAAGGAGGAGAAGAAGAAGTACAAGGAGTACCTGGAGCTGGACGAGACCAGCAAGGCCAAGCGC
TACCTGAACATCCGCGAGATCGAGGAGCTGGTGAACCTGGTGGAGGGCTTCGAGGAGGTGGACGTGTTCAGCGTG
CTGCTGGAGAAGTTCAAGATGAACAACATCGAGCGCAGCGAGTTCGAGGCCCCCATCTACGGCAGCCCCATCAAG
CTGGAGGCCATCAAGGAGTACCTGGAGAAGCACCTGGAGGAGTACCACAAGTGGAAGCTGCTGCTGATCGGCAAC
GACGACCTGGACACCGACGAGACCTTCTACCCCCTGCTGAACGAGGTGATCAGCGACTACTACATCATCCCCCTG
TACAACCTGACCCGCAACTACCTGACCCGCAAGCACAGCGACAAGGACAAGATCAAGGTGAACTTCGACTTCCCC
ACCCTGGCCGACGGCTGGAGCGAGAGCAAGATCAGCGACAACCGCAGCATCATCCTGCGCAAGGGCGGCTACTAC
TACCTGGGCATCCTGATCGACAACAAGCTGCTGATCAACAAGAAGAACAAGAGCAAGAAGATCTACGAGATCCTG
ATCTACAACCAGATCCCCGAGTTCAGCAAGAGCATCCCCAACTACCCCTTCACCAAGAAGGTGAAGGAGCACTTC
AAGAACAACGTGAGCGACTTCCAGCTGATCGACGGCTACGTGAGCCCCCTGATCATCACCAAGGAGATCTACGAC
ATCAAGAAGGAGAAGAAGTACAAGAAGGACTTCTACAAGGACAACAACACCAACAAGAACTACCTGTACACCATC
TACAAGTGGATCGAGTTCTGCAAGCAGTTCCTGTACAAGTACAAgggcccCAACAAGGAGAGCTACAAGGAGATG
TACGACTTCAGCACCCTGAAGGACACCAGCCTGTACGTGAACCTGAACGACTTCTACGCCGACGTGAACAGCTGC
GCCTACCGCGTGCTGTTCAACAAGATCGACGAGAACACCATCGACAACGCCGTGGAGGACGGCAAGCTGCTGCTG
TTCCAGATCTACAACAAGGACTTCAGCCCCGAGAGCAAGGGCAAGAAGAACCTGCACACCCTGTACTGGCTGAGC
ATGTTCAGCGAGGAGAACCTGCGCACCCGCAAGCTGAAGCTGAACGGCCAGGCCGAGATCTTCTACCGCAAGAAG
CTGGAGAAGAAGCCCATCATCCACAAGGAGGGCAGCATCCTGCTGAACAAGATCGACAAGGAGGGCAACACCATC
CCCGAGAACATCTACCACGAGTGCTACCGCTACCTGAACAAGAAGATCGGCCGCGAGGACCTGAGCGACGAGGCC
ATCGCCCTGTTCAACAAGGACGTGCTGAAGTACAAGGAGGCCCGCTTCGACATCATCAAGGACCGCCGCTACAGC
GAGAGCCAGTTCTTCTTCCACGTGCCCATCACCTTCAACTGGGACATCAAGACCAACAAGAACGTGAACCAGATC
GTGCAGGGCATGATCAAGGACGGCGAGATCAAGCACATCATCGGCATCGACCGCGGCGAGCGCCACCTGCTGTAC
TACAGCGTGATCGACCTGGAGGGCAACATCGTGGAGCAGGGCAGCCTGAACACCCTGGAGCAGAACCGCTTCGAC
AACAGCACCGTGAAGGTGGACTACCAGAACAAGCTGCGCACCCGCGAGGAGGACCGCGACCGCGCCCGCAAGAAC
TGGACCAACATCAACAAGATCAAGGAGCTGAAGGACGGCTACCTGAGCCACGTGGTGCACAAGCTGAGCCGCCTG
ATCATCAAGTACGAGGCCATCGTGATCATGGAGAACCTGAACCAGGGCTTCAAGCGCGGCCGCTTCAAGGTGGAG
CGCCAGGTGTACCAGAAGTTCGAGCTGGCCCTGATGAACAAGCTGAGCGCCCTGAGCTTCAAGGAGAAGTACGAC
GAGCGCAAGAACCTGGAGCCCAGCGGCATCCTGAACCCCATCCAGGCCTGCTACCCCGTGGACGCCTACCAGGAG
CTGCAGGGCCAGAACGGCATCGTGTTCTACCTGCCCGCCGCCTACACCAGCGTGATCGACCCCGTGACCGGCTTC
ACCAACCTGTTCCGCCTGAAGAGCATCAACAGCAGCAAGTACGAGGAGTTCATCAAGAAGTTCAAGAACATCTAC
TTCGACAACGAGGAGGAGGACTTCAAGTTCATCTTCAACTACAAGGACTTCGCCAAGGCCAACCTGGTGATCCTG
AACAACATCAAGAGCAAGGACTGGAAGATCAGCACCCGCGGCGAGCGCATCAGCTACAACAGCAAGAAGAAGGAG
TACTTCTACGTGCAGCCCACCGAGTTCCTGATCAACAAGCTGAAGGAGCTGAACATCGACTACGAGAACATCGAC
ATCATCCCCCTGATCGACAACCTGGAGGAGAAGGCCAAGCGCAAGATCCTGAAGGCCCTGTTCGACACCTTCAAG
TACAGCGTGCAGCTGCGCAACTACGACTTCGAGAACGACTACATCATCAGCCCCACCGCCGACGACAACGGCAAC
TACTACAACAGCAACGAGATCGACATCGACAAGACCAACCTGCCCAACAACGGCGACGCCAACGGCGCCTTCAAC
ATCGCCCGCAAGGGCCTGCTGCTGAAGGACCGCATCGTGAACAGCAACGAGAGCAAGGTGGACCTGAAGATCAAG
AACGAGGACTGGATCAACTTCATCATCAGC

>ArCas12a

atgcgcgatcacAACAACGGCACAAACAATTTCCAGAACTTCATTGGCATTTCATCACTCCAGAAGACCCTGAGG
AATGCCCTGACACCTACCGAGACAACCCAGCAATTCATTGTGAAGAACGGAATCATTAAGGAGGACGAACTGAGA
GGCGAGAACCGCCAGATCCTGAAAGACATCATGGACGACTACTATCGCGGTTTCATCTCCGAGACACTCAGCAGC
ATTGATGATATTGATTGGACATCTCTGTTTGAGAAGATGGAAATCCAGCTGAAGAATGGCGACAACAAGGACACT
CTCATTAAGGAGCAAGCAGAGAAGAGAAAGGCTATCTACAAGAAGTTTGCAGACGACGATCGCTTCAAGAATATG
TTTAGCGCAAAGCTGATAAGTGATATTCTCCCAGAGTTTGTGATTCACAACAACAACTATTCTGCCAGCGAGAAG
GAAGAGAAGACACAGGTCATCAAGCTCTTCTCTCGGTTTGCCACCTCATTCAAAGATTACTTCAAGAATCGGGCA
AATTGTTTCTCTGCCGATGACATCTCATCTTCCTCCTGTCACAGAATCGTTAATGATAACGCCGAAATCTTCTTC
TCAAATGCCCTGGTGTACCGGAGAATCGTGAAGAATCTGAGTAACGACGACATCAATAAGATTAGCGGTGATATG
AAGGACTCTCTGAAGAAGATGTCCCTGGAGAAGATATACAGCTATGAGAAGTACGGCGAGTTCATCACTCAAGAG
GGAATTAGCTTCTACAACGACATTTGTGGCAAGGTTAACTCATTCATGAATCTCTACTGCCAGAAGAACAAGGAG
AATAAGAATCTGTACAAACTGCGCAAACTGCATAAGCAAATCCTGTGTATCGCCGACACCTCTTATGAGGTGCCC
TACAAGTTTGAGTCTGACGAAGAGGTGTACCAGTCCGTGAACGGATTCCTGGACAACATCAGTTCTAAACACATC
GTCGAGCGGCTGAGGAAGATCGGCGACAACTACAATGGCTATAACCTGGACAAGATATACATCGTCAGTAAGTTC
TATGAAAGTGTGAGTCAGAAGACCTACCGGGACTGGGAGACTATCAACACCGCACTCGAAATCCACTACAATAAC
ATCCTGCCTGGCAACGGTAAGAGCAAGGCCGACAAGGTGAAGAAGGCCGTCAAGAACGACCTCCAGAAGAGCATC
ACCGAGATTAACGAACTGGTGAGTAACTACAAGCTCTGCCCAGACGATAACATTAAGGCAGAAACCTACATTCAT
GAGATTTCCCATATACTGAACAACTTTGAAGCTCAAGAGCTGAAATACAATCCCGAGATTCATCTGGTGGAGAGC
GAGCTGAAAGCATCCGAGCTGAAGAACGTTCTCGACGTCATCATGAACGCCTTCCACTGGTGTAGCGTGTTCATG
ACTGAGGAGCTGGTCGATAAAGACAACAATTTCTACGCCGAGCTGGAAGAAATCTACGACGAGATATACCCTGTG
ATTAGCCTCTACAATCTGGTCCGGAACTATGTGACCCAGAAGCCCTATTCAACTAAGAAGATCAAGCTGAACTTT
GGGATTCCTACCCTGGCCGACGGCTGGTCCAAGAGCAAAGAGTATTCCAATAACGCAATCATCCTGATGAGAGAC
AACCTGTATTACCTCGGTATCTTTAACGCTAAGAATAAGCCCGAGAAGAAGATCATCGAAGGAAATACATCCGAG
AACAAGGGCGACTACAAGAAGATGATCTATAACCTGCTGCCTGGCCCAAACAAGATGATCCCTAAGGTGTTCCTG
AGCAGCAAGACCGGAGTCGAGACTTACAAGCCAAGTGCCTACATACTGGAGGGCTATAAGCAGAACAAGCACCTG
AAATCTAGCAAAGATTTCGACATCACTTTCTGTCGCGACCTGATCGACTATTTCAAGAATTGTATTGCCATCCAC
CCAGAGTGGAAGAATTTCGGATTCGACTTCTCTGACACCTCCACATACGAGGACATCAGTGGCTTCTATAGAGAA
GTGGAGCTGCAAGGTTACAAGATCGACTGGACCTACATATCTGAGAAAGACATCGACCTGCTGCAAGAGAAAGGG
CAGCTCTACCTCTTCCAAATCTACAACAAGGACTTTAGTAAGAAGTCTACAGGTAATGACAATCTGCACACTATG
TACCTGAAGAATCTCTTCTCTGAAGAGAACCTCAAAGACGTGGTGCTGAAACTGAACGGCGAAGCAGAAATCTTC
TTTCGCAAATCATCCATTAAGAATCCTATCATACACAAGAAGGGTAGTATCCTGGTGAACAGGACATACGAAGCC
GAGGAGAAAGATCAGTTCGGCAACATTCAGATTGTGCGCAAGACTATTCCCGAGAATATCTACCAGGAGCTGTAC
AAATACTTCAACGATAAGTCTGATAAAGAGCTGTCAGACGAAGCAGCCAAGCTGAAGAATGCTGTGGGACATCAT
GAAGCAGCTACTAACATCGTGAAAGACTATAGATACACATACGACAAGTATTTCCTGCACATGCCAATTACCATC
AACTTCAAAGCCAATAAGACTTCTTTCATTAACGACCGCATCCTCCAGTACATTGCAAAGGAGAAAGACCTGCAC
GTGATCGGGATTGATCGCGGAGAACGGAACCTCATCTACGTTTCAGTCATCGACACATGCGGTAACATCGTCGAA
CAGAAGAGCTTCAACATTGTTAATGGGTATGATTACCAGATCAAACTCAAGCAACAGGAAGGCGCACGGCAGATT
GCTCGCAAGGAGTGGAAGGAAATTGGCAAGATCAAGGAAATCAAGGAAGGATACCTCAGCCTCGTCATTCATGAA
ATCAGCAAGATGGTGATCAAGTATAACGCAATCATCGCTATGGAGGACCTGAGTTATGGCTTCAAGAAAGGCAGA
TTCAAGGTGGAGCGGCAAGTCTACCAGAAATTCGAAACAATGCTGATCAACAAGCTGAACTACCTGGTGTTCAAA
GACATCAGCATAACCGAGAACGGAGGACTCCTGAAAGGCTACCAGCTCACATACATCCCAGAGAAACTCAAGAAT
GTGGGCCACCAATGCGGCTGCATCTTCTACGTCCCTGCCGCTTACACCAGCAAGATAGATCCCACTACAGGATTC

GTGAACATATTCAAATTCAAGGATCTGACAGTGGACGCCAAGAGGGAGTTCATCAAGAAGTTTGATAGTATTCGC

TATGACAGCGATAAGAATCTGTTCTGTTTCACCTTTGACTACAACAACTTCATTACCCAGAATACCGTTATGTCC

AAGTCTAGCTGGAGTGTGTATACCTACGGTGTTCGGATCAAGCGGAGGTTTGTCAATGGTAGATTCTCAAACGAA

AGCGACACCATCGACATCACAAAGGACATGGAGAAGACACTGGAAATGACTGACATAAACTGGAGAGATGGACAC

GACCTGCGGCAAGACATCATTGACTACGAGATCGTTCAGCACATCTTTGAAATCTTCAAGCTGACTGTTCAGATG

CGGAATAGTCTGAGCGAGCTGGAGGACCGGAATTACGACCGCCTGATCTCACCAGTCCTGAACGAGAATAACATC

TTCTACGATTCTGCCAAAGCAGGAGATGCCCTGCCAAAGGACGCTGATGCAAACGGTGCCTACTGCATCGCCCTC

AAAGGTCTGTACGAAATCAAGCAGATTACCGAGAATTGGAAGGAGGACGGGAAGTTCAGCAGAGACAAGCTCAAG

ATCAGCAATAAGGACTGGTTCGATTTCATTCAGAACAAGCGCTACCTG

>PrCas12a

ATGATCATCGGCCGCGACTTCAACATGTACTACCAGAACCTGACCAAGATGTACCCCATCAGCAAGACCCTGCGC

AACGAGCTGATCCCCGTGGGCAAGACCCTGGAGAACATCCGCAAGAACGGCATCCTGGAGGCCGACATCCAGCGC

AAGGCCGACTACGAGCACGTGAAGAAGCTGATGGACAACTACCACAAGCAGCTGATCAACGAGGCCCTGCAGGGC

GTGCACCTGAGCGACCTGAGCGACGCCTACGACCTGTACTTCAACCTGAGCAAGGAGAAGAACAGCGTGGACGCC

TTCAGCAAGTGCCAGGACAAGCTGCGCAAGGAGATCGTGAGCCTGCTGAAGAACCACGAGAACTTCCCCAAGATC

GGCAACAAGGAGATCATCAAGCTGCTGCAGAGCCTGTACGACAACGACACCGACTACAAGGCCCTGGACAGCTTC

AGCAACTTCTACACCTACTTCAGCAGCTACAACGAGGTGCGCAAGAACCTGTACAGCGACGAGGAGAAGAGCAGC

ACCGTGGCCTACCGCCTGATCAACGAGAACCTGCCCAAGTTCCTGGACAACATCAAGGCCTACGCCATCGCCAAG

AAGGCCGGCGTGCGCGCCGAGGGCCTGAGCGAGGAGGACCAGGACTGCCTGTTCATCATCGAGACCTTCGAGCGC

ACCCTGACCCAGGACGGCATCGACAACTACAACGCCGCCATCGGCAAGCTGAACACCGCCATCAACCTGTTCAAC

CAGCAGAACAAGAAGCAGGAGGGCTTCCGCAAGGTGCCCCAGATGAAGTGCCTGTACAAGCAGATCCTGAGCGAC

CGCGAGGAGGCCTTCATCGACGAGTTCAGCGACGACGAGGACCTGATCACCAACATCGAGAGCTTCGCCGAGAAC

ATGAACGTGTTCCTGAACAGCGAGATCATCACCGACTTCAAGATCGCCCTGGTGGAGAGCGACGGCAGCCTGGTG

TACATCAAGAACGACGTGAGCAAGACCAGCTTCAGCAACATCGTGTTCGGCAGCTGGAACGCCATCGACGAGAAG

CTGAGCGACGAGTACGACCTGGCCAACAGCAAGAAGAAGAAGGACGAGAagtactACGAGAAGCGCCAGAAGGAG

CTGAAGAAGAACAAGAGCTACGACCTGGAGACCATCATCGGCCTGTTCGACGACAACAGCGACGTGATCGGCAAG

TACATCGAGAAGCTGGAGAGCGACATCACCGCCATCGCCGAGGCCAAGAACGACTTCGACGAGATCGTGCTGCGC

AAGCACGACAAGAACAAGAGCCTGCGCAAGAACACCAACGCCGTGGAGGCCATCAAGAGCTACCTGGACACCGTG

AAGGACTTCGAGCGCGACATCAAGCTGATCAACGGCAGCGGCCAGGAGGTGGAGAAGAACCTGGTGGTGTACGCC

GAGCAGGAGAACATCCTGGCCGAGATCAAGAACGTGGACAGCCTGTACAACATGAGCCGCAACTACCTGACCCAG

AAGCCCTTCAGCACCGAGAAGTTCAAGCTGAACTTCAACCGCGCCACCCTGCTGAACGGCTGGGACAAGAACAAG

GAGACCGACAACCTGGGCATCCTGTTCGAGAAGGACGGCATGTACTACCTGGGCATCATGAACACCAAGGCCAAC

AAGATCTTCGTGAACATCCCCAAGGCCACCAGCAACGACGTGTACCACAAGGTGAACTACAAGCTGCTGCCCGGC

CCCAACAAGATGCTGCCCAAGGTGTTCTTCGCCCAGAGCAACCTGGACTACTACAAGCCCAGCGAGGAGCTGCTG

GCCAAGTACAAGGCCGGCACCCACAAGAAGGGCGACAACTTCAGCCTGGAGGACTGCCACGCCCTGATCGACTTC

TTCAAGGCCAGCATCGAGAAGCACCCCGACTGGAGCAGCTTCGGCTTCGAGTTCAGCGAGACCTGCACCTACGAG

GACCTGAGCGGCTTCTACCGCGAGGTGGAGAAGCAGGGCTACAAGATCACCTACACCGACGTGGACGCCGACTAC

ATCACCAGCCTGGTGGAGCGCGACGAGCTGTACCTGTTCCAGATCTACAACAAGGACTTCAGCCCCTACAGCAAG

GGCAACCTGAACCTGCACACCATCTACCTGCAGATGCTGTTCGACCAGCGCAACCTGAACAACGTGGTGTACAAG

CTGAACGGCGAGGCCGAGGTGTTCTACCGCCCCGCCAGCATCAACGACGAGGAGGTGATCATCCACAAGGCCGGC

GAGGAGATCAAGAACAAGAACAGCAAGCGCGCCGTGGACAAGCCCACCAGCAAGTTCGGCTACGACATCATCAAG

GACCGCCGCTACAGCAAGGACAAGTTCATGCTGCACATCCCCGTGACCATGAACTTCGGCGTGGACGAGACCCGC

CGCTTCAACGACGTGGTGAACGACGCCCTGCGCAACGACGAGAAGGTGCGCGTGATCGGCATCGACCGCGGCGAG
CGCAACCTGCTGTACGTGGTGGTGGACACCGACGGCACCATCCTGGAGCAGATCAGCCTGAACAGCATCATC
AACAACGAGTACAGCATCGAGACCGACTACCACAAGCTGCTGGACGAGAAGGAGGGCGACCGCGACCGCGCCCGC
AAGAACTGGACCACCATCGAGAACATCAAGGAGCTGAAGGAGGGCTACCTGAGCCAGGTGGTGAACGTGATCGCC
AAGCTGGTGCTGAAGTACAACGCCATCATCTGCCTGGAGGACCTGAACTTCGGCTTCAAGCgcggccgcCAGAAG
GTGGAGAAGCAGGTGTACCAGAAGTTCGAGAAGATGCTGATCGACAAGCTGAACTACCTGGTGATCGACAAGAGC
CGCAAGCAGGACAAGCCCGAGGAGTTCGGCGGCGCCCTGAACGCCCTGCAGCTGACCAGCAAGTTCACCAGCTTC
AAGGACATGGGCAAGCAGACCGGCATCATCTACTACGTGCCCGCCTACCTGACCAGCAAGATCGACCCCACCACC
GGCTTCGCCAACCTGTTCTACGTGAAGTACGAGAACGTGGAGAAGGCCAAGGAGTTCTTCAGCCGCTTCGACAGC
ATCAGCTACAACAACGAGAGCGGCTACTTCGAGTTCGCCTTCGACTACAAGAAGTTCACCGACCGCGCCTGCGGC
GCCCGCAGCCAGTGGACCGTGTGCACCTACGGCGAGCGCATCATCAAGTTCCGCAACACCGAGAAGAACAACAGC
TTCGACGACAAGACCATCGTGCTGAGCGAGGAGTTCAAGGAGCTGTTCAGCATCTACGGCATCAGCTACGAGGAC
GGCGCCGAGCTGAAGAACAAGATCATGAGCGTGGACGAGGCCGACTTCTTCCGCAGCCTGACCCGCCTGTTCCAG
CAGACCATGCAGATGCGCAACAGCAGCAACGACGTGACCCGCGACTACATCATCAGCCCCATCATGAACGACCGC
GGCGAGTTCTTCAACAGCGAGGCCTGCGACGCCAGCAAGCCCAAGGACGCCGACGCCAACGGCGCCTTCAACATC
GCCCGCAAGGGCCTGTGGGTGCTGGAGCAGATCCGCAACACCCCCAGCGGCGACAAGCTGAACCTGGCCATGAGC
AACGCCGAGTGGCTGGAGTACGCCCAGCGCAACCAGATC

>PxCas12a

ATGATCATCGGCCGCGACTTCAACATGTACTACCAGAACCTGACCAAGATGTACCCCATCAGCAAGACCCTGCGC
AACGAGCTGATCCCCGTGGGCAAGACCCTGGAGAACATCCGCAAGAACGGCATCCTGGAGGCCGACATCCAGCGC
AAGGCCGACTACGAGCACGTGAAGAAGCTGATGGACAACTACCACAAGCAGCTGATCAACGAGGCCCTGCAGGGC
GTGCACCTGAGCGACCTGAGCGACGCCTACGACCTGTACTTCAACCTGAGCAAGGAGAAGAACAGCGTGGACGCC
TTCAGCAAGTGCCAGGACAAGCTGCGCAAGGAGATCGTGAGCTTCCTGAAGAACCACGAGAACTTCCCCAAGATC
GGCAACAAGGAGATCATCAAGCTGATCCAGAGCCTGAACGACAACGACGCCGACAACAACGCCCTGGACAGCTTC
AGCAACTTCTACACCTACTTCAGCAGCTACAACGAGGTGCGCAAGAACCTGTACAGCGACGAGGAGAAGAGCAGC
ACCGTGGCCTACCGCCTGATCAACGAGAACCTGCCCAAGAGCCTGGACAACATCAAGGCCTACGCCATCGCCAAG
AAGGCCGGCGTGCGCGCCGAGGGCCTGAGCGAGGAGGAGCAGGACTGCCTGTTCATCATCGAGACCTTCGAGCGC
ACCCTGACCCAGGACGGCATCGACAACTACAACGCCGACATCGGCAAGCTGAACACCGCCATCAACCTGTACAAC
CAGCAGAACAAGAAGCAGGAGGGCTTCCGCAAGGTGCCCCAGATGAAGTGCCTGTACAAGCAGATCCTGAGCGAC
CGCGAGGAGGCCTTCATCGACGAGTTCAGCGACGACGAGGACCTGATCACCAACATCGAGAGCTTCGCCGAGAAC
ATGAACGTGTTCCTGAACAGCGAGATCATCACCGACTTCAAGAACGCCCTGGTGGAGAGCGACGGCAGCCTGGTG
TACATCAAGAACGACGTGAGCAAGACCCTGTTCAGCAACATCGTGTTCGGCAGCTGGAACGCCATCGACGAGAAG
CTGAGCGACGAGTACGACCTGGCCAACAGCAAGAAGAAGAAGGACGAGAagtactACGAGAAGCGCCAGAAGGAG
CTGAAGAAGAACAAGAGCTACGACCTGGAGACCATCATCGGCCTGTTCGACGACAGCATCGACGTGATCGGCAAG
TACATCGAGAAGCTGGAGAGCGACATCACCGCCATCGCCGAGGCCAAGAACGACTTCGACGAGATCGTGCTGCGC
AAGCACGACAAGAACAAGAGCCTGCGCAAGAACACCAACGCCGTGGAGGCCATCAAGAGCTACCTGGACACCGTG
AAGGACTTCGAGCGCGACATCAAGCTGATCAACGGCAGCGGCCAGGAGGTGGAGAAGAACCTGGTGGTGTACGCC
GAGCAGGAGAACATCCTGGCCGAGATCAAGAACGTGGACAGCCTGTACAACATGAGCCGCAACTACCTGACCCAG
AAGCCCTTCAGCACCGAGAAGTTCAAGCTGAACTTCGAGAACCCCACCCTGCTGAACGGCTGGGACCGCAACAAG
GAGAAGGACTACCTGGGCATCCTGTTCGAGAAGGAGGGCATGTACTACCTGGGCATCATCAACAACAACCACCGC
AAGATCTTCGAGAACGAGAAGCTGTGCACCGGCAAGGAGAGCTGCTTCAACAAGATCGTGTACAAGCAGATCAGC
AACGCCGCCAAGTACCTGAGCAGCAAGCAGATCAACCCCCAGAACCCCCCCAAGGAGATCGCCGAGATCCTGCTG

AAGCGCAAGGCCGACAGCAGCAGCCTGAGCCGCAAGGAGACCGAGCTGTTCATCGACTACCTGAAGGACGACTTC
CTGGTGAACTACCCCATGATCATCAACAGCGACGGCGAGAACTTCTTCAACTTCCACTTCAAGCAGGCCAAGGAC
TACGGCAGCCTGCAGGAGTTCTTCAAGGAGGTGGAGCACCAGGCCTACAGCCTGAAGACCCGCCCCATCGACGAC
AGCTACATCTACCGCATGATCGACGAGGGCAAGCTGTACCTGTTCCAGATCCACAACAAGGACTTCAGCCCCTAC
AGCAAGGGCAACCTGAACCTGCACACCATCTACCTGCAGATGCTGTTCGACCAGCGCAACCTGAACAACGTGGTG
TACAAGCTGAACGGCGAGGCCGAGGTGTTCTACCGCCCCGCCAGCATCAACGACGAGGAGGTGATCATCCACAAG
GCCGGCGAGGAGATCAAGAACAAGAACAGCAAGCGCGCCGTGGACAAGCCCACCAGCAAGTTCGGCTACGACATC
ATCAAGGACCGCCGCTACAGCAAGGACAAGTTCATGCTGCACATCCCCGTGACCATGAACTTCGGCGTGGACGAG
ACCCGCCGCTTCAACGACGTGGTGAACGACGCCCTGCGCAACGACGAGAAGGTGCGCGTGATCGGCATCGACCGC
GGCGAGCGCAACCTGCTGTACGTGGTGGTGGACACCGACGGCACCATCCTGGAGCAGATCAGCCTGAACAGC
ATCATCAACAACGAGTACAGCATCGAGACCGACTACCACAAGCTGCTGGACGAGAAGGAGGGCGACCGCGACCGC
GCCCGCAAGAACTGGACCACCATCGAGAACATCAAGGAGCTGAAGGAGGGCTACCTGAGCCAGGTGGTGAACGTG
ATCGCCAAGCTGGTGCTGAAGTACAACGCCATCATCTGCCTGGAGGACCTGAACTTCGGCTTCAAGCgcggccgc
CAGAAGGTGGAGAAGCAGGTGTACCAGAAGTTCGAGAAGATGCTGATCGACAAGCTGAACTACCTGGTGATCGAC
AAGAGCCGCAAGCAGGAGAAGCCCGAGGAGTTCGGCGGCGCCCTGAACGCCCTGCAGCTGACCAGCAAGTTCACC
AGCTTCAAGGACATGGGCAAGCAGACCGGCATCATCTACTACGTGCCCGCCTACCTGACCAGCAAGATCGACCCC
ACCACCGGCTTCGCCAACCTGTTCTACGTGAAGTACGAGAACGTGGAGAAGGCCAAGGAGTTCTTCAGCCGCTTC
GACAGCATCAGCTACAACAACGAGAGCGGCTACTTCGAGTTCGCCTTCGACTACAAGAAGTTCACCGACCGCGCC
TGCGGCGCCCGCAGCCAGTGGACCGTGTGCACCTACGGCGAGCGCATCATCAAGTACCGCAACGCCGACAAGAAC
AACAGCTTCGACGACAAGACCATCGTGCTGAGCGAGGAGTTCAAGGAGCTGTTCAGCATCTACGGCATCAGCTAC
GAGGACGGCGCCGAGCTGAAGAACAAGATCATGAGCGTGGACGAGGCCGACTTCTTCCGCTGCCTGACCGGCCTG
CTGCAGAAGACCCTGCAGATGCGCAACAGCAGCAACGACGGCACCCGCGACTACATCATCAGCCCCATCATGAAC
GACCGCGGCGAGTTCTTCAACAGCGAGGCCTGCGACGCCAGCAAGCCCAAGGACGCCGACGCCAACGGCGCCTTC
AACATCGCCCGCAAGGGCCTGTGGGTGCTGGAGCAGATCCGCAACACCCCCAGCGGCGACAAGCTGAACCTGGCC
ATGAGCAACGCCGAGTGGCTGGAGTACGCCCAGCGCAACCAGATC

>Mb3Cas12a

GGTATCCACGGAGTCCCAGCAGCCCTGTTCCAGGACTTCACCCACCTGTACCCCCTGAGCAAGACCGTCAGATTC
GAGCTGAAGCCCATCGGCAAGACCCTGGAACACATCCACGCCAAGAACTTTCTGAACCAGGACGAGACAATGGCC
GACATGTACCAGAAAGTGAAGGCCATCCTGGACGACTACCACCGGGACTTTATCGCCGACATGATGGGCGAAGTG
AAGCTGACCAAGCTGGCCGAGTTCTACGACGTGTACCTGAAGTTCCGGAAGAACCCCAAGGACGACGGCCTGCAG
AAGCAGCTGAAAGACCTGCAGGCTGTGCTGCGGAAAGAAATCGTGAAGCCTATCGGAAACGGCGGCAAGTACAAG
GCCGGCTACGACAGACTGTTCGGCGCCAAGCTGTTCAAGGACGGCAAAGAGCTGGGCGACCTGGCCAAGTTCGTG
ATCGCCCAGGAAGGCGAGAGCAGCCCTAAGCTGGCCCACCTGGCCCATTTCGAGAAGTTCAGCACCTACTTCACC
GGCTTCCACGACAACCGCAAGAACATGTACAGCGACGAGGACAAGCACACCGCCATTGCCTACCGGCTGATCCAC
GAGAACCTGCCCCGGTTCATCGACAACCTGCAGATCCTGGCCACCATCAAGCAGAAGCACAGCGCCCTGTACGAC
CAGATCATCAACGAGCTGACCGCCAGCGGCCTGGATGTGTCTCTGGCCTCTCACCTGGATGGCTACCACAAGCTG
CTGACACAGGAAGGCATCACCGCCTACAACACCCTGCTGGGCGGAATCTCTGGCGAGGCCGGCAGCAGAAAGATC
CAGGGCATCAATGAACTGATCAACAGCCACCACAACCAGCACTGCCACAAGAGCGAGCGGATTGCCAAGCTGCGG
CCCCTGCACAAGCAGATTCTGAGCGACGGAATGGGCGTGTCCTTCCTGCCCAGCAAGTTCGCCGACGACAGCGAA
GTGTGCCAGGCCGTGAATGAGTTCTACCGGCACTACGCCGACGTGTTCGCCAAGGTGCAGAGCCTGTTCGACGGC
TTCGACGACTATCAGAAAGACGGCATCTACGTGGAGTACAAGAACCTGAACGAGCTGTCCAAGCAGGCCTTCGGC
GACTTCGCTCTGCTGGGAAGAGTGCTGGACGGCTACTATGTGGACGTCGTGAACCCCGAGTTCAACGAGAGATTT

GCCAAGGCCAAGACCGACAACGCCAAAGCCAAGCTGACAAAAGAGAAGGACAAGTTCATCAAGGGCGTGCACTCC
CTGGCTTCTCTGGAACAGGCCATCGAGCACTACACCGCCAGACACGACGACGAGTCTGTGCAGGCCGGAAAGCTG
GGCCAGTACTTCAAGCACGGACTGGCCGGCGTGGACAACCCCATCCAGAAGATCCACAACAACCACTCTACAATC
AAGGGCTTCCTGGAAAGAGAGCGGCCTGCCGGCGAAAGAGCCCTGCCCAAGATCAAGAGCGACAAGAGCCCCGAG
ATCAGACAGCTGAAAGAGCTGCTGGACAACGCCCTGAACGTGGCCCACTTCGCCAAACTGCTGACCACCAAGACC
ACACTGCACAATCAGGATGGCAACTTCTACGGCGAGTTCGGAGCCCTGTATGATGAGCTGGCCAAGATCGCCACC
CTGTACAACAAAGTGCGGGACTACCTGAGCCAGAAGCCCTTCAGCACCGAGAAGTACAAGCTGAATTTCGGCAAC
CCTACCCTGCTGAACGGCTGGGACCTGAACAAAGAGAAAGATAACTTCGGCGTGATCCTGCAGAAGGACGGCTGC
TACTACCTGGCCCTGCTGGATAAGGCCCACAAGAAGGTGTTCGATAACGCCCCCAACACCGGCAAGTCTGTGTAT
CAGAAGATGATCTATAAGCTGCTGCCTGGCCCCAACAAGATGCTGCCCAAGGTGTTCTTCGCCAAGAGCAACCTG
GACTACTACAACCCCAGCGCCGAACTGCTGGACAAATACGCCCAGGGCACACACAAGAAAGGCGACAACTTCAAC
CTGAAGGACTGCCACGCCCTGATCGATTTCTTCAAGGCCGGAATCAACAAGCACCCCGAGTGGCAGCACTTCGGC
TTCAAGTTCAGCCCCACCAGCAGCTACCAGGACCTGAGCGACTTCTACAGAGAGGTGGAACCCCAGGGCTACCAA
GTGAAGTTCGTGGACATCAACGCCGACTACATCAATGAGCTGGTGGAACAGGGCCAGCTGTACCTGTTTCAGATC
TACAACAAGGACTTTAGCCCCAAGGCCCACGGCAAGCCCAACCTGCACACCCTGTATTTCAAGGCCCTGTTTAGC
GAGGACAACCTCGTGAATCCCATCTACAAACTGAACGGGGAGGCCGAGATCTTCTACCGGAAGGCCTCCCTGGAC
ATGAACGAGACAACCATCCACAGAGCCGGCGAGGTGCTGGAAAACAAGAACCCTGACAACCCTAAGAAACGGCAG
TTCGTGTACGACATCATCAAGGACAAGCGGTACACCCAGGATAAGTTCATGCTGCACGTGCCCATCACCATGAAC
TTCGGAGTGCAGGGCATGACCATCAAAGAGTTCAACAAAAAAGTGAACCAGAGCATCCAGCAGTACGACGAAGTG
AACGTGATCGGCATCGACCGGGGCGAGCGGCATCTGCTGTATCTGACCGTGATCAACTCCAAGGGCGAGATTCTG
GAACAGAGATCCCTGAACGACATCACCACCGCCTCCGCCAACGGCACCCAGATGACCACCCCTTACCACAAGATC
CTGGACAAGCGCGAGATCGAGCGGCTGAACGCCAGAGTGGGATGGGGAGAGATCGAGACAATCAAAGAACTGAAG
TCCGGCTACCTGTCCCACGTGGTGCATCAGATCTCCCAGCTGATGCTGAAGTACAACGCCATCGTGGTGCTGGAA
GATCTGAATTTTGGCTTCAAGAGGGGCCGGTTCAAGGTGGAAAAGCAGATCTACCAGAACTTCGAGAATGCCCTG
ATCAAGAAACTGAACCACCTGGTGCTGAAAGACAAGGCCGACGACGAGATCGGCAGCTACAAGAACGCCCTGCAG
CTGACTAACAACTTCACCGATCTGAAGAGTATCGGCAAGCAGACCGGCTTTCTGTTCTACGTGCCCGCCTGGAAT
ACCAGCAAGATCGACCCCGAGACAGGCTTCGTGGACCTGCTGAAGCCTAGATACGAGAATATCGCCCAGAGCCAG
GCCTTCTTCGGCAAGTTCGACAAGATCTGCTACAACGCCGATAGGGGCTACTTCGAGTTCCACATCGACTACGCC
AAGTTCAATGACAAGGCCAAAAACAGCCGGCAGATCTGGAAAATCTGCAGCCACGGCGATAAGCGCTACGTGTAC
GATAAGACCGCCAACCAGAACAAGGGCGCCACCATCGGAGTGAATGTGAACGATGAGCTGAAGTCCCTGTTCACC
CGCTACCACATCAACGATAAGCAGCCCAATCTCGTGATGGACATCTGCCAGAACAACGACAAAGAGTTTCACAAG
AGCCTGATGTATCTGCTGAAAACACTGCTGGCTCTGCGGTACAGCAACGCCAGCTCCGACGAGGATTTCATCCTG
AGCCCCGTGGCCAACGACGAGGGCGTGTTCTTCAATAGCGCCCTGGCCGACGATACCCAGCCCCAGAATGCCGAT
GCCAACGGCGCCTACCACATTGCCCTGAAGGGACTGTGGCTGCTGAACGAACTGAAGAACAGCGACGATCTGAAC
AAAGTGAAGCTGGCCATCGACAACCAGACCTGGCTGAATTTCGCCCAGAACAGA

>PdCas12a

ATGGAGAACTATCAGGAGTTCACCAACCTGTTTCAGCTGAATAAGACACTGAGATTCGAGCTGAAGCCCATCGGC
AAGACCTGCGAGCTGCTGGAGGAGGGCAAGATCTTCGCCAGCGGCTCCTTTCTGGAGAAGGACAAGGTGAGGGCC
GATAACGTGAGCTACGTGAAGAAGGAGATCGACAAGAAGCACAAGATCTTTATCGAGGAGACACTGAGCTCCTTC
TCTATCAGCAACGATCTGCTGAAGCAGTACTTTGACTGCTATAATGAGCTGAAGGCCTTCAAGAAGGACTGTAAG
AGCGATGAGGAGGAGGTGAAGAAAACCGCCCTGCGCAACAAGTGTACCTCCATCCAGAGGGCCATGCGCGAGGCC
ATCTCTCAGGCCTTTCTGAAGAGCCCCCAGAAGAAGCTGCTGGCCATCAAGAACCTGATCGAGAACGTGTTCAAG

GCCGACGAGAATGTGCAGCACTTCTCCGAGTTTACCAGCTATTTCTCCGGCTTTGAGACAAACAGAGAGAATTTC
TACTCTGACGAGGAGAAGTCCACATCTATCGCCTATAGGCTGGTGCACGATAACCTGCCTATCTTCATCAAGAAC
ATCTACATCTTCGAGAAGCTGAAGGAGCAGTTCGACGCCAAGACCCTGAGCGAGATCTTCGAGAACTACAAGCTG
TATGTGGCCGGCTCTAGCCTGGATGAGGTGTTCTCCCTGGAGTACTTTAACAATACCCTGACACAGAAGGGCATC
GACAACTATAATGCCGTGATCGGCAAGATCGTGAAGGAGGATAAGCAGGAGATCCAGGGCCTGAACGAGCACATC
AACCTGTATAATCAGAAGCACAAGGACCGGAGACTGCCCTTCTTTATCTCCCTGAAGAAGCAGATCCTGTCCGAT
CGGGAGGCCCTGTCTTGGCTGCCTGACATGTTCAAGAATGATTCTGAAGTGATCAAGGCCCTGAAGGGCTTCTAC
ATCGAGGACGGCTTTGAGAACAATGTGCTGACACCTCTGGCCACCCTGCTGTCCTCTCTGGATAAGTACAACCTG
AATGGCATCTTTATCCGCAACAATGAGGCCCTGAGCTCCCTGTCCCAGAACGTGTATCGGAATTTTTCTATCGAC
GAGGCCATCGATGCCAACGCCGAGCTGCAGACCTTCAACAATTACGAGCTGATCGCCAATGCCCTGCGCGCCAAG
ATCAAGAAGGAGACAAAGCAGGGCCGGAAGTCTTTCGAGAAGTACGAGGAGTATATCGATAAGAAGGTGAAGGCC
ATCGACAGCCTGTCCATCCAGGAGATCAACGAGCTGGTGGAGAATTACGTGAGCGAGTTTAACTCTAATAGCGGC
AACATGCCAAGAAAGGTGGAGGACTACTTCAGCCTGATGAGGAAGGGCGACTTCGGCTCCAACGATCTGATCGAA
AATATCAAGACCAAGCTGAGCGCCGCAGAGAAGCTGCTGGGCACAAAGTACCAGGAGACAGCCAAGGACATCTTC
AAGAAGGATGAGAACTCCAAGCTGATCAAGGAGCTGCTGGACGCCACCAAGCAGTTCCAGCACTTTATCAAGCCA
CTGCTGGGCACAGGCGAGGAGGCAGATCGGGACCTGGTGTTCTACGGCGATTTTCTGCCCCTGTATGAGAAGTTT
GAGGAGCTGACCCTGCTGTATAACAAGGTGCGGAATAGACTGACACAGAAGCCCTATTCCAAGGACAAGATCCGC
CTGTGCTTCAACAAGCCTAAGCTGATGACAGGCTGGGTGGATTCCAAGACCGAGAAGTCTGACAACGGCACACAG
TACGGCGGCTATCTGTTTCGGAAGAAGAATGAGATCGGCGAGTACGATTATTTTCTGGGCATCTCTAGCAAGGCC
CAGCTGTTCAGAAAGAACGAGGCCGTGATCGGCGACTACGAGAGGCTGGATTACTATCAGCCAAAGGCCAATACC
ATCTACGGCTCTGCCTATGAGGGCGAGAACAGCTACAAGGAGGACAAGAAGCGGCTGAACAAAGTGATCATCGCC
TATATCGAGCAGATCAAGCAGACAAACATCAAGAAGTCTATCATCGAGTCCATCTCTAAGTATCCTAATATCAGC
GACGATGACAAGGTGACCCCATCCTCTCTGCTGGAGAAGATCAAGAAGGTGTCTATCGACAGCTACAACGGCATC
CTGTCCTTCAAGTCTTTTCAGAGCGTGAACAAGGAAGTGATCGATAACCTGCTGAAAACCATCAGCCCCCTGAAG
AACAAGGCCGAGTTTCTGGACCTGATCAATAAGGATTATCAGATCTTCACCGAGGTGCAGGCCGTGATCGACGAG
ATCTGCAAGCAGAAAACCTTCATCTACTTTCCAATCTCCAACGTGGAGCTGGAGAAGGAGATGGGCGATAAGGAC
AAGCCCCTGTGCCTGTTCCAGATCAGCAATAAGGATCTGTCCTTCGCCAAGACCTTTAGCGCCAACCTGCGGAAG
AAGAGAGGCGCCGAGAATCTGCACACAATGCTGTTTAAGGCCCTGATGGAGGGCAACCAGGATAATCTGGACCTG
GGCTCTGGCGCCATCTTCTACAGAGCCAAGAGCCTGGACGGCAACAAGCCCACACACCCTGCCAATGAGGCCATC
AAGTGTAGGAACGTGGCCAATAAGGATAAGGTGTCCCTGTTCACCTACGACATCTATAAGAACAGGCGCTACATG
GAGAATAAGTTCCTGTTTCACCTGAGCATCGTGCAGAACTATAAGGCCGCCAATGACTCCGCCCAGCTGAACAGC
TCCGCCACCGAGTATATCAGAAAGGCCGATGACCTGCACATCATCGGCATCGATAGGGGCGAGCGCAATCTGCTG
TACTATTCCGTGATCGATATGAAGGGCAACATCGTGGAGCAGGACTCTCTGAATATCATCAGGAACAATGACCTG
GAGACAGATTACCACGACCTGCTGGATAAGAGGGAGAAGGAGCGCAAGGCCAACCGGCAGAATTGGGAGGCCGTG
GAGGGCATCAAGGACCTGAAGAAGGGCTACCTGAGCCAGGCCGTGCACCAGATCGCCCAGCTGATGCTGAAGTAT
AACGCCATCATCGCCCTGGAGGATCTGGGCCAGATGTTTGTGACCCGCGGCCAGAAGATCGAGAAGGCCGTGTAC
CAGCAGTTCGAGAAGAGCCTGGTGGATAAGCTGTCCTACCTGGTGGACAAGAAGCGGCCTTATAATGAGCTGGGC
GGCATCCTGAAGGCCTACCAGCTGGCCTCTAGCATCACCAAGAACAATTCTGACAAGCAGAACGGCTTCCTGTTT
TATGTGCCAGCCTGGAATACAAGCAAGATCGATCCCGTGACCGGCTTTACAGACCTGCTGCGGCCCAAGGCCATG
ACCATCAAGGAGGCCCAGGACTTCTTTGGCGCCTTCGATAACATCTCTTACAATGACAAGGGCTATTTCGAGTTT
GAGACAAACTACGACAAGTTTAAGATCAGAATGAAGAGCGCCCAGACCAGGTGGACAATCTGCACCTTCGGCAAT
CGGATCAAGAGAAAGAAGGATAAGAACTACTGGAATTATGAGGAGGTGGAGCTGACCGAGGAGTTCAAGAAGCTG
TTTAAGGACAGCAACATCGATTACGAGAACTGTAATCTGAAGGAGGAGATCCAGAACAAGGACAATCGCAAGTTC

TTTGATGACCTGATCAAGCTGCTGCAGCTGACACTGCAGATGCGGAACTCCGATGACAAGGGCAATGATTATATC
ATCTCTCCTGTGGCCAACGCCGAGGGCCAGTTCTTTGACTCCCGCAATGGCGATAAGAAGCTGCCACTGGATGCA
GACGCAAACGGAGCCTACAATATCGCCCGCAAGGGCCTGTGGAACATCCGGCAGATCAAGCAGACCAAGAACGAC
AAGAAGCTGAATCTGAGCATCTCCTCTACAGAGTGGCTGGATTTCGTGCGGGAGAAGCCTTACCTGAAG

>PiCas12a

ATGGAGAACTATCAGGAGTTCACCAACCTGTTTCAGCTGAATAAGACACTGAGATTCGAGCTGAAGCCCATCGGC
AAGACCTGCGAGCTGCTGGAGGAGGGCAAGATCTTCGCCAGCGGCTCCTTTCTGGAGAAGGACAAGGTGAGGGCC
GATAACGTGAGCTACGTGAAGAAGGAGATCGACAAGAAGCACAAGATCTTTATCGAGGAGACACTGAGCTCCTTC
TCTATCAGCAACGATCTGCTGAAGCAGTACTTTGACTGCTATAATGAGCTGAAGGCCTTCAAGAAGGACTGTAAG
AGCGATGAGGAGGAGGTGAAGAAAACCGCCCTGCGCAACAAGTGTACCTCCATCCAGAGGGCCATGCGCGAGGCC
ATCTCTCAGGCCTTTCTGAAGAGCCCCCAGAAGAAGCTGCTGGCCATCAAGAACCTGATCGAGAACGTGTTCAAG
GCCGACGAGAATGTGCAGCACTTCTCCAAGTTTACCAGCTATTTCTCCGGCTTTGAGACAAACAGAGAGAATTTC
TACTCTGACGAGGAGAAGTCCACATCTATCGCCTATAGGCTGGTGCACGATAACCTGCCTATCTTCATCAAGAAC
ATCTACATCTTCGAGAAGCTGAAGGAGCAGTTCGACGCCAAGACCCTGAGCGAGATCTTCGAGAACTACAAGCTG
TATGTGGCCGGCTCTAGCCTGGATGAGGTGTTCTCCCTGGAGTACTTTAACAATACCCTGACACAGAAGGGCATC
GACAACTATAATGCCGTGATCGGCAAGATCGTGAAGGAGGATAAGCAGGAGATCCAGGGCCTGAACGAGCACATC
AACCTGTATAATCAGAAGCACAAGGACCGGAGACTGCCCTTCTTTATCTCCCTGAAGAAGCAGATCCTGTCCGAT
CGGGAGGCCCTGTCTTGGCTGCCTGACATGTTCAAGAATGATTCTGAAGTGATCAAGGCCCTGAAGGGCTTCTAC
ATCGAGGACGGCTTTGAGAACAATGTGCTGACACCTCTGGCCACCCTGCTGTCCTCTCTGGATAAGTACAACCTG
AATGGCATCTTTATCCGCAACAATGAGGCCCTGAGCTCCCTGTCCCAGAACGTGTATCGGAATTTTTCTATCGAC
GAGGCCATCGATGCCAACGCCGAGCTGCAGACCTTCAACAATTACGAGCTGATCGCCAATGCCCTGCGCGCCAAG
ATCAAGAAGGAGACAAAGCAGGGCCGGAAGTCTTTCGAGAAGTACGAGGAGTATATCGATAAGAAGGTGAAGGCC
ATCGACAGCCTGTCCATCCAGGAGATCAACGAGCTGGTGGAGAATTACGTGAGCGAGTTTAACTCTAATAGCGGC
AACATGCCAAGAAAGGTGGAGGACTACTTCAGCCTGATGAGGAAGGGCGACTTCGGCTCCAACGATCTGATCGAA
AATATCAAGACCAAGCTGAGCGCCGCAGAGAAGCTGCTGGGCACAAAGTACCAGGAGACAGCCAAGGACATCTTC
AAGAAGGATGAGAACTCCAAGCTGATCAAGGAGCTGCTGGACGCCACCAAGCAGTTCCAGCACTTTATCAAGCCA
CTGCTGGGCACAGGCGAGGAGGCAGATCGGGACCTGGTGTTCTACGGCGATTTTCTGCCCCTGTATGAGAAGTTT
GAGGAGCTGACCCTGCTGTATAACAAGGTGCGGAATAGACTGACACAGAAGCCCTATTCCAAGGACAAGATCCGC
CTGTGCTTCAACAAGCCTAAGCTGATGACAGGCTGGGTGGATTCCAAGACCGAGAAGTCTAACAGCGGCACACAG
TTCGGCGGCTATCTGTTTCGGAAGAAGAATGAGATCGGCGAGTACGATTATTTTCTGGGCATCTCTAGCAAGACC
CAGCTGTTCAGAAAGAACGAGGCCGTGATCGGCGACTACGAGAGGCTGGATTACTATCAGCCAAAGGCCAATACC
ATCTACGGCTCTGCCTATGAGGGCGAGAACAGCTACAAGGAGGACAAGAAGCGGCTGAACAAAGTGATCATCGCC
TATATCGAGCAGATCAAGCAGACAAACATCAAGAAGTCTATCATCGAGTCCATCTCTAAGTATCCTAATATCAGC
GACGATGACAAGGTGACCCCATCCTCTCTGCTGGAGAAGATCAAGAAGGTGTCTATCGACAGCTACAACGGCATC
CTGTCCTTCAAGTCTTTTCAGAGCGTGAACAAGGAAGTGATCGATAACCTGCTGAAAACCATCAGCCCCCTGAAG
AACAAGGCCGAGTTTCTGGACCTGATCAATAAGGATTATCAGATCTTCACCGAGGTGCAGGCCGTGATCGACGAG
ATCTGCAAGCAGAAAACCTTCATCTACTTTCCAATCTCCAACGTGGAGCTGGAGAAGGAGATGGGCGATAAGGAC
AAGCCCCTGTGCCTGTTCCAGATCAGCAATAAGGATCTGTCCTTCGCCAAGACCTTTAGCGCCAACCTGCGGAAG
AAGAGAGGCGCCGAGAATCTGCACACAATGCTGTTTAAGGCCCTGATGGAGGGCAACCAGGATAATCTGGACCTG
GGCTCTGGCGCCATCTTCTACAGAGCCAAGAGCCTGGACGGCAACAAGCCCACACACCCTGCCAATGAGGCCATC
AAGTGTAGGAACGTGGCCAATAAGGATAAGGTGTCCCTGTTCACCTACGACATCTATAAGAACAGGCGCTACATG
GAGAATAAGTTCCTGTTTCACCTGAGCATCGTGCAGAACTATAAGGCCGCCAATGACTCCGCCCAGCTGAACAGC

TCCGCCACCGAGTATATCAGAAAGGCCGATGACCTGCACATCATCGGCATCGATAGGGGCGAGCGCAATCTGCTG
TACTATTCCGTGATCGATATGAAGGGCAACATCGTGGAGCAGGACTCTCTGAATATCATCAGGAACAATGACCTG
GAGACAGATTACCACGACCTGCTGGATAAGAGGGAGAAGGAGCGCAAGGCCAACCGGCAGAATTGGGAGGCCGTG
GAGGGCATCAAGGACCTGAAGAAGGGCTACCTGAGCCAGGCCGTGCACCAGATCGCCCAGCTGATGCTGAAGTAT
AACGCCATCATCGCCCTGGAGGATCTGGGCCAGATGTTTGTGACCCGCGGCCAGAAGATCGAGAAGGCCGTGTAC
CAGCAGTTCGAGAAGAGCCTGGTGGATAAGCTGTCCTACCTGGTGGACAAGAAGCGGCCTTATAATGAGCTGGGC
GGCATCCTGAAGGCCTACCAGCTGGCCTCTAGCATCACCAAGAACAATTCTGACAAGCAGAACGGCTTCCTGTTT
TATGTGCCAGCCTGGAATACAAGCAAGATCGATCCCGTGACCGGCTTTACAGACCTGCTGCGGCCCAAGGCCATG
ACCATCAAGGAGGCCCAGGACTTCTTTGGCGCCTTCGATAACATCTCTTACAATGACAAGGGCTATTTCGAGTTT
GAGACAAACTACGACAAGTTTAAGATCAGAATGAAGAGCGCCCAGACCAGGTGGACAATCTGCACCTTCGGCAAT
CGGATCAAGAGAAAGAAGGATAAGAACTACTGGAATTATGAGGAGGTGGAGCTGACCGAGGAGTTCAAGAAGCTG
TTTAAGGACAGCAACATCGATTACGAGAACTGTAATCTGAAGGAGGAGATCCAGAACAAGGACAATCGCAAGTTC
TTTGATGACCTGATCAAGCTGCTGCAGCTGACACTGCAGATGCGGAACTCCGATGACAAGGGCAATGATTATATC
ATCTCTCCTGTGGCCAACGCCGAGGGCCAGTTCTTTGACTCCCGCAATGGCGATAAGAAGCTGCCACTGGATGCA
GACGCAAACGGAGCCTACAATATCGCCCGCAAGGGCCTGTGGAACATCCGGCAGATCAAGCAGACCAAGAACGAC
AAGAAGCTGAATCTGAGCATCTCCTCTACAGAGTGGCTGGATTTCGTGCGGGAGAAGCCTTACCTGAAG

>Lb2Cas12a

ATGTACTATGAGTCCCTGACCAAGCAGTACCCCGTGTCTAAGACAATCCGGAATGAGCTGATCCCTATCGGCAAG
ACACTGGATAACATCCGCCAGAACAATATCCTGGAGAGCGACGTGAAGCGGAAGCAGAACTACGAGCACGTGAAG
GGCATCCTGGATGAGTATCACAAGCAGCTGATCAACGAGGCCCTGGACAATTGCACCCTGCCATCCCTGAAGATC
GCCGCCGAGATCTACCTGAAGAATCAGAAGGAGGTGTCTGACAGAGAGGATTTCAACAAGACACAGGACCTGCTG
AGGAAGGAGGTGGTGGAGAAGCTGAAGGCCCACGAGAACTTTACCAAGATCGGCAAGAAGGACATCCTGGATCTG
CTGGAGAAGCTGCCTTCCATCTCTGAGGACGATTACAATGCCCTGGAGAGCTTCCGCAACTTTTACACCTATTTC
ACATCCTACAACAAGGTGCGGGAGAATCTGTATTCTGATAAGGAGAAGAGCTCCACAGTGGCCTACAGACTGATC
AACGAGAATTTCCCAAAGTTTCTGGACAATGTGAAGAGCTATAGGTTTGTGAAAACCGCAGGCATCCTGGCAGAT
GGCCTGGGAGAGGAGGAGCAGGACTCCCTGTTCATCGTGGAGACATTCAACAAGACCCTGACACAGGACGGCATC
GATACCTACAATTCTCAAGTGGGCAAGATCAACTCTAGCATCAATCTGTATAACCAGAAGAATCAGAAGGCCAAT
GGCTTCAGAAAGATCCCCAAGATGAAGATGCTGTATAAGCAGATCCTGTCCGATAGGGAGGAGTCTTTCATCGAC
GAGTTTCAGAGCGATGAGGTGCTGATCGACAACGTGGAGTCTTATGGCAGCGTGCTGATCGAGTCTCTGAAGTCC
TCTAAGGTGAGCGCCTTCTTTGATGCCCTGAGAGAGTCTAAGGGCAAGAACGTGTACGTGAAGAATGACCTGGCC
AAGACAGCCATGAGCAACATCGTGTTCGAGAATTGGAGGACCTTTGACGATCTGCTGAACCAGGAGTACGACCTG
GCCAACGAGAACAAGAAGAAGGACGATAAGTATTTCGAGAAGCGCCAGAAGGAGCTGAAGAAGAATAAGAGCTAC
TCCCTGGAGCACCTGTGCAACCTGTCCGAGGATTCTTGTAACCTGATCGAGAATTATATCCACCAGATCTCCGAC
GATATCGAGAATATCATCATCAACAATGAGACATTCCTGCGCATCGTGATCAATGAGCACGACAGGTCCCGCAAG
CTGGCCAAGAACCGGAAGGCCGTGAAGGCCATCAAGGACTTTCTGGATTCTATCAAGGTGCTGGAGCGGGAGCTG
AAGCTGATCAACAGCTCCGGCCAGGAGCTGGAGAAGGATCTGATCGTGTACTCTGCCCACGAGGAGCTGCTGGTG
GAGCTGAAGCAGGTGGACAGCCTGTATAACATGACCAGAAATTATCTGACAAAGAAGCCTTTCTCTACCGAGAAG
GTGAAGCTGAACTTTAATCGCAGCACACTGCTGAACGGCTGGGATCGGAATAAGGAGACAGACAACCTGGGCGTG
CTGCTGCTGAAGGACGGCAAGTACTATCTGGGCATCATGAACACAAGCGCCAATAAGGCCTTCGTGAATCCCCCT
GTGGCCAAGACCGAGAAGGTGTTTAAGAAGGTGGATTACAAGCTGCTGCCAGTGCCCAACCAGATGCTGCCAAAG
GTGTTCTTTGCCAAGAGCAATATCGACTTCTATAACCCCTCTAGCGAGATCTACTCCAATTATAAGAAGGGCACC
CACAAGAAGGGCAATATGTTTTCCCTGGAGGATTGTCACAACCTGATCGACTTCTTTAAGGAGTCTATCAGCAAG

CACGAGGACTGGAGCAAGTTCGGCTTTAAGTTCAGCGATACAGCCTCCTACAACGACATCTCCGAGTTCTATCGC
GAGGTGGAGAAGCAGGGCTACAAGCTGACCTATACAGACATCGATGAGACATACATCAATGATCTGATCGAGCGG
AACGAGCTGTACCTGTTCCAGATCTATAATAAGGACTTTAGCATGTACTCCAAGGGCAAGCTGAACCTGCACACA
CTGTATTTCATGATGCTGTTTGATCAGCGCAATATCGACGACGTGGTGTATAAGCTGAACGGAGAGGCAGAGGTG
TTCTATAGGCCAGCCTCCATCTCTGAGGACGAGCTGATCATCCACAAGGCCGGCGAGGAGATCAAGAACAAGAAT
CCTAACCGGGCCAGAACCAAGGAGACAAGCACCTTCAGCTACGACATCGTGAAGGATAAGCGGTATAGCAAGGAT
AAGTTTACCCTGCACATCCCCATCACAATGAACTTCGGCGTGGATGAGGTGAAGCGGTTCAACGACGCCGTGAAC
AGCGCCATCCGGATCGATGAGAATGTGAACGTGATCGGCATCGACCGGGGCGAGAGAAATCTGCTGTACGTGGTG
GTCATCGACTCTAAGGGCAACATCCTGGAGCAGATCTCCCTGAACTCTATCATCAATAAGGAGTACGACATCGAG
ACAGATTATCACGCACTGCTGGATGAGAGGGAGGGCGGCAGAGATAAGGCCCGGAAGGACTGGAACACCGTGGAG
AATATCAGGGACCTGAAGGCCGGCTACCTGAGCCAGGTGGTGAACGTGGTGGCCAAGCTGGTGCTGAAGTATAAT
GCCATCATCTGCCTGGAGGACCTGAACTTTGGCTTCAAGAGGGGCCGCCAGAAGGTGGAGAAGCAGGTGTACCAG
AAGTTCGAGAAGATGCTGATCGATAAGCTGAATTACCTGGTCATCGACAAGAGCCGCGAGCAGACATCCCCTAAG
GAGCTGGGAGGCGCCCTGAACGCACTGCAGCTGACCTCTAAGTTCAAGAGCTTTAAGGAGCTGGGCAAGCAGTCC
GGCGTGATCTACTATGTGCCTGCCTACCTGACCTCTAAGATCGATCCAACCACAGGCTTCGCCAATCTGTTTTAT
ATGAAGTGTGAGAACGTGGAGAAGTCCAAGAGATTCTTTGACGGCTTTGATTTCATCAGGTTCAACGCCCTGGAG
AACGTGTTCGAGTTCGGCTTTGACTACCGGAGCTTCACCCAGAGGGCCTGCGGCATCAATTCCAAGTGGACCGTG
TGCACCAACGGCGAGCGCATCATCAAGTATCGGAATCCAGATAAGAACAATATGTTCGACGAGAAGGTGGTGGTG
GTGACCGATGAGATGAAGAACCTGTTTGAGCAGTACAAGATCCCCTATGAGGATGGCAGAAATGTGAAGGACATG
ATCATCAGCAACGAGGAGGCCGAGTTCTACCGGAGACTGTATAGGCTGCTGCAGCAGACCCTGCAGATGAGAAAC
AGCACCTCCGACGGCACAAGGGATTACATCATCTCCCCTGTGAAGAATAAGAGAGAGGCCTACTTCAACAGCGAG
CTGTCCGACGGCTCTGTGCCAAAGGACGCCGATGCCAACGGCGCCTACAATATCGCCAGAAAGGGCCTGTGGGTG
CTGGAGCAGATCAGGCAGAAGAGCGAGGGCGAGAAGATCAATCTGGCCATGACCAACGCCGAGTGGCTGGAGTAT
GCCCAGACACACCTGCTG

>ErCas12a

ATGAACAACGGCACAAACAACTTCCAGAACTTCATCGGGATATCTTCCCTTCAGAAAACCCTTCGAAATGCTTTG
ATTCCAACCGAAACGACCCAGCAATTCATCGTGAAGAACGGTATCATTAAAGAAGATGAACTGCGAGGCGAGAAT
AGGCAGATCTTGAAGGACATTATGGACGACTACTACCGAGGCTTTATCTCAGAGACACTGAGCAGTATTGACGAT
ATCGACTGGACTTCCCTTTTCGAAAAGATGGAAATTCAGCTCAAGAACGGGGACAATAAGGACACTCTGATTAAA
GAACAGACGGAGTATCGCAAGGCCATTCACAAGAAGTTCGCTAACGATGATAGATTCAAGAACATGTTCTCCGCC
AAACTGATTTCAGATATCTTGCCCGAATTCGTTATTCACAATAATAATTATTCAGCAAGTGAGAAGGAGGAGAAG
ACTCAAGTGATTAAGCTGTTTTCCAGATTCGCAACATCCTTCAAGGACTACTTCAAGAACCGAGCAAACTGTTTC
AGCGCCGATGACATTAGCAGTTCTTCATGTCATAGGATCGTCAATGACAATGCCGAAATTTTTTTAGCAACGCC
CTGGTGTACCGGAGAATTGTGAAGTCCCTGTCTAATGATGACATAAATAAGATTTCAGGAGATATGAAAGATAGT
CTTAAGGAGATGTCATTGGAGGAGATCTATTCCTACGAAAAATATGGCGAGTTTATCACACAGGAAGGGATCTCC
TTTTACAACGACATCTGTGGCAAGGTGAACAGCTTCATGAACCTTTATTGTCAGAAGAATAAAGAAAACAAAAAC
CTGTATAAACTCCAAAAGCTTCATAAGCAGATCCTTTGCATTGCTGATACGAGTTACGAGGTCCCTTACAAGTTC
GAGTCCGATGAGGAGGTGTATCAGAGCGTGAATGGGTTTCTCGATAATATCTCATCCAAACATATAGTGGAGCGC
CTGAGAAAGATTGGAGACAACTACAATGGCTACAATTTGGATAAAATCTACATTGTGTCTAAGTTCTATGAGTCA
GTGAGCCAGAAAACATATCGAGACTGGGAAACTATTAACACCGCTCTCGAAATCCATTACAATAACATCCTGCCC
GGCAACGGCAAATCTAAGGCAGATAAAGTCAAAAAGGCCGTAAAGAATGACCTGCAGAAGAGCATCACAGAAATC
AATGAGCTTGTGTCTAACTACAAACTTTGTTCCGACGACAATATTAAAGCCGAAACATATATACACGAAATTTCT

CACATCCTCAACAATTTCGAGGCCCAGGAACTTAAATACAACCCCGAGATCCACCTTGTAGAGAGCGAACTCAAA
GCCAGCGAACTCAAGAACGTCCTGGATGTGATTATGAACGCGTTTCACTGGTGCAGTGTCTTCATGACCGAGGAA
CTGGTGGACAAGGATAATAACTTTTATGCCGAACTGGAGGAAATTTATGACGAAATATATCCAGTCATATCTCTG
TACAACCTGGTTAGGAATTATGTCACACAGAAGCCTTACTCCACCAAAAAGATCAAACTGAACTTCGGCATCCCC
ACACTCGCCGATGGGTGGTCTAAAAGTAAGGAATACAGCAACAACGCAATAATTCTGATGAGGGACAACCTCTAC
TATCTGGGCATCTTCAATGCCAAGAATAAGCCTGACAAGAAAATTATTGAGGGCAACACGTCAGAGAATAAGGGG
GACTACAAGAAAATGATTTACAATCTGTTGCCCGGCCCAAACAAGATGATTCCTAAGGTGTTCCTCTCAAGCAAA
ACTGGAGTAGAAACATATAAGCCTAGCGCGTACATACTGGAGGGTTACAAGCAGAATAAGCATATTAAGTCAAGT
AAAGATTTCGATATCACATTCTGCCATGACCTGATCGATTACTTTAAAAATTGCATCGCAATACATCCCGAATGG
AAGAACTTCGGATTCGACTTTAGTGATACATCCACATATGAAGACATTTCCGGTTTCTATAGGGAGGTGGAGCTG
CAGGGATATAAGATCGATTGGACCTACATTTCAGAGAAAGACATTGACCTTCTGCAAGAAAAAGGACAGCTCTAT
CTCTTTCAGATTTACAATAAGGACTTTAGTAAAAAATCCACAGGGAACGACAATCTGCACACAATGTATCTTAAA
AACCTGTTTTCCGAGGAGAACTTGAAGGACATAGTGCTTAAGTTGAATGGGGAAGCCGAAATTTTTTTTAGAAAG
TCCAGTATTAAAAATCCTATAATCCATAAGAAAGGTTCAATCTTGGTGAACAGAACTTACGAGGCTGAGGAAAAG
GATCAGTTCGGGAATATACAGATTGTGAGAAAGAACATCCCGGAGAATATTTACCAGGAGCTTTACAAGTACTTC
AATGATAAGTCTGATAAGGAACTCAGCGACGAGGCCGCCAAATTGAAGAATGTGGTCGGACATCACGAAGCCGCC
ACAAATATCGTCAAAGACTACAGATATACTTACGATAAGTACTTCCTGCATATGCCAATAACTATCAACTTCAAG
GCTAATAAAACCGGTTTTATAAATGACCGGATTTTGCAGTATATTGCCAAAGAGAAAGATCTCCATGTGATCGGC
ATCGACCGCGGAGAGAGAAACCTCATTTACGTGTCTGTGATTGACACATGTGGGAATATCGTGGAACAGAAATCT
TTTAATATTGTGAATGGATATGATTACCAGATCAAGTTGAAGCAGCAGGAGGGGGCCCGCCAGATCGCCAGGAAG
GAATGGAAGGAAATCGGCAAGATTAAAGAAATCAAGGAAGGGTACCTGTCATTGGTTATTCACGAAATCAGCAAA
ATGGTAATCAAGTACAACGCCATCATTGCAATGGAGGACCTGAGCTACGGCTTTAAGAAGGGTCGCTTCAAAGTG
GAGCGCCAGGTGTACCAGAAGTTTGAAACCATGCTCATCAACAAACTCAATTATTTGGTGTTTAAGGACATTTCC
ATAACCGAAAACGGCGGTCTGTTGAAAGGGTACCAGCTGACCTACATCCCTGACAAGCTGAAGAACGTAGGGCAC
CAATGTGGGTGCATCTTTTATGTACCTGCCGCGTACACCTCTAAGATCGATCCGACAACCGGCTTTGTGAATATC
TTCAAGTTCAAGGATCTTACCGTGGATGCGAAGCGCGAGTTCATTAAAAAGTTTGATTCCATTAGATACGACTCC
GAGAAGAACCTTTTCTGCTTTACTTTTGATTACAACAATTTCATCACTCAAAACACTGTGATGAGTAAATCTTCC
TGGAGCGTGTACACATACGGCGTGCGAATTAAGCGCCGGTTCGTGAATGGCAGGTTCTCCAATGAGTCCGATACC
ATTGACATCACTAAAGACATGGAGAAGACCCTGGAGATGACTGATATTAATTGGAGGGACGGCCACGATCTGCGG
CAGGACATCATCGATTATGAGATCGTACAGCATATATTCGAGATCTTCCGCCTGACAGTGCAGATGAGGAACTCC
CTCAGCGAGCTTGAGGATCGCGACTATGATAGACTCATTTCTCCCGTCCTGAACGAGAATAATATTTTTTATGAT
TCTGCCAAGGCTGGCGATGCCCTGCCCAAGGACGCGGACGCAAACGGTGCCTACTGCATCGCTCTTAAGGGCTTG
TACGAGATTAAACAGATAACGGAGAACTGGAAGGAGGACGGTAAATTTTCTAGGGACAAGTTGAAAATTTCTAAT
AAGGACTGGTTTGATTTCATACAGAATAAAAGATACCTGTAA

>EeCas12a

ATGAACGGCAATAGGTCCATCGTGTACCGCGAGTTCGTGGGCGTGATCCCCGTGGCCAAGACCCTGAGGAATGAG
CTGCGCCCTGTGGGCCACACACAGGAGCACATCATCCAGAACGGCCTGATCCAGGAGGACGAGCTGCGGCAGGAG
AAGAGCACCGAGCTGAAGAACATCATGGACGATTACTATAGAGAGTACATCGATAAGTCTCTGAGCGGCGTGACC
GACCTGGACTTCACCCTGCTGTTCGAGCTGATGAACCTGGTGCAGAGCTCCCCCTCCAAGGACAATAAGAAGGCC
CTGGAGAAGGAGCAGTCTAAGATGAGGGAGCAGATCTGCACCCACCTGCAGTCCGACTCTAACTACAAGAATATC
TTTAACGCCAAGCTGCTGAAGGAGATCCTGCCTGATTTCATCAAGAACTACAATCAGTATGACGTGAAGGATAAG
GCCGGCAAGCTGGAGACACTGGCCCTGTTTAATGGCTTCAGCACATACTTTACCGACTTCTTTGAGAAGAGGAAG

AACGTGTTCACCAAGGAGGCCGTGAGCACATCCATCGCCTACCGCATCGTGCACGAGAACTCCCTGATCTTCCTG
GCCAATATGACCTCTTATAAGAAGATCAGCGAGAAGGCCCTGGATGAGATCGAAGTGATCGAGAAGAACAATCAG
GACAAGATGGGCGATTGGGAGCTGAATCAGATCTTTAACCCTGACTTCTACAATATGGTGCTGATCCAGTCCGGC
ATCGACTTCTACAACGAGATCTGCGGCGTGGTGAATGCCCACATGAACCTGTACTGTCAGCAGACCAAGAACAAT
TATAACCTGTTCAAGATGCGGAAGCTGCACAAGCAGATCCTGGCCTACACCAGCACCAGCTTCGAGGTGCCCAAG
ATGTTCGAGGACGATATGAGCGTGTATAACGCCGTGAACGCCTTCATCGACGAGACAGAGAAGGGCAACATCATC
GGCAAGCTGAAGGATATCGTGAATAAGTACGACGAGCTGGATGAGAAGAGAATCTATATCAGCAAGGACTTTTAC
GAGACACTGAGCTGCTTCATGTCCGGCAACTGGAATCTGATCACAGGCTGCGTGGAGAACTTCTACGATGAGAAC
ATCCACGCCAAGGGCAAGTCCAAGGAGGAGAAGGTGAAGAAGGCCGTGAAGGAGGACAAGTACAAGTCTATCAAT
GACGTGAACGATCTGGTGGAGAAGTATATCGATGAGAAGGAGAGGAATGAGTTCAAGAACAGCAATGCCAAGCAG
TACATCCGCGAGATCTCCAACATCATCACCGACACAGAGACAGCCCACCTGGAGTATGACGATCACATCTCTCTG
ATCGAGAGCGAGGAGAAGGCCGACGAGATGAAGAAGCGGCTGGATATGTATATGAACATGTACCACTGGGCCAAG
GCCTTTATCGTGGACGAGGTGCTGGACAGAGATGAGATGTTCTACAGCGATATCGACGATATCTATAATATCCTG
GAGAACATCGTGCCACTGTATAATCGGGTGAGAAACTACGTGACCCAGAAGCCCTACAACTCTAAGAAGATCAAG
CTGAATTTCCAGAGCCCTACACTGGCCAATGGCTGGTCCCAGTCTAAGGAGTTCGACAACAATGCCATCATCCTG
ATCAGAGATAACAAGTACTATCTGGCCATCTTCAATGCCAAGAACAAGCCAGACAAGAAGATCATCCAGGGCAAC
TCCGATAAGAAGAACGACAACGATTACAAGAAGATGGTGTATAACCTGCTGCCAGGCGCCAACAAGATGCTGCCC
AAGGTGTTTCTGTCTAAGAAGGGCATCGAGACATTCAAGCCCTCCGACTATATCATCTCTGGCTACAACGCCCAC
AAGCACATCAAGACAAGCGAGAATTTTGATATCTCCTTCTGTCGGGACCTGATCGATTACTTCAAGAACAGCATC
GAGAAGCACGCCGAGTGGAGAAAGTATGAGTTCAAGTTTTCCGCCACCGACAGCTACTCCGATATCTCTGAGTTC
TATCGGGAGGTGGAGATGCAGGGCTACAGAATCGACTGGACATATATCAGCGAGGCCGACATCAACAAGCTGGAT
GAGGAGGGCAAGATCTATCTGTTTCAGATCTACAATAAGGATTTCGCCGAGAACAGCACCGGCAAGGAGAATCTG
CACACAATGTACTTTAAGAACATCTTCTCCGAGGAGAATCTGAAGGACATCATCATCAAGCTGAACGGCCAGGCC
GAGCTGTTTTATCGGAGAGCCTCTGTGAAGAATCCCGTGAAGCACAAGAAGGATAGCGTGCTGGTGAACAAGACC
TACAAGAATCAGCTGGACAACGGCGACGTGGTGAGAATCCCCATCCCTGACGATATCTATAACGAGATCTACAAG
ATGTATAATGGCTACATCAAGGAGTCCGACCTGTCTGAGGCCGCCAAGGAGTACCTGGATAAGGTGGAGGTGAGG
ACCGCCCAGAAGGACATCGTGAAGGATTACCGCTATACAGTGGACAAGTACTTCATCCACACACCTATCACCATC
AACTATAAGGTGACCGCCCGCAACAATGTGAATGATATGGTGGTGAAGTACATCGCCCAGAACGACGATATCCAC
GTGATCGGCATCGACCGGGGCGAGAGAAACCTGATCTACATCTCCGTGATCGATTCTCACGGCAACATCGTGAAG
CAGAAATCCTACAACATCCTGAACAACTACGACTACAAGAAGAAGCTGGTGGAGAAGGAGAAAACCCGGGAGTAC
GCCAGAAAGAACTGGAAGAGCATCGGCAATATCAAGGAGCTGAAGGAGGGCTATATCTCCGGCGTGGTGCACGAG
ATCGCCATGCTGATCGTGGAGTACAACGCCATCATCGCCATGGAGGACCTGAATTATGGCTTTAAGAGGGGCCGC
TTCAAGGTGGAGCGGCAGGTGTACCAGAAGTTTGAGAGCATGCTGATCAATAAGCTGAACTATTTCGCCAGCAAG
GAGAAGTCCGTGGACGAGCCAGGAGGCCTGCTGAAGGGCTATCAGCTGACCTACGTGCCCGATAATATCAAGAAC
CTGGGCAAGCAGTGCGGCGTGATCTTTTACGTGCCTGCCGCCTTCACCAGCAAGATCGACCCATCCACAGGCTTT
ATCTCTGCCTTCAACTTTAAGTCTATCAGCACAAATGCCTCTCGGAAGCAGTTCTTTATGCAGTTTGACGAGATC
AGATACTGTGCCGAGAAGGATATGTTCAGCTTTGGCTTCGACTACAACAACTTCGATACCTACAACATCACAATG
GGCAAGACACAGTGGACCGTGTATACAAACGGCGAGAGACTGCAGTCTGAGTTCAACAATGCCAGGCGCACCGGC
AAGACAAAGAGCATCAATCTGACAGAGACAATCAAGCTGCTGCTGGAGGACAATGAGATCAACTACGCCGACGGC
CACGATATCAGGATCGATATGGAGAAGATGGACGAGGATAAGAAGAGCGAGTTCTTTGCCCAGCTGCTGAGCCTG
TATAAGCTGACCGTGCAGATGCGCAATTCCTATACAGAGGCCGAGGAGCAGGAGAACGGCATCTCTTACGACAAG
ATCATCAGCCCTGTGATCAATGATGAGGGCGAGTTCTTTGACTCCGATAACTATAAGGAGTCTGACGATAAGGAG
TGCAAGATGCCAAAGGACGCCGATGCCAACGGCGCCTACTGTATCGCCCTGAAGGGCCTGTATGAGGTGCTGAAG

ATCAAGAGCGAGTGGACCGAGGACGGCTTTGATAGGAATTGCCTGAAGCTGCCACACGCAGAGTGGCTGGACTTC
ATCCAGAACAAGCGGTACGAG

>CeCas12a

ATGAACAACAACACCAACAACAGCTTCGAGCCCTTCATCGGCGGCAACAGCGTGAGCAAGACCCTGCGCAACGAG
CTGCGCGTGGGCAGCGAGTACACCGGCAAGCACATCAAGGAGTGCGCCATCATCGCCGAGGACGCCGTGAAGGCC
GAGAACCAGTACATCGTGAAGGAGATGATGGACGACTTCTACCGCGACTTCATCAACCGCAAGCTGGACGCCCTG
CAGGGCATCAACTGGGAGCAGCTGTTCGACATCATGAAGAAGGCCAAGCTGGACAAGAGCAACAAGGTGAGCAAG
GAGCTGGACAAGATCCAGGAGAGCACCCGCAAGGAGATCGTGAAGATCTTCAGCAGCGACCCCATCTACAAGGAC
ATGCTGAAGGCCGACATGATCAGCAAGATCCTGCCCGAGTACATCGTGGACAAGTACGGCGACGCCGCCAGCCGC
ATCGAGGCCGTGAAGGTGTTCTACGGCTTCAGCGGCTACTTCATCGACTTCTGGGCCAGCCGCAAGAACGTGTTC
AGCGACAAGAACATCGCCAGCGCCATCCCCCACCGCATCGTGAACGTGAACGCCCGCATCCACCTGGACAACATC
ACCGCCTTCAACCGCATCGCCGAGATCGCCGGCGACGAGGTGGCCGGCATCGCCGAGGACGCCTGCGCCTACCTG
CAGAACATGAGCCTGGAGGACGTGTTCACCGGCGCCTGCTACGGCGAGTTCATCTGCCAGAAGGACATCGACCGC
TACAACAACATCTGCGGCGTGATCAACCAGCACATGAACCAGTACTGCCAGAACAAGAAGATCAGCCGCAGCAAG
TTCAAGATGGAGCGCCTGCACAAGCAGATCCTGTGCCGCAGCGAGAGCGGCTTCGAGATCCCCATCGGCTTCCAG
ACCGACGGCGAGGTGATCGACGCCATCAACAGCTTCAGCACCATCCTGGAGGAGAAGGACATCCTGGACCGCCTG
CGCACCCTGAGCCAGGAGGTGACCGGCTACGACATGGAGCGCATCTACGTGAGCAGCAAGGCCTTCGAGAGCGTG
AGCAAGTACATCGACCACAAGTGGGACGTGATCGCCAGCAGCATGTACAACTACTTCAGCGGCGCCGTGCGCGGC
AAGGACGACAAGAAGGACGCCAAGATCCAGACCGAGATCAAGAAGATCAAGAGCTGCAGCCTGCTGGACCTGAAG
AAGCTGGTGGACATGTACTACAAGATGGACGGCATGTGCCTGGAGCACGAGGCCACCGAGTACGTGGCCGGCATC
ACCGAGATCCTGGTGGACTTCAACTACAAGACCTTCGACATGGACGACAGCGTGAAGATGATCCAGAACGAGCAC
ATGATCAACGAGATCAAGGAGTACCTGGACACCTACATGAGCATCTACCACTGGGCCAAGGACTTCATGATCGAC
GAGCTGGTGGACCGCGACATGGAGTTCTACAGCGAGCTGGACGAGATCTACTACGACCTGAGCGACATCGTGCCC
CTGTACAACAAGGTGCGCAACTACGTGACCCAGAAGCCCTACAGCCAGGACAAGATCAAGCTGAACTTCGGCAGC
CCCACCCTGGCCAACGGCTGGAGCAAGAGCAAGGAGTTCGACAACAACGTGGTGGTGCTGCTGCGCGACGAGAAG
ATCTACCTGGCCATCCTGAACGTGGGCAACAAGCCCAGCAAGGACATCATGGCCGGCGAGGACCGCCGCCGCAGC
GACACCGACTACAAGAAGATGAACTACTACCTGCTGCCCGGCGCCAGCAAGACCCTGCCCCACGTGTTCATCAGC
AGCAACGCCTGGAAGAAGAGCCACGGCATCCCCGACGAGATCATGTACGGCTACAACCAGAACAAGCACCTGAAG
AGCAGCCCCAACTTCGACCTGGAGTTCTGCCGCAAGCTGATCGACTACTACAAGGAGTGCATCGACAGCTACCCC
AACTACCAGATCTTCAACTTCAAGTTCGCCGCCACCGAGACCTACAACGACATCAGCGAGTTCTACAAGGACGTG
GAGCGCCAGGGCTACAAGATCGAGTGGAGCTACATCAGCGAGGACGACATCAACCAGATGGACCGCGACGGCCAG
ATCTACCTGTTCCAGATCTACAACAAGGACTTCGCCCCCAACAGCAAGGGCATGCAGAACCTGCACACCCTGTAC
CTGAAGAACATCTTCAGCGAGGAGAACCTGAGCGACGTGGTGATCAAGCTGAACGGCGAGGCCGAGCTGTTCTTC
CGCAAGAGCAGCATCCAGCACAAGCGCGGCCACAAGAAGGGCAGCGTGCTGGTGAACAAGACCTACAAGACCACC
GAGAAGACCGAGAACGGCCAGGGCGAGATCGAGGTGATCGAGAGCGTGCCCGACCAGTGCTACCTGGAGCTGGTG
AAGTACTGGAGCGAGGGCGGCGTGGGCCAGCTGAGCGAGGAGGCCAGCAAGTACAAGGACAAGGTGAGCCACTAC
GCCGCCACCATGGACATCGTGAAGGACCGCCGCTACACCGAGGACAAGTTCTTCATCCACATGCCCATCACCATC
AACTTCAAGGCCGACAACCGCAACAACGTGAACGAGAAGGTGCTGAAGTTCATCGCCGAGAACGACGACCTGCAC
GTGATCGGCATCGACCGCGGCGAGCGCAACCTGCTGTACGTGAGCGTGATCGACAGCCGCGGCCGCATCGTGGAG
CAGAAGAGCTTCAACATCGTGGAGAACTACGAGAGCAGCAAGAACGTGATCCGCCGCCACGACTACAAGGGCAAG
CTGGTGAACAAGGAGCACTACCGCAACGAGGCCCGCAAGAGCTGGAAGGAGATCGGCAAGATCAAGGAGATCAAG
GAGGGCTACCTGAGCCAGGTGATCCACGAGATCAGCAAGCTGGTGCTGAAGTACAACGCCATCATCGTGATGGAG

GACCTGAACTACGGCTTCAAGCGCGGCCGCTTCAAGGTGGAGCGCCAGGTGTACCAGAAGTTCGAGACCATGCTG
ATCAACAAGCTGGCCTACCTGGTGGACAAGAGCCGCGCCGTGGACGAGCCCGGCGGCCTGCTGAAGGGCTACCAG
CTGACCTACGTGCCCGACAACCTGGGCGAGCTGGGCAGCCAGTGCGGCATCATCTTCTACGTGCCCGCCGCCTAC
ACCAGCAAGATCGACCCCGTGACCGGCTTCGTGGACGTGTTCGACTTCAAGGCCTACAGCAACGCCGAGGCCCGC
CTGGACTTCATCAACAAGCTGGACTGCATCCGCTACGACGCCAGCCGCAACAAGTTCGAGATCGCCTTCGACTAC
GGCAACTTCCGCACCCACCACACCACCCTGGCCAAGACCAGCTGGACCATCTTCATCCACGGCGACCGCATCAAG
AAGGAGCGCGGCAGCTACGGCTGGAAGGACGAGATCATCGACATCGAGGCCCGCATCCGCAAGCTGTTCGAGGAC
ACCGACATCGAGTACGCCGACGGCCACAACCTGATCGGCGACATCAACGAGCTGGAGAGCCCCATCCAGAAGAAG
TTCGTGGGCGAGCTGTTCGACATCATCCGCTTCACCGTGCAGCTGCGCAACAGCAAGAGCGAGAAGTACGACGGC
ACCGAGAAGGAGTACGACAAGATCATCAGCCCCGTGATGGACGAGGAGGGCGTGTTCTTCACCACCGACAGCTAC
ATCCGCGCCGACGGCACCGAGCTGCCCAAGGACGCCGACGCCAACGGCGCCTACTGCATCGCCCTGAAGGGCCTG
TACGACGTGCTGGCCGTGAAGAAGTACTGGAAGGAGGGCGAGAAGTTCGACCGCAAGCTGCTGGCCATCACCAAC
TACAACTGGTTCGACTTCATCCAGAACCGCCGCTTC

For the four Cas12a variants, R substitutions were performed by replacing the codons at the corresponding amino acid positions of the wild-type protein with the codon for arginine (AGA), while the A substitution was performed by replacing the codon at the corresponding position with the codon for alanine (GCC).

>enAsCas12a-HF(E174R/N282A/S542R/K548R)

We deposited the three novel engineered 3R versions of the Cas12a variants in Addgene under the ID numbers shown below:

>PrCas12a-3Rv(E162R/N519R/K525R)   Addgene#179708
>Mb3Cas12a-3Rv(D180R/N581R/K587R) Addgene#179709
>BsCas12a-3Rv(K155R/N512R/K518R)   Addgene#179710

Detailed Legend for Figures 1-5

Here, we provide supplementary details for the legends of Figures 1-5, including the targets involved, the statistical analysis used, detailed *p* values, and other descriptions.

Related to Figure 1

(**d**) Synt.R1 and R2 show the target sites from synthetic biological replicates. ‘D12’ represents a 12 bp deletion repair outcome generated by Cas12a editing after alignment with the original target site

sequence. The repair outcome containing bases shown in bold red text denoting the microhomology unit (MH unit) indicates that the repair outcome was generated via MMEJ-based pathway.

Related to Figure 2

(**b**) Note that the PrCas12a bacterial loci contain no DR array. Here, we applied an optimized crRNA scaffold sequence with a loop region variant as UAUG for the editing activity assay of PrCas12a. Box-whisker plots: median line, quartiles for box edges, 1-99% whiskers. Differences in the editing activity between Cas12a orthologues were determined by non-parametric Kruskal–Wallis test (H test), followed by Dunn's multiple comparison post hoc test. ****$p < 0.0001$. For p values less significant than **** p <0.0001, (**c**) HEK293T: Pr v.s. Bs, ** p =0.0062; Lb v.s. Lb2, *** p =0.0002. (**d**) RKO: Mb3 v.s. Lb, ** p =0.0027.

Related to Figure 3

(**a**) The numbers of the target sites with diverse PAM sequences involved in each group are as follows. **AsCas12a**: TTTV, n=1,240; VTTV, n=457; TTCV, n=833; and TRTV, n=3,512. **BsCas12a**: TTTV, n=1,640; VTTV, n=348; TTCV, n=501; and TRTV, n=1,875. **PrCas12a**: TTTV, n=2,635; VTTV, n=300; TTCV, n=482; and TRTV, n=2,770. **Mb3Cas12a**: TTTV, n=2,844; VTTV, n=314; TTCV, n=386; and TRTV, n=3,169. Box-whisker plots: median line, quartiles for box edges, 1-99% whiskers. V = A/C/G; R = A/G. (**b**) The targeted DNA contains the target site with a TTTA PAM; the 4 nt PAM parts of the non-target strand and target strand DNA are highlighted in red and yellow, respectively, marked with the corresponding bases and positions in PAM, such as NT(-1)DA, representing an adenine nucleotide at position −1 in the 4 nt PAM on the non-target strand. Ten amino acid residues proximal to PAM are highlighted in blue. Substitutions of these residues were expected to alter the PAM recognition pattern of Cas12a. (**c**) Based on an amino acid alignment with AsCas12a, we introduced the three arginine substitutions mentioned above into the corresponding residue positions of three Cas12a orthologues showing potential in our editing activity assessment (BsCAs12a, PrCas12a, and Mb3Cas12a). Three Cas12a-3R variants were generated: BsCas12a-3Rv (K155R/N512R/K518R), PrCas12a-3Rv (E162R/N519R/K525R), and Mb3Cas12a-3Rv (D180R/N581R/K587R).

Related to Figure 4

The same set of target sites were chosen to evaluate the editing efficiency between WT Cas12a and engineered Cas12a mutants. The number of target sites with diverse PAM sequences involved in each group is as follows. (**a**) PrCas12a: TTTV, n=2,635; VTTV, n=300; TTCV, n=482; and TRTV, n=2,770. (**b**) Mb3Cas12a: TTTV, n=2,844; VTTV, n=314; TTCV, n=386; and TRTV, n=3,169. (**c**) BsCas12a: TTTV, n=1,640; VTTV, n=348; TTCV, n=501; and TRTV, n=1,875. (**d**) AsCas12a: TTTV, n=1,240; VTTV, n=457; TTCV, n=833; and TRTV, n=3,512. Box-whisker plots: median line, quartiles for box edges, 1-99% whiskers. **** $p < 0.0001$ (Mann–Whitney,

two-tailed). V=A/C/G; R=A/G.

Related to Figure 5

Target sites involved in Figure 5 had at least 300 read counts per crRNA–target pair in one sample. The number of target sites involved in each group is as follows. (b) PrCas12a-3Rv: TGTV, *n*=1,249; TATV, *n*=715. Mb3Cas12a-3Rv: TGTV, *n*=840; TATV: *n*=1,250. BsCas12a-3Rv: TGTV, *n*=995; TATV, *n*=605. enAsCas12a-HF: TGTV, *n*=1,500; TATV, *n*=1,437. (c) PrCas12a-3Rv: TTCV, *n*=1,019; ATCV, *n*=436; CTCV, *n*=541; and GTCV, *n*=482. Mb3Cas12a-3Rv: TTCV, *n*=1,095; ATCV, *n*=252; CTCV, *n*=500; and GTCV, *n*=464. Box-whisker plots: median line, quartiles for box edges, 1-99% whiskers. Differences in the editing activity between groups were determined by non-parametric Kruskal–Wallis test (H test), followed by Dunn's multiple comparison post hoc test. **** p <0.0001. For p values less significant than **** p <0.0001, (b) Mb3Cas12a-3Rv: TGTV vs. TATV, (ns) p =0.0750; enAsCas12a-HF: TGTV vs. TATV, (ns) p =0.0684. (c) PrCas12a-3Rv: ATCV vs. CTCV, (ns) p =0.2305; ATCV vs. GTCV, (ns) p >0.9999. Mb3Cas12a-3Rv: TTCV vs. ATCV, (ns) p >0.9999; CTCV vs. GTCV, (ns) p =0.6449.
